# Decoding the Transcriptomics of Oil Palm Seed Germination

**DOI:** 10.3390/plants13192680

**Published:** 2024-09-24

**Authors:** Padungsak Suksa-Ard, Sunya Nuanlaong, Chettupon Pooljun, Azzreena Mohamad Azzeme, Potjamarn Suraninpong

**Affiliations:** 1 School of Agricultural Technology and Food Industry, Walailak University, Nakhon Si Thammarat 80161, Thailand; spadungs@wu.ac.th (P.S.-A.); sunya.ton@gmail.com (S.N.); 2 Akkhraratchakumari Veterinary College, Walailak University, Nakhon Si Thammarat 80160, Thailand; chettupon.po@wu.ac.th; 3 Faculty of Biotechnology and Biomolecular Sciences, Universiti Putra Malaysia, Serdang 43400, Malaysia; azzreena@upm.edu.my; 4 Biomass and Oil Palm Center of Excellence, Walailak University, Nakhon Si Thammarat 80161, Thailand

**Keywords:** seed dormancy, germination, hormonal signaling, starch metabolism

## Abstract

Seed dormancy and germination are critical factors affecting oil palm production efficiency. The typical dormancy-breaking process involves dry heat treatment (38–40 °C for 40–60 days) followed by germination at 30–32 °C. To understand the molecular mechanisms behind this process and improve germination rates and speed, we conducted transcriptome analysis at three stages: pre-incubation, 45-day incubation at 40 °C, and 14-day germination at 32 °C. Our findings, supported by qRT–PCR and DEGs analysis, identified four key stages: ABA degradation, energy mobilization, starch mobilization, and cell elongation and division. ABA pathway genes (*SnRK2*, *PYR*/*PYL*) were active during dormancy release, while *GAE* and *GPI* were upregulated after heat treatment, indicating increased energy metabolism and structural changes. During germination, genes involved in starch/sucrose metabolism (*SPS*, *TPP*, *SS*, *MGAM*) and cell wall biosynthesis (*GAUT1*, *PE*, *GAE*) supported embryo expansion, with *BAM*, *PGM*, *GlgB* fueling early growth. Auxin (*TIR1*, *AUX*/*IAA*, *ARF*), brassinosteroid (*BRI1*, *BSK*, *BIN2*, *CYCD3*), ethylene (*ETR*, *CTR1*), and jasmonic acid (*JAR1*, *COI1*) pathway genes regulated cell growth and stress response, promoting seedling development. Though gibberellins were not crucial for this oil palm variety, gene expression varied between varieties. This study provides information on oil palm seed germination that could be applied to other oil palm species, particularly in terms of incubation times and chemical treatments.

## 1. Introduction

Seed germination involves intricate processes leading to the growth of young plants. It is influenced by factors such as seed viability, water, oxygen, temperature, and light. In contrast, the phenomenon of seed dormancy represents a robust feature, hindering the sprouting of viable seeds due to factors like hard seed coats, reduced oxygen, underdeveloped embryos, or inhibitory chemicals [1]. A prominent illustration of this dormancy is evident in oil palm seeds, where approximately 50% of them undergo a prolonged delay in germination, lasting 5–6 months. These seeds exhibit a formidable combination of a tough shell (endocarp) and endosperm, wherein each of the three germ pores is obstructed by a 3 mm fiber plug firmly attached at the base, forming a plate-like structure known as the “operculum”. The operculum plays a pivotal role as a key factor in reinforcing and sustaining oil palm seed dormancy [2,3,4].

Breaking oil palm seed dormancy involves two main processes. Firstly, dry heat treatment at 38–40 °C is conducted at an appropriate moisture content, spanning 40–60 days, with the duration depending on seed size and shell thickness. Secondly, germination is achieved at 30–32 °C, resulting in a germination rate of approximately 85–90% [5]. It was reported that a 37-day heat treatment promotes a 90% germination rate [6], and 60 days of heat treatment at 40 °C enhances germination and growth [7,8]. In the case of seeds stored at 30 °C, germination begins after 112 days of heat treatment at 40 °C, while seeds stored at 40 °C initiate germination after 30 days [9]. Interestingly, removing the operculum without heat results in the highest germination rate at 42 days, reaching 80.66%. This outperforms operculum removal with 40 °C heat for one or two months and is statistically similar to 60 days of heat treatment at 40 °C, which yields 85.17% germination [10]. Importantly, operculum removal through the germ pore leads to rapid germination (90%) within just five days at 30 °C, surpassing the 40 °C heat treatment, which requires thirty-five days to achieve 80% germination, whether or not thiamethoxam and imidacloprid are used [11,12]. It is worth noting that germ pore operculum removal requires skill to prevent embryo damage. Given the critical need for the consistent germination of oil palm seeds, which is highly sought after by companies that sell germinated seeds and seedlings, these companies must ensure simultaneous germination and also be able to delay germination until orders are received. Consequently, this study focuses on investigating the genes involved in the germination process of oil palm under conditions of heat treatment.

The realm of biological omics data, spanning genomics, transcriptomics, proteomics, and metabolomics, continues to expand with the advancements facilitated by next-generation sequencing (NGS) technology. Cutting-edge platforms such as Roche/GS—FLX 454 Genome sequencer, ABI/SOLiD 5500xl, and Illumina/Hiseq 2000 play a pivotal role in unveiling genetic secrets and providing valuable insights into evolutionary processes [13]. Transcriptome research, a crucial component of this expansive field, encompasses all RNA types. The expression levels of these RNA molecules exhibit dynamic variations across tissues, growth stages, developmental phases, and in response to diverse environmental conditions [14].

Transcriptomic studies on seed dormancy began over a decade ago, shedding light on the complex mechanisms that regulate this process. In wheat, transcriptome analysis was conducted during seed germination of the elite Chinese bread wheat cultivar Jimai 20 [15]. Similarly, in maize, a dynamic transcriptome landscape was explored to understand embryo and endosperm development [16]. in soybean, germination studies emphasized the early activation of key metabolic and hormonal pathways [17]. *Paeonia lactiflora* transcriptomic research focused on the effects of alternating temperatures in breaking epicotyl morphophysiological dormancy in seeds [18]. For Callery pear (*Pyrus calleryana* Decne), transcriptome analysis during cold stratification revealed important genetic responses [19]. In *Polygonatum sibiricum*, a combination of SMRT and Illumina sequencing was utilized to study seed dormancy during warm and cold stratifications [20]. Transcriptomic analysis in ornamental peaches cv. “Yaguchi” (*Prunus persica*) following rinsing and chilling treatments identified genes linked to improved germination [21]. In rice, 14 transcripts and 15 metabolites were identified as crucial for germination and seedling growth [22]. Research on *Paris polyphylla* employed multiomics strategies to provide deeper insights into the mechanisms controlling dormancy release [23]. Lastly, in *Camellia oleifera*, transcriptomic analysis highlighted functional changes in cotyledonary tissues during seed germination and seedling emergence [24]. 

Building on previous successes, this study utilized next-generation sequencing (NGS) to identify differentially expressed genes (DGEs) across three key germination stages, in conjunction with quantitative reverse transcription PCR (qRT–PCR), to investigate the germination process of oil palm seeds. The stages examined were before incubation, after 45 days of incubation at 40 °C, and after germination following 14 days of incubation at 32 °C. Our results provide valuable insights for the application of hormones or chemicals to enhance the uniformity of seed germination and seedling production for commercial purposes.

## 2. Results

### 2.1. Transcriptome Sequencing and De Novo Assembly

The RNA from each sample was combined to create pooled cDNA samples. Enriched mRNA was isolated from the total RNA and converted into double-stranded RNA before library preparation and sequencing. The raw data from each library underwent quality control analysis. The error rate for each library, determined using the Phred score (Qphred = −10log10(e)) with Illumina CASAVA v1.8 software, was low, at just 0.01 percent, with Q20 and Q30 values ranging from 97.84 to 98.16 percent and 94.37 to 95.01 percent, respectively. The GC content distribution of each library, based on CG/AT segregation evaluation, fell within the range from 49.29 to 51.43 percent. Each library produced raw reads ranging from 39 to 49 million reads. Following read classification, adapter filtering, and the removal of low-quality reads, 38–47 million clean reads were obtained (Table 1).

From the clean reads, a de novo transcriptome was assembled using the Trinity program. The data were then corseted through hierarchical clustering to eliminate redundancy, resulting in the longest transcript data. Each group represented a unigene, and the analysis revealed a total of 148,695 unigenes, with the majority falling within the length range of approximately 500–1 kilobase pairs (46,360 unigenes), followed by 200–500 base pairs (40,688 unigenes). The average unigene length was approximately 1230 base pairs (Supplementary Table S1 and Figure S1).

### 2.2. Functional Annotations of Unigenes

**Figure 1:**
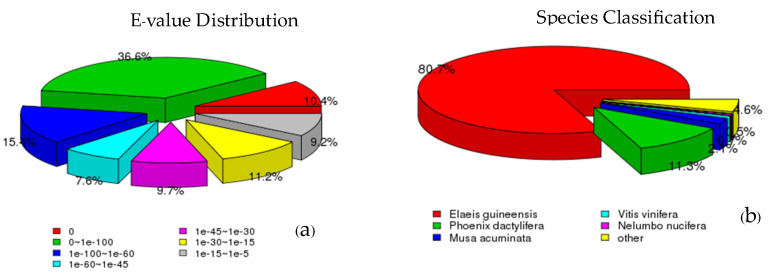
The distribution of unigenes in the NR database. (**a**) E-value distribution. (**b**) Species distribution.

Among the 148,695 unigenes, 84,253 unigenes (56.66%) were annotated in the NCBI nucleotide sequence (Nt) database, followed by the NCBI nonredundant protein (NR) database (74,595 unigenes, 50.16%). The ratio of annotated unigenes was similar for the Swiss-Prot (52,250, 35.13%), PFAM (53,987, 36.30%), and GO (54,064, 36.35%) databases. Meanwhile, the KOG (23,333, 15.69%) database annotated the fewest unigenes (Table 2, Supplementary Figure S2). A total of 13,779 unigenes (9.29%) matched entries in seven databases. The comparison of the 148,695 unigenes with the NR database revealed that most displayed an E-value of 0~1 × 10^−100^ (36.60%), followed by 1 × 10^−100^~1 × 10^−60^ (15.40%) and 10^−30^~10^−15^ (11.20%), respectively. The NR annotation species distribution analysis revealed homology with sequences of *E*. *guineensis* (80.70%), *Phoenix dactylifera* (11.30%), and *Musa acuminta* (2.10%) (Figure 1).

**Figure 2:**
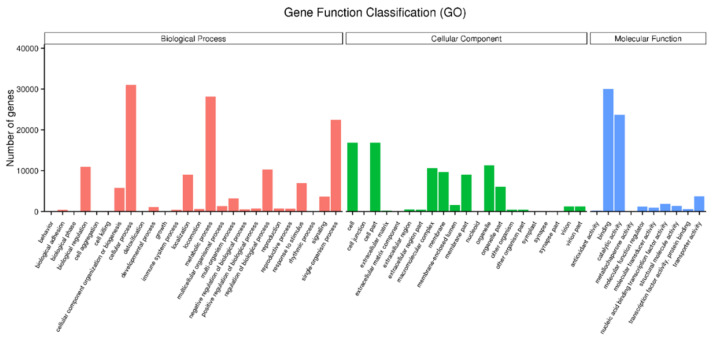
Gene ontology classification of unigenes at the secondary level.

In GO function classification, 148,695 unigenes were categorized into three major groups: biological processes, molecular functions, and cellular components. Within the biological process category, a significant number of unigenes were associated with cellular processes (31,028 unigenes), metabolic processes (28,186 unigenes), and single-organism processes (22,492 unigenes). In the molecular function category, the majority of genes were involved in binding (30,031 unigenes), followed by catalytic activity and transporter activity. In the cellular component category, cells (16,863 unigenes) were the most prevalent, followed by cell parts and macromolecular complexes (Figure 2).

**Figure 3:**
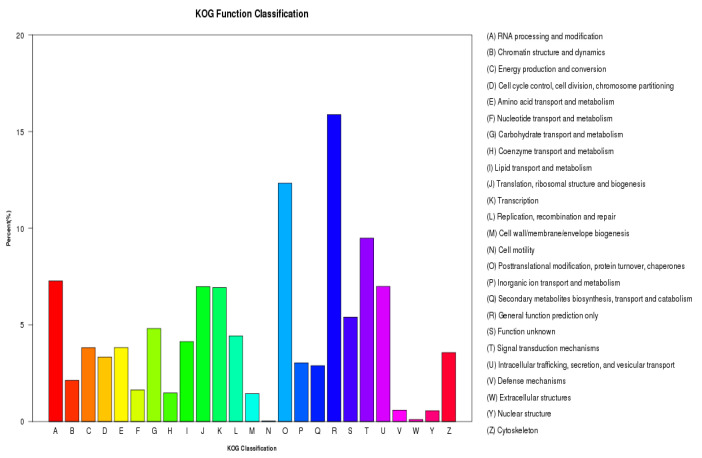
KOG function classification of unigenes.

Furthermore, 27,273 unigenes were annotated through KOG function classification, which categorizes them as orthologs or paralogs of eukaryotic proteins. Among these, the most prevalent category was “general function prediction only” (3704 unigenes), followed by “posttranslational modification, protein turnover, chaperones” (2887 unigenes) and “signal transduction mechanisms” (2213 unigenes) (Figure 3).

**Figure 4:**
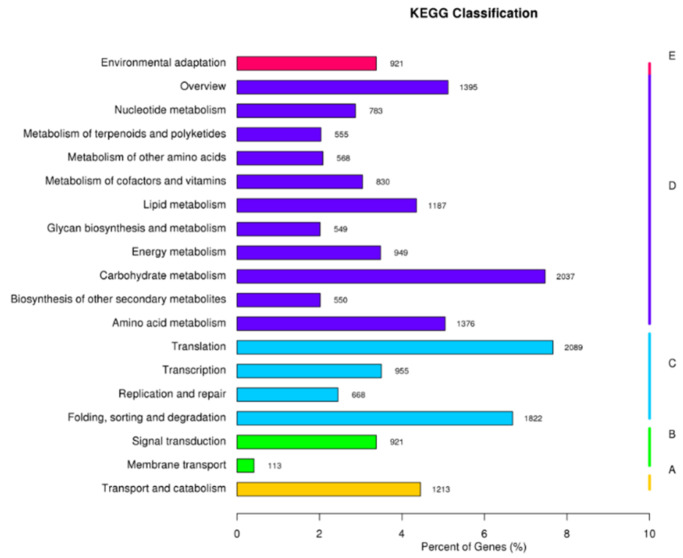
KEGG function classification of unigenes. (A) Cellular processes. (B) Environmental information processing. (C) Genetic information processing. (D) Metabolism. (E) Organismal systems.

The unigenes were subsequently mapped to the KEGG pathway database to identify biological pathways. In total, 22,273 unigenes were categorized into 19 secondary pathways within 5 primary pathways. The majority of these pathways were related to metabolism (11 pathways), followed by genetic information processing (4 pathways). Among these unigenes, the “translation” category had the highest number of unigenes (2089), followed by “carbohydrate metabolism” (2037). The “folding, sorting, and degradation” category was the smallest, comprising 1822 unigenes (Figure 4).

### 2.3. Gene Expression Difference Analysis

When analyzing de novo transcriptome data, the RSEM program, using Corset as a reference genome, aligned clean reads, counted the genes in each sample, and converted the values to FPKM (fragments per kilobase of transcript sequence per million base pairs sequenced). FPKM is a standard method for normalizing RNA-seq data and assessing gene expression levels. The analysis indicated that, on average, 74–75 percent of the total reads in each sample were successfully mapped to the reference data. Notably, the H_em sample showed the highest mapping rate (Table 3, Supplementary Figure S3)

**Figure 5:**
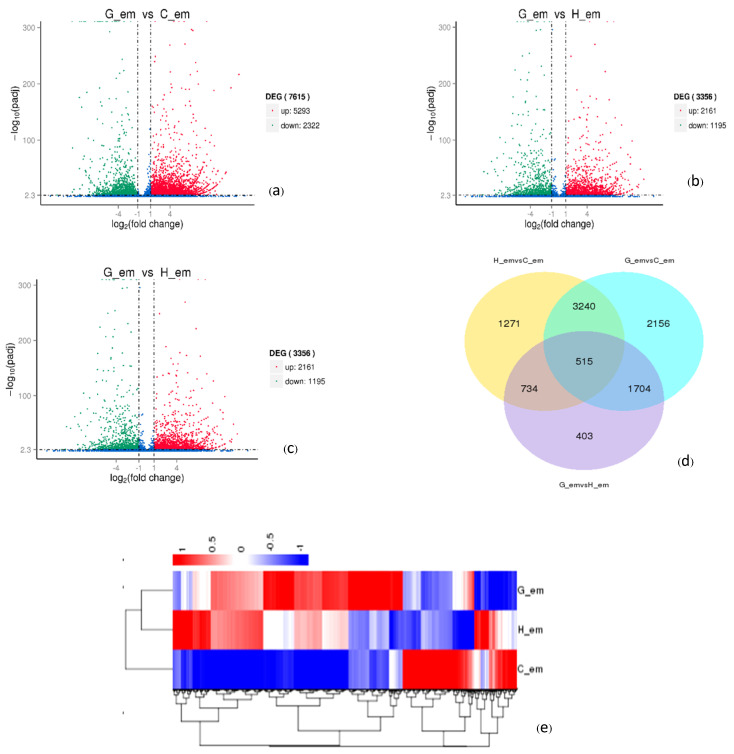
The differentially expressed gene analysis at each stage of oil palm seed germination. (**a**–**c**) Volcano plots. (**d**) A Venn diagram. (**e**) Gene cluster analysis was performed for each sample using FPKM values, with high-expression genes marked in red and low-expression genes marked in blue. C_em = seeds before incubation; G_em = seeds after 40 °C incubation for 45 days; H_em = germinated seedlings after 32 °C incubation for 14 days.

In the gene expression analysis, the results were used to identify differentially expressed genes (DEGs). It was observed that the G_em/C_em comparison had the highest number of DEGs, with a total of 7615 genes, the majority of which were up-regulated (5293 genes). In contrast, H_em/C_em had 5760 DEGs, and G_em/H_em had the fewest DEGs, totaling 3356 genes (Figure 5a–c). Additionally, there were 515 DEGs that were shared across all samples. However, when these DEGs were visualized in a Venn diagram, 2156 genes were specifically found in the G_em/C_em comparison. (Figure 5d).

When analyzing groups of DEGs to ascertain similarity patterns, genes with similar expression patterns were grouped together to explore potential functions and roles of unknown genes. These groups were differentiated by color. Genes within the same color group were likely to share similar functions or participate in common biological processes. The analysis identified three distinct groups of genes with varying expression patterns. Notably, H_em displayed different expression patterns of genes that were more closely related to the G_em group than the C_em group, for instance (Figure 5e).

### 2.4. GO Enrichment Analysis of DEGs

**Figure 6:**
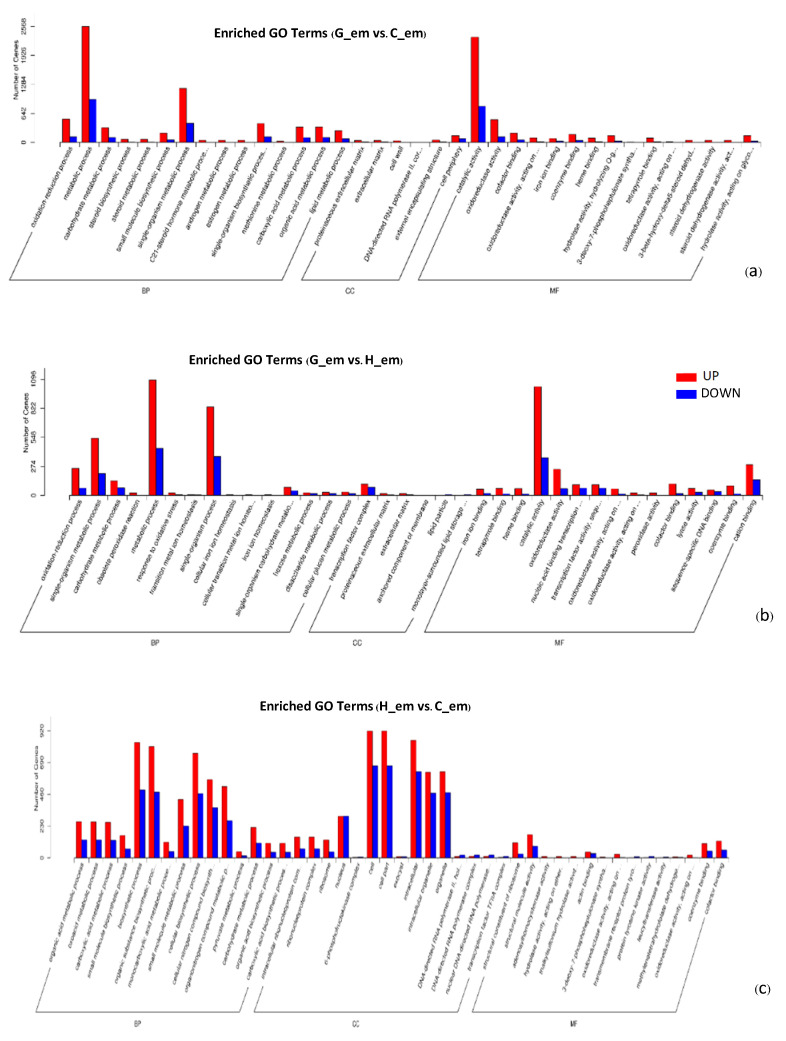
GO terms of genes differentially expressed between oil palm seed germination stages. (**a**) Enriched GO terms of G_em vs. C_em. (**b**) Enriched GO terms of G_em vs. H_em. (**c**) Enriched GO terms of H_em vs. G_em (BP = biological process; CC = cellular component; MF = molecular function; C_em = seeds before incubation; G_em = seeds after 40 °C incubation for 45 days; H_em = germinated seedlings after 32 °C incubation for 14 days).

GO enrichment analysis of the genes differentially expressed between each germination stage revealed more up-regulated genes with known functions than down-regulated ones. The gene expression processes and mechanisms in G_em/C_em and G_em/H_em exhibited significant similarities across all three levels. In the biological process category, metabolic processes displayed the highest gene expression, followed by single-organism metabolic processes. Within the molecular function category of G_em/C_em, catalytic activity had the highest gene expression, followed by oxidoreductase activity. However, in G_em/H_em, catalytic activity also had the highest gene expression, but it was followed by cation binding. Regarding the cellular component category, in G_em/C_em, the cell periphery exhibited the highest gene expression, whereas in G_em/H_em, the transcription factor complex displayed the highest gene expression. In contrast, when analyzing the biological process category of H_em/C_em, biosynthetic processes had the highest gene expression, followed by substance biosynthesis processes and cellular biosynthetic processes, respectively. In the molecular function category, structural molecule activity showed the highest gene expression, followed by cofactor binding. Within the cellular component category, cells had the highest gene expression (Figure 6).

**Figure 7:**
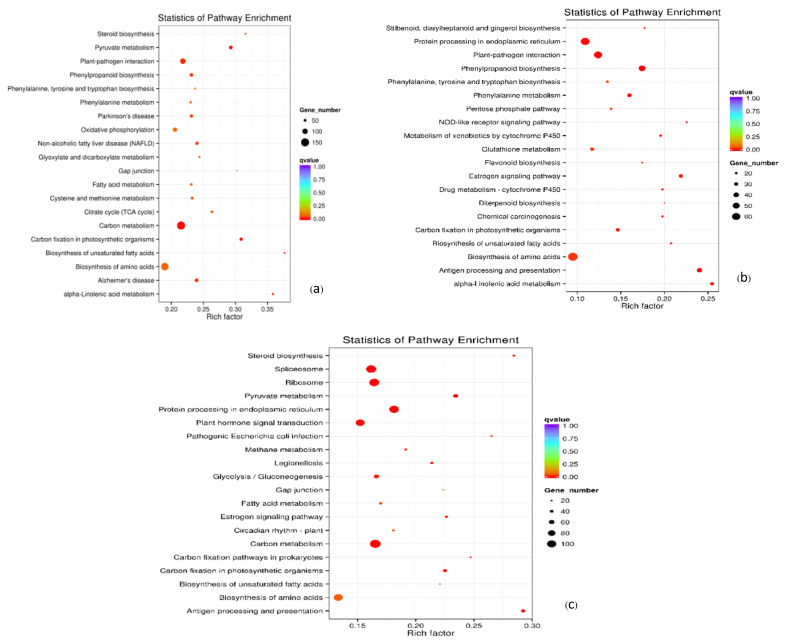
KEGG pathway enrichment analysis of the differentially expressed genes between oil palm seed germination stages. (**a**) DEGs between G_em and C_em. (**b**) DEGs between G_em and H_em. (**c**) DEGs between H_em and G_em. (C_em = seeds before incubation; G_em = seeds after 40 °C incubation for 45 days; H_em = germinated seedlings after 32 °C incubation for 14 days).

KEGG pathway enrichment analysis of the DEGs showed no significant differences between G_em/C_em and H_em/C_em. In both cases, the genes associated with the carbon metabolism pathway exhibited the highest expression levels, closely followed by those involved in the biosynthesis of amino acids pathway (in the case of G_em/C_em) and the ribosome pathway (in the case of H_em/C_em). Conversely, in the G_em//H_em comparison, genes related to the biosynthesis of amino acids pathway displayed the highest expression levels, with those involved in protein processing in the endoplasmic reticulum following closely behind (Figure 7).

### 2.5. Quantitative RT–PCR (qRT–PCR) Analysis of Specific Genes

**Figure 8:**
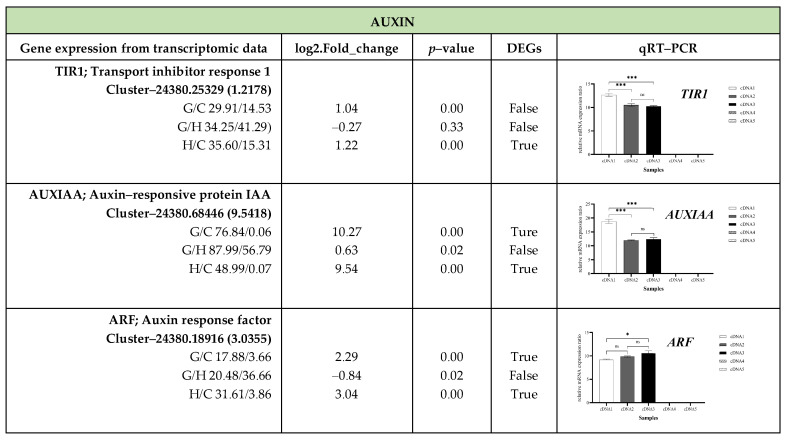

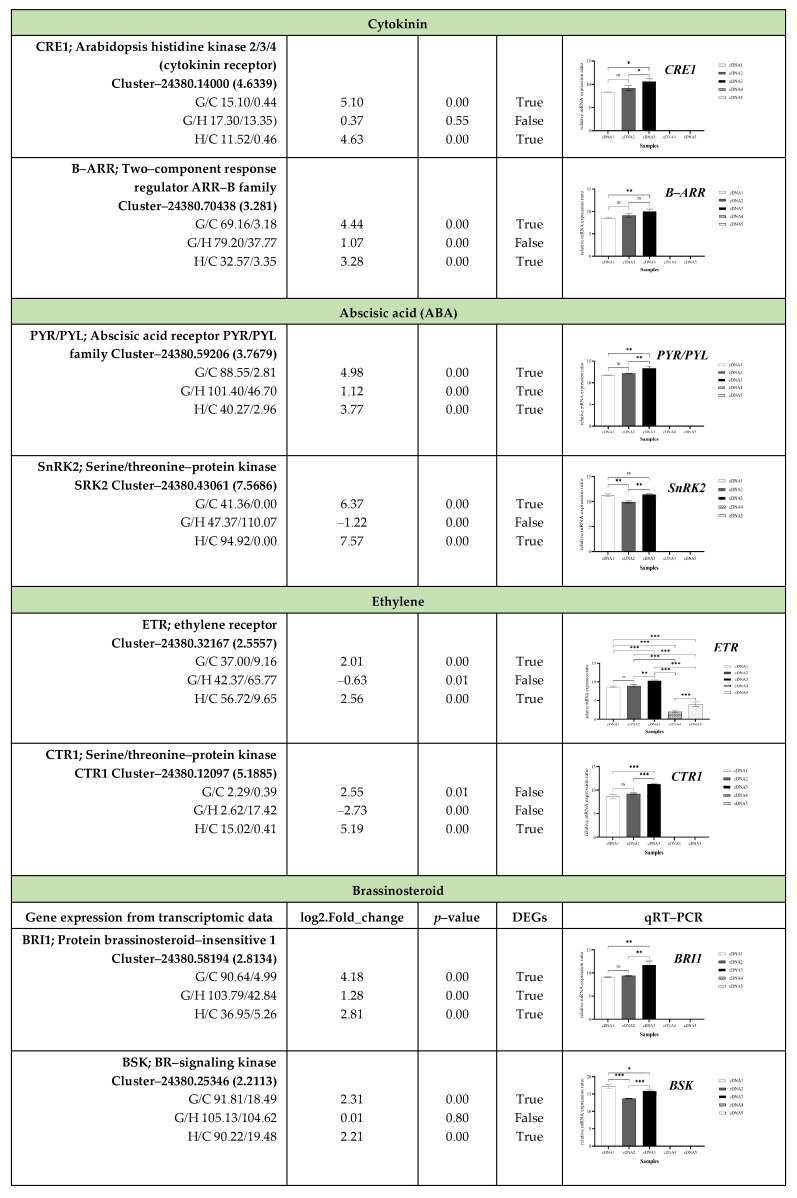

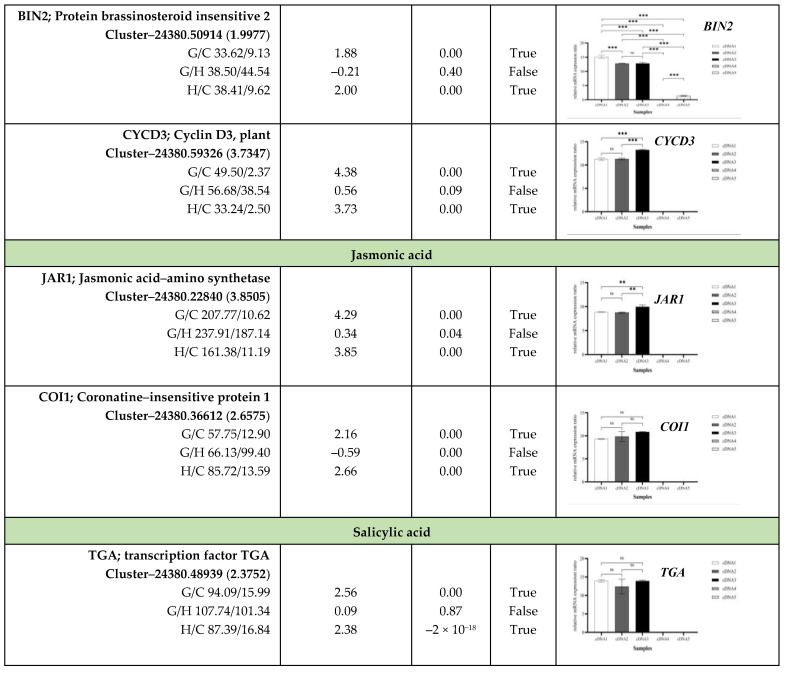
The expression of genes associated with plant hormone signal transduction validated via transcriptome analysis and qRT–PCR across four stages of oil palm seed germination. cDNA1 = seeds before incubation; cDNA2 = seeds after 40 °C incubation for 45 days; C. cDNA3 = germinated seedlings after 32 °C incubation for 14 days; cDNA4 = shoots of germinating seeds incubated at 32 °C for 60 days; cDNA5 = roots of germinating seeds incubated at 32 °C for 60 days. * = *p* < 0.05, statistical significance at the 5% level; ** = *p* < 0.01, statistical significance at the 1% level; *** = *p* < 0.001, statistical significance at the 0.1% level; ns = Not significant (*p* ≥ 0.05); NA = Not Available.

**Figure 9:**
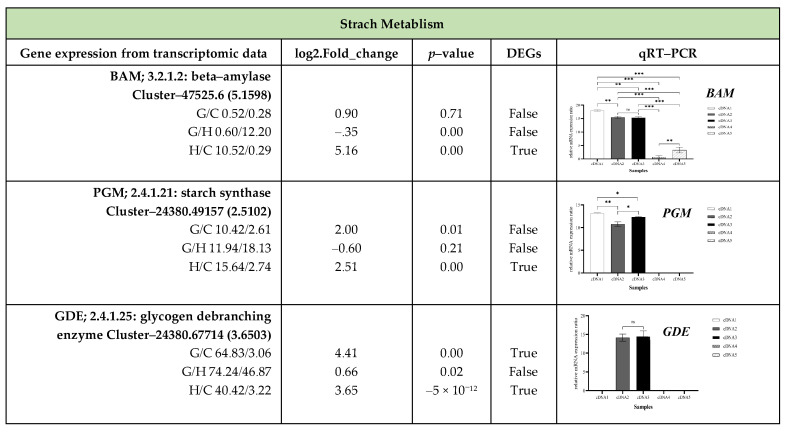

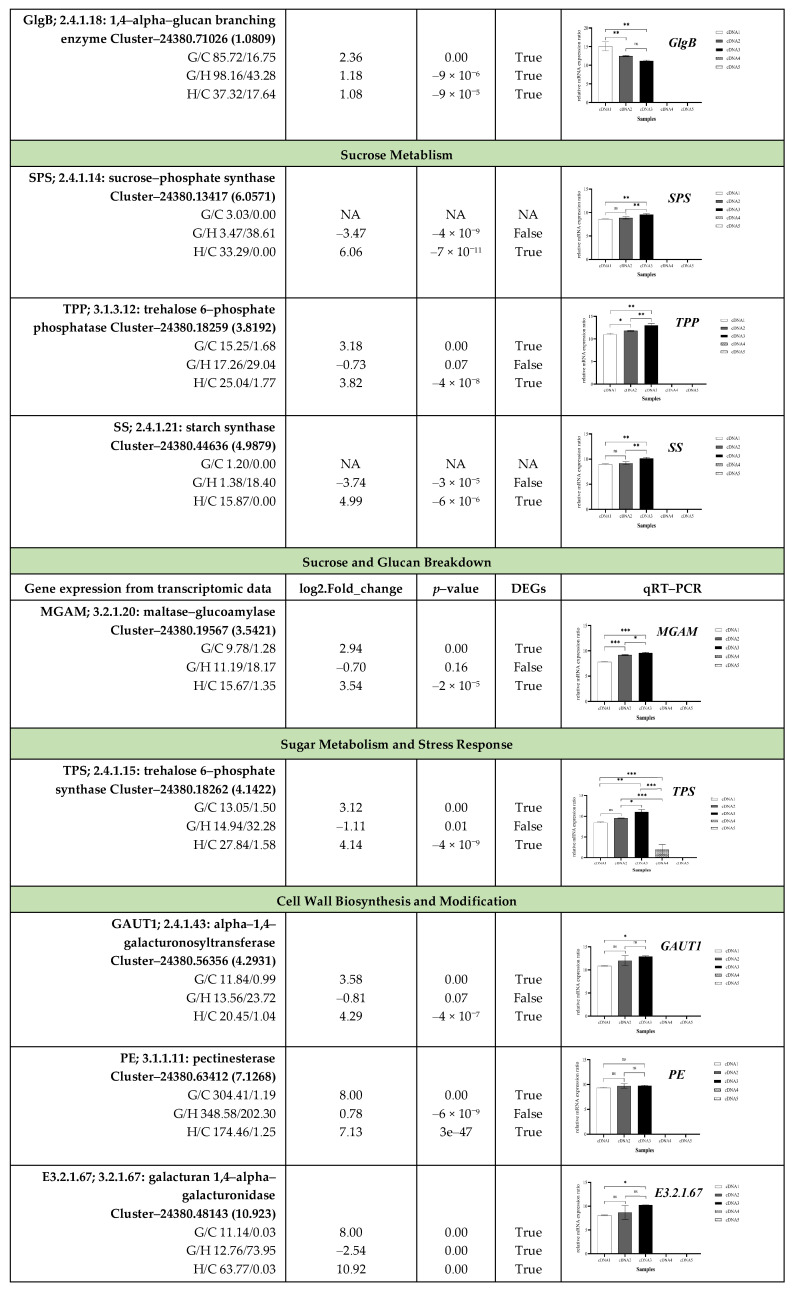

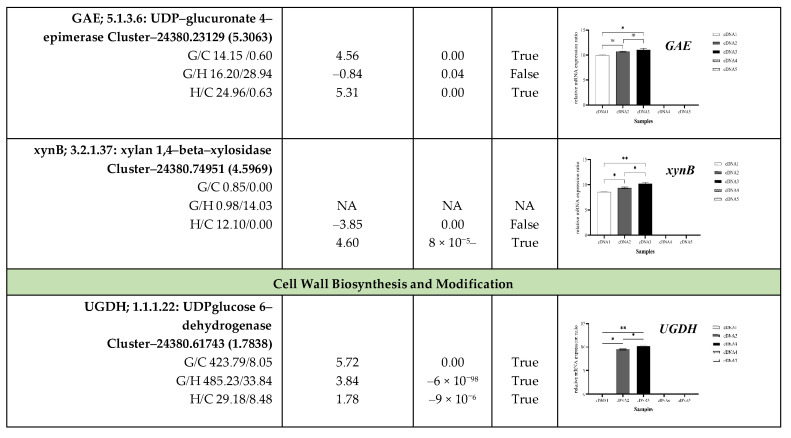
The expression of genes associated with starch and sucrose metabolism validated via transcriptome analysis and qRT–PCR across four stages of oil palm seed germination. cDNA1 = seeds before incubation; cDNA2 = seeds after 40 °C incubation for 45 days; C. cDNA3 = germinated seedlings after 32 °C incubation for 14 days; cDNA4 = shoots of germinating seeds incubated at 32 °C for 60 days and cDNA5 = roots of germinating seeds incubated at 32 °C for 60 days. * = *p* < 0.05, statistical significance at the 5% level; ** = *p* < 0.01, statistical significance at the 1% level; *** = *p* < 0.001, statistical significance at the 0.1% level; ns = Not significant (*p* ≥ 0.05); NA = Not Available.

To validate the transcriptome analysis across three stages of oil palm germination, up-regulated DEGs from 21 genes related to plant hormone signaling and 22 genes involved in starch and sucrose metabolism were examined. The results from qRT–PCR revealed that 14 out of 43 genes could not be amplified. These include seven genes involved in plant hormone signaling (*AUX1*, *AHP1*, *A*-*ARR, PP2C*, *ABF*, *BZR1*/*2*, *MYC2*) and seven genes related to starch and sucrose metabolism (*GlgC*, *GlgP*, *SUS*, *FRK*, *UXS*, *GPI, BGL*). The qRT–PCR analysis of oil palm seeds from the Chumphon Dura × Chumphon Pisifera variety and the transcriptome analysis of DEGs in the Nong Ped (D × P) variety revealed differences in gene expression patterns for particular genes (Figure 8 and Figure 9 and Supplementary Table S2).

qRT–PCR results revealed that during oil palm seed germination, several genes from different pathways showed their highest expression levels. In the pre-incubation phase (seeds before incubation), *T1R1* and *AUX*/*IAA* (auxin pathway), *SnRK2* (abscisic acid), *BSK* and *BIN2* (brassinosteroid pathway), and *BAM*, *PGM*, and *GlgB* (starch metabolism) had elevated expression. After heat treatment, *GAE* (cell wall biosynthesis and modification) and *GPI* (glycolysis and energy metabolism) were highly expressed, while no genes from the plant hormone signaling pathways were active. Following a 14–day incubation at 32 °C, most genes—particularly those in starch and sucrose metabolism—reached their peak expression. In the auxin pathway, *ARF* showed maximum expression, while *CRE1* and *B*-*ARR* were highly expressed in the cytokinin pathway. In the ethylene pathway, *ETR* and *CTR1* peaked, while *BRI1* and *CYCD3* reached maximum levels in the brassinosteroid pathway. Similarly, *JAR1* and *COI1* were highly expressed in the jasmonic acid pathway, and *PYR*/*PYL* in the abscisic acid pathway. Genes involved in sucrose metabolism (*SPS*, *TPP*, *SS*), cell wall biosynthesis and modification (*GAUT1*, *PE*, *E3.2.1.67*, *GAE*, *xynB*, *UGDH*, *MGAM*), starch and glucan breakdown, and sugar metabolism and stress response (TPS) exhibited peak expression. *TGA* (salicylic acid pathway) and *PE* (cell wall biosynthesis) showed no significant differences across stages. *SnRK2* (brassinosteroid pathway) was expressed in both pre-incubation and germinated seedlings (a 14-day incubation at 32 °C). *GDE* (starch metabolism) was highly expressed after heat treatment and in germinated seedlings. Additionally, *ETR*, *BIN2*, and *BAM* were more highly expressed in the root than the shoot of germinated seedlings incubated at 32 °C for 60 days, while *TPS* was exclusively expressed in the shoot.

In parallel, the transcriptome analysis of DEGs related to plant hormone signaling also revealed significant upregulation across several pathways during oil palm seed germination. *AUX*/*IAA*, *TIR1*, *ARF*, *CRE1*, *B*-*ARR*, *PYR*/*PYL*, *SnRK2*, *JAR1*, and *COI1* were strongly upregulated during germination. Additionally, *BRI1*, *BSK*, *BIN2*, *CYCD3*, *TGA*, *ETR*, and *CTR1* were upregulated in both germinated seedlings and after heat treatment. (Figure 8, Supplementary Table S2 and Figure S4). In sucrose metabolism identified significant upregulation in several genes, including in *GAUT1*, *PE*, *E3.2.1.67*, *GAE*, *xynB*, *UGDH*, *SPS*, *TPP*, *MGAM*, *GDE*, and *GlgB*, with particularly strong increases observed during germination. The highest expression levels were detected in germinated seedlings after 32 °C incubation for 14 days, followed by those incubated at 40 °C for 45 days, and finally in seeds before incubation. Notably, *SS* and *TPS* were highly upregulated in seeds incubated at 40 °C for 45 days, but their expression significantly decreased in germinated seedlings after 32 °C incubation for 14 days. *PGM* exhibited moderate upregulation during incubation at 40 °C for 45 days, with slight increases in germinated seedlings after 32 °C incubation for 14 days. *GPI* showed significant upregulation in incubated seeds but experienced minimal change followed by a significant decrease in germinated seedlings after 32 °C incubation for 14 days. *BAM* was significantly upregulated in seeds incubated at 40 °C for 45 days, with minimal upregulation in germinated seedlings after 32 °C incubation for 14 days and a subsequent decrease. Moreover, no genes were active in seed before incubation.

## 3. Discussion

Seed dormancy keeps seeds inactive even under favorable conditions, ensuring optimal germination timing. It can be influenced by factors such as hard seed coats, hormonal imbalances (ABA/GA), or underdeveloped embryos. Dormancy is typically broken through hormonal treatments, temperature shifts, light exposure, or physical stratification [1]. Therefore, transcriptomic analysis was conducted to understand gene expression during each germination stage of oil palm seeds.

The results from our transcriptome analysis of oil palm seeds at three developmental stages displayed meticulous data quality control. Each library exhibited an error rate consistently below 0.01%, with Q30 values surpassing 94% and a CG content falling within a range from 49.29% to 51.43%. After filtering, adapter removal, and eliminating low-quality reads, each library produced 38–47 million clean reads. This aligns with the findings of transcriptome analysis for *Phelipanche aegyptiaca* seed germination after activation with fluridone, TID108, and GR247, where the number of clean reads ranged from 21 to 25 million, with a Q30 value exceeding 94.90% and a CG content between 45.76% and 48.91% [25]. When de novo assembling the clean reads, a total of 148,695 unigenes with an average length (N50) of 1862 base pairs were identified. The majority of these unigenes fell within the 500–1000 base pair range, similar to the transcriptome data assembly for *P*. *lactiflora* seeds at various developmental stages using Illumina sequencing, which reported 24,688 unigenes [18]. Our analysis of oil palm seed data highlights the remarkable abundance of unigenes in comparison with similar experiments in other plant species [18,19,20,21,22].

In our study, GO function classification of 148,695 unigenes highlighted significant involvement in cellular, metabolic, and single-organism processes. These findings are consistent with studies on *P*. *lactiflora* [18] and *Polygonatum cyrtonema* [26], which emphasized the critical roles of cellular and metabolic processes in seed germination. The KOG function classification and KEGG analysis of oil palm seed germination stages in our study revealed a pattern similar to *P*. *lactiflora* seeds, with the highest expression in the translation pathway, followed by carbohydrate metabolism and folding, sorting, and degradation [27]. In contrast, *P*. *aegyptiaca* seeds showed increased activity in metabolism and biosynthesis pathways upon germination stimulation with fluridone TIS108 and GR24 [25].

To perform the functional analysis of DEGs between each seed germination stage, RNA-seq mapping was conducted, showing that 74–75% of reads successfully mapped to the reference genome. The G_em/C_em comparison revealed a high number of DEGs, indicating significant biological differences between the two conditions [28]. Upregulated genes in G_em/C_em may play a crucial role in defining G_em’s unique characteristics during germination by being involved in specific metabolic pathways or regulatory networks [29]. Shared DEGs suggest core regulatory pathways, while unique DEGs in G_em/C_em could be biomarkers. Gene cluster analysis identified three gene groups with distinct expression patterns, with H_em being more similar to G_em than C_em.

GO enrichment analysis of DEGs during seed germination revealed that upregulated genes were more associated with known functions than downregulated ones, suggesting that specific processes are activated to support germination. Our results differed from the findings in *P*. *lactiflora* seed germination regarding the biological process, molecular function, and cellular component categories [18]. However, both studies show significant enrichment in developmental processes at the germination stages compared to the initiation stage across different functional modules. This suggests that GO analysis of DEGs varies across plant types and dormancy-breaking methods.

KEGG analysis of DEGs during seed germination revealed that the carbon metabolism pathway had the highest expression levels in C_em/H_em and C_em/G_em, highlighting its role in energy and carbon provision. The ribosome pathway was most expressed in C_em/H_em, indicating high protein synthesis demand, while the biosynthesis of amino acids pathway was the second most expressed in C_em/G_em, being crucial for growth. In H_em vs. G_em, the biosynthesis of amino acids pathway was highest expressed, with significant expression of protein processing genes, emphasizing the importance of protein folding and transport. Our obtained results were similar to the KEGG analysis of DEGs of rice seed germination and young seedling growth stages [22], but they differed from the results for *P*. *lactiflora* seed development [18].

Analyzing gene expression patterns to uncover relationships among differentially expressed genes revealed some discrepancies between the qRT–PCR results and transcriptome analysis. While qRT–PCR, known for its precision, confirmed the broader trends identified in the transcriptome data, including the significant upregulation of key pathways [29], transcriptomics provides a comprehensive, genome-wide perspective [30]. It is important to note that the two methods were applied to different varieties of oil palm seeds. Despite these differences, both methods revealed the activation of plant hormone signaling pathways, along with starch and sucrose metabolism, working together during the dormant stage, heat treatment at 40 °C, and germination at 32 °C.

The gene expression profiles from both methods offer valuable insights into oil palm seed germination. Prior to incubation, the elevated expression levels of *TIR1*, *AUX*/*IAA*, *BSK*, *BIN2*, *SnRK2*, and *PE* detected via qRT–PCR suggest an early activation of *ARFs* and *BRI1*, similar to the patterns observed in soybean. In soybean, *BRI1* antagonizes ABA to promote germination, a role paralleled by *BSK* and *BIN2* in oil palm. Both species exhibit early activation of *SnRK2*, indicating the involvement of ABA, which is later suppressed to encourage growth. Additionally, the increased expression of PE in oil palm reflects the glycolytic activation observed in soybean, which is critical for energy production during germination. The coordination of ABA, GA, and BR signaling pathways plays a crucial role in both species, with BR and GA working in tandem to overcome ABA—mediated inhibition and drive germination [17]. Furthermore, the elevated ABA expression observed during the early stages of oil palm germination aligns with findings in wheat, where ABA biosynthetic genes showed a slight upregulation within the first 12 h after imbibition, as dormancy was broken, followed by significant downregulation during the later stages of germination [15].

After 45 days of incubation at 40 °C, qRT–PCR data indicated increased expression of *GAE* and *GPI*, pointing to active metabolic processes and structural changes essential for germination. This aligns with the transcriptomic data showing upregulation of genes such as *BRI1*, *BSK*, *BIN2*, and *CYCD3*, which promote cell elongation and division, vital for embryo growth. Additionally, the increased expression of *ETR* and *CTR1*, involved in ethylene signaling, suggests that stress responses and growth regulation are activated during germination [31]. The upregulation of SS and TPS supports the notion that seeds are mobilizing stored starch into sugars, providing energy for early development [32,33]. The consistent increase in GPI across both qRT–PCR and transcriptomic data highlights the significant energy demands necessary during germination. Previously, several studies have shown that heat treatment, especially at around 40 °C, has a significant impact on oil palm seed germination. A 50-day treatment at 37–39 °C was found to be optimal [34], while a 60-day treatment at 40 °C achieved a 56.3% germination rate, outperforming the 80-day standard [7]. Though effective in breaking dormancy, germination rates varied by progeny, likely due to genetic differences [7,8]. These results contrast earlier reports favoring an 80-day treatment [2,35], highlighting the importance of optimizing heat treatment duration for successful germination. Moreover, a 37-day heat treatment or HC treatment resulted in over 90% germination, compared to under 4% in untreated seeds, mainly due to reduced ABA levels, with HC also increasing IAA during imbibition. Gibberellins and cytokinins showed no direct role in dormancy release, though hormone sensitivity may be involved [6]. The findings above provide evidence that the duration of exposure to heat treatments, the application of chemical agents, and the genetic variations of progeny all play a critical role in breaking dormancy and promoting germination in oil palm seeds. Shorter or longer durations can either inhibit or enhance germination, depending on the precise timing. 

Molecular findings correspond with the structural changes observed in oil palm seeds after heat treatment. In dormant seeds, the thick cell walls in the micropylar endosperm acted as a barrier, blocking germination. However, after 60–70 days of heat treatment, this barrier weakened, allowing the embryo to exert pressure, leading to cracks in the endosperm and dislodging the operculum, which initially prevented water absorption. The heat treatment thus facilitated water entry and radicle emergence by weakening these physical barriers. SEM analysis further confirmed that untreated seeds had a lignified operculum, while heat-treated seeds exhibited cracks, enabling successful germination [4]. However, prolonged heat exposure was found to reduce germination by raising endocarp temperatures and decreasing enzyme activity [36]. This indicates that while heat treatment is essential for breaking dormancy and initiating germination, excessive heat can negatively impact seed viability. The upregulation of genes related to cell growth, energy metabolism, and stress response supports the structural changes observed after heat treatment. Our findings on molecular and previous physical modifications highlight that both methods are crucial for overcoming dormancy and promoting germination. This will lead to a better understanding of how to apply this knowledge to manage the germination of oil palm seeds across different varieties and production batches. In this study, a low germination rate was observed in the oil palm variety, which underwent a 45-day incubation at 40 °C to break dormancy. This duration may not have been sufficient to promote high and uniform germination. Based on these findings and previous studies that used longer heat treatment periods, further research will be conducted. This will include exploring the use of hormones or a combination of both methods to optimize the germination process of our oil palm seeds for commercial production

While our study used high temperatures (40 °C) for 45 days to break dormancy, in *P*. *sibiricum*, dormancy was managed through alternating warm stratification (25 °C) followed by cold stratification (4 °C). In warm temperatures, *P*. *sibiricum* initiate corm growth, while cold treatment reduces ABA levels and enhances GA signaling, breaking epicotyl dormancy and promoting seedling emergence. Despite these differences, both oil palm and *P*. *sibiricum* activate key pathways—glycolysis (*GAE*, *GPI*), BR signaling (*BRI1*, *BIN2*), and ethylene signaling (*ETR*, *CTR1*)—suggesting a conserved mechanism involving energy production, hormonal regulation, and cell cycle control that drives germination and seedling establishment [18]. In *P*. *cyrtonema*, after a 6-month dormancy period, the expression of starch metabolism genes (*α*-*Amylase*, *TPS*, *MGAM, β*-*Glucosidase*, and *EGLC*) was upregulated, mobilizing starch and sugars to fuel germination. Hormone genes related to auxin (*AUX1*, *TIR1*) and gibberellin (*GID2*, *PIF3*/*PIF4*) were also upregulated, promoting growth, while ABA-related genes (*PP2C*, *SnRK2*, *ABF*) were downregulated to break dormancy. Genes in phenylpropanoid and flavonoid biosynthesis pathways were upregulated, boosting flavonoid production and reducing lignin synthesis, facilitating seed coat breakdown and dormancy release [26]. The differences in gene expression and dormancy-breaking methods across species show that dormancy mechanisms are highly variable depending on the plant and environmental conditions.

Moreover, after incubation at 32 °C for 15 days, the germinated seeds exhibited the highest expression levels of several key genes across various pathways, as confirmed by both qRT–PCR and transcriptomic data. The gene expression patterns obtained from these two methods showed a high degree of similarity, with only a few genes displaying difference. This high level of gene expression similarity across methods underscores the coordinated activation of multiple hormonal and metabolic pathways, which are crucial for successful germination. The high expression of *AUX*/*IAA*, *TIR1*, and *ARF* in our transcriptomic data indicates their pivotal role in regulating cell division and elongation, processes essential for radicle emergence and early seedling growth [37]. Similarly, in *P*. *sibiricum*, 60 auxin-related genes were differentially expressed during seed dormancy and germination, highlighting auxin’s role in regulating these stages. Auxin signaling pathways in *P*. *sibiricum* were activated alongside other hormonal signals, such as ABA, GA, and BR, all of which are crucial for promoting seed germination and early growth [20]. This comparison underscores the conserved role of auxin and its interaction with other hormones in regulating germination across different plant species. The hormone signaling, particularly through *GAI1* and *ARF* genes, regulates GA and auxin (IAA) pathways, crucial for cell elongation, division, and dormancy release in *P*. *lactiflora*. These genes are differentially expressed across developmental stages, highlighting their role in transitioning from dormancy to active growth. *BRI1* also plays a key role in cell elongation during seed germination [18]. This coordinated hormone regulation ensures seeds can break dormancy and begin germination under favorable condition. 

Similarly, the high expression of *CRE1* and *B*-*ARR* in germinated seeds suggests their role in promoting cell division and differentiation during germination [38]. *BRI1*, *BSK*, *BIN2*, and *CYCD3* underscore the importance of brassinosteroids in cell elongation and growth during later stages of germination [39]. The increased expression of *ETR*, *CTR1*, *JAR1* and *COI1* indicates their role in stress response and growth regulation, helping seeds adapt to environmental conditions [40,41]. Additionally, *PYR*/*PYL* and *SnRK2* were selectively activated to prevent premature germination, ensuring favorable conditions for seedling establishment [42,43]. Our findings are aligned with *C*. *oleifera*, where similar hormone signaling genes—*CRE1*, *B*-*ARR*, *BRI1*, *BSK*, *BIN2*, *CYCD3*, *ETR*, *CTR1*, *JAR1*, and *COI1*-were expressed. These genes regulate cell elongation, division, stress responses, and dormancy, reflecting conserved mechanisms across both species [24]. In Callery pear, genes related to ABA, GA, auxin, ethylene, BR, and JA play crucial roles in seed dormancy and germination. The downregulation of *CYP707A* and *PP2C* during cold stratification lowers ABA levels, aiding dormancy release. Increased expression of *GA2ox* and DELLA proteins further supports dormancy release by counteracting ABA. Additionally, the differential expression of *BRI1* and *TIR1* regulates cell elongation and division, facilitating the germination process [19]. Moreover, submergence tolerance in rice seeds (*Oryza sativa*) involves genes like *ERF*, *CTR1*, and *TPS*, which regulate energy metabolism and stress tolerance. These genes maintain energy balance, enabling seed survival and germination under low-oxygen conditions [44].

Additionally, the upregulation of *SPS*, *TPP*, *SS*, and *TPS* in our study suggests active energy mobilization through starch conversion to sugars, supporting early growth, similar to findings in *C*. *oleifera*. The increased expression of *GPI* highlights the energy demand during dormancy breaking and rapid growth, aligning with the glycolysis-related activity seen in *C*. *Oleifera* [24]. The upregulation of *GAUT1*, *GAE*, *UXS*, and *UGDH* emphasizes the importance of cell wall biosynthesis and modification, essential for seedling development in both species. Moreover, as reported in macaw palm (*Acrocomia aculeata*)**,** the degradation of cell wall carbohydrates, particularly (galacto)mannans, plays a key role in endosperm thinning and cotyledonary petiole emergence [45]. This supports the critical role of cell wall remodeling in germination, as also highlighted in our study, where genes involved in cell wall biosynthesis are actively upregulated. These findings align with the oil palm germination process discussed in [46], where carbohydrate remobilization and cell wall modification are necessary to facilitate seedling emergence and growth. The interplay between energy mobilization and structural changes is critical for overcoming dormancy and ensuring successful seed germination. Furthermore, genes related to sucrose and starch metabolism, such as SPS, SS, and hexokinase, are upregulated during dormancy release, highlighting the critical role of energy mobilization in supporting seedling growth, as observed in Callery pear [19].

Furthermore, during the germination stage, GA-related genes in oil palm seeds consistently showed low upregulation, supporting the finding that GA is not a critical factor in the natural germination process of some oil palm varieties [46]. Hormonal profiling of germinating oil palm seeds has shown that several hormones fluctuate during the initial days, with GA exhibiting only a slight increase [6]. The antagonistic relationship between ABA and GA is expected, as it is a key component of dormancy breaking in seeds with mixed physical-physiological dormancy [47,48]. Similar to Arabidopsis germination, the interaction between GA and ABA involves a complex network [42] and includes the COP9 signalosome, which regulates protein degradation via the proteasome [49]. In contrast, wheat seed germination shows significant upregulation of GA, BA, and ethylene biosynthetic genes throughout the process, consistent across both eudicot and monocot seeds [15]. Similarly, in soybean, GA-related genes such as *GID1* and *SLY1* are highly expressed early in germination. In soybeans, GA plays a key role in promoting germination by degrading DELLA proteins, releasing the growth-promoting effects of GA and counteracting ABA inhibition [17]. Moreover, in *P*. *sibiricum seeds*, GA biosynthesis genes show stage-specific expression during dormancy release, with *GA3ox* upregulated in early stages and *GA2ox* during later stages. GA signaling genes (*GID1*, *GASA*, *SPY*) are highly expressed during cold stratification and seedling emergence, indicating GA’s key role in promoting germination. In contrast, oil palm relies more on other hormonal pathways, as GA expression is lower. These species-specific differences highlight distinct strategies for dormancy release, with GA being more central in *P*. *sibiricum*, while oil palm may rely more on auxin or BR signals [20].

**Figure 10:**
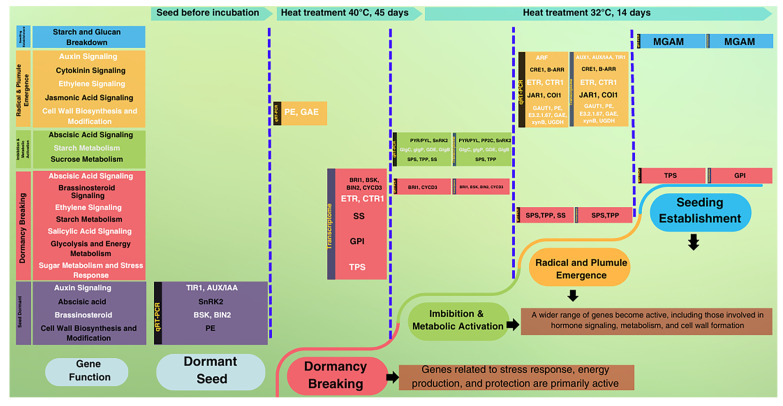
Summary of upregulated genes related to plant hormone signal transduction and starch/sucrose metabolism via qRT–PCR and transcriptomic data during oil palm seed germination across three stages: seeds before incubation, seeds incubated at 40 °C for 45 days, and germinated seedlings incubated at 32 °C for 14 days.

Our findings reinforce the roles of hormones and genes in starch and sucrose metabolism during oil palm seed dormancy and germination, while also uncovering specific genetic mechanisms driving these processes (Figure 10). These insights may have broader implications for dormancy breaking and germination in other palm species, potentially enhancing seed germination and consistency for commercial production. 

## 4. Materials and Methods

### 4.1. Oil Palm Seed Preapration

Seeds from the oil palm variety “Nong Ped (D × P)” from Pao-Rong Oil Palm Co., Ltd., Nakhon Si Thammarat, Thailand, were subjected to dormancy breaking followed the company’s procedure. Fully mature oil palm bunches were harvested, incubated for 3–7 days, separated, and then blended to minimize the fibrous covering. Simultaneously, the seeds underwent a soaking period of 5–7 days in water before the outer fibers were scrubbed off. To prevent fungal growth, the seeds were immersed in 10% sodium hypochlorite (Worldchemical Company, Bangkok, Thailand) for 30 min, dried in the shade for 7 days, and stored at 18–20 °C before use. 

**Figure 11:**
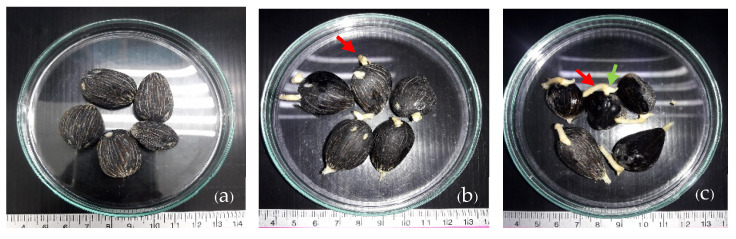
The stages of oil palm seed germination used in this study. (**a**) C_em = seeds before incubation. (**b**) G_em = seeds after 40 °C incubation for 45 days. (**c**) H_em = germinated seedlings after 32 °C incubation for 14 days (red arrows = roots; green arrow = shoot).

In this experiment, three stages of oil palm seed germination were utilized. The first stage was represented by the seeds after drying, referred to as the seeds before incubation (C_em). The second stage was represented by the embryos after incubation at 40 °C for 45 days, designated as H_em, and the third stage was represented by the germinating embryos incubated at 32 °C for 14 days, labeled as G_em (Figure 11). As part of the preparation process, twenty oil palm seeds were placed in a plastic bag, measuring 16 × 20 inches. The seeds were moistened by spraying them with water once a week, ensuring that the moisture content remained between 80 and 85%. The bags were then tightly sealed with a rubber band. Subsequently, the bagged seeds were transferred to an incubation room and were kept in the dark at a temperature of 40 ± 1 °C for 45 days to break their dormancy. After this incubation period, the seeds were removed from the bags and arranged in a plastic basket. The basket was covered with a 16– × 20–inch plastic bag, sprayed with water weekly to maintain the moisture level at between 80 and 85%, and tightly sealed with a rubber band. The basket was then kept in the dark at 32 °C for at least 14 days to facilitate germination. Using this method, a germination rate of 80–90% for the seedlings was achieved.

All oil palm embryos at the three different stages mentioned above were utilized for RNA extraction using the TriPure^TM^ Isolation Reagent (Merck, Darmstadt, Germany) procedure. Three replicates of each sample were quality-checked with a NanoDrop spectrophotometer and 1.0% agarose gel electrophoresis. The highest-quality samples from each set were selected and sent for sequencing via Illumina NovaSeq 6000 at NovogeneAIT Genomics Singapore Pte. Ltd. (Singapore).

### 4.2. Transcriptome Analysis, De Novo Assembly and Annotation

In brief, libraries were prepared with the NEBNext^®^ Ultra™ RNA Kit (Illumina^®^, San Diego, CA, USA). mRNA was purified, fragmented, and converted to cDNA, and adapters were added. cDNA fragments were purified and checked with the Agilent Bioanalyzer 2100. Samples were clustered and sequenced on an Illumina platform. Quality control involved processing raw fastq data with in-house perl scripts to remove adapters, poly-N, and low-quality reads. Clean data quality was assessed by calculating the Q20, Q30, GC content, and sequence duplication levels. 

Transcriptome assembly was performed using the Trinity method [50], and gene function was annotated based on several databases, namely the NCBI non-redundant protein sequences (Nr), NCBI non-redundant nucleotide sequences (Nt), Protein family (Pfam) [51], Clusters of Orthologous Groups of proteins (KOG/COG), Swiss-Prot (UniProtKB/Swiss-Prot), KEGG Ortholog (KO) [52], and Gene Ontology (GO) databases. Picard-tools (v1.41) and samtools (v0.1.18) were utilized for sorting and removing duplicates in alignment results, while GATK3 [53] was employed for single nucleotide polymorphism (SNP) identification. Simple sequence repeats (SSRs) were identified using MISA (http://pgrc.ipkgatersleben.de/misa/misa.html, accessed on 15 March 2022), and primers were designed using Primer3 (http://primer3.sourceforge.net/ releases.php, accessed on 20 March 2022).

Gene expression levels were estimated using RNA–Seq via expectation maximization (RSEM software, v1.1.17) [54], and differential expression analysis was conducted using DESeq (1.10.1) R packages [55]. The resulting *p*-values were adjusted using the Benjamani-Hochberg approach [56] for controlling the false discovery rate. Genes with an adjusted *p*-value < 0.05 found via DESeq were assigned as differentially expressed. For samples without biological replicates, read counts from each sequenced library were normalized using edgeR [57]. Differential expression analysis between two samples was performed using DEGseq [55] with *p*-values adjusted using q-values. Significantly differentially expressed genes were identified using a q-value threshold of <0.005 and |log2(fold change)| > 1. 

GO enrichment analysis of the differentially expressed genes (DEGs) was performed using GOseq R packages [58], and KEGG pathway enrichment was assessed using KOBAS [59].

### 4.3. –qRT–PCR Analysis

To gain a comprehensive understanding of oil palm seed germination, 43 upregulated genes associated with plant hormone signal transduction and starch and sucrose metabolism were selected from the transcriptome database. The expression of these genes was validated using two methods, namely qRT–PCR and log2 fold change, with the latter reflecting the difference in gene expression between two conditions on a logarithmic scale. This analysis was conducted across four stages of oil palm seed germination: the first three stages (cDNA1, cDNA2, and cDNA3), as mentioned earlier, and a fourth stage involving germinated seeds incubated at 32 °C for 60 days. Their shoots were labeled as cDNA4, while their roots were labeled as cDNA5. For qRT–PCR testing, the oil palm hybrid Chumphon Dura × Chumphon Pisifera was used, while the oil palm hybrid Nong Ped (D × P) was analyzed for differentially expressed genes (DEGs) using log2 fold change in our transcriptome study. 

The primers were designed to be 18–20 bp in length, with a PCR product size of 200–300 bp, a CG content of 40–60%, and a melting temperature of 50–70 °C, adjusted for salt with both forward (F) and reverse (R) primers. The primer design was performed using the Primer3 Input tool (http://bioinfo.ut.ee/primer30.4.0/, accessed on 21 January 2022) (Supplementary Figure S4) and The Oligonucleotide Properties Calculator (http://www.basic.northwestern.edu/biotools/oligocalc.html, accessed on 21 January 2022) (Supplementary Table S3).

RNA of the four oil palm stages was extracted and quality—checked as mentioned above. The extracted RNA was reverse-transcribed into cDNA using the iScriptTM Select cDNA Synthesis Kit (Bio-Rad, Hercules, CA, USA). The 20 μL reaction mixture consisted of 2 μL of total RNA (2 μg), 1.0 μL of oligo (dT) primer (2.5 μmolar), 4.0 μL of reaction buffer, 0.5 μL of protector RNase inhibitor (40 unit/μL), 2.0 μL of deoxynucleotide mix (dNTP), 1.0 μL of reverse transcriptase, and 8.5 μL of sterile dH_2_O. The cDNA synthesis reaction was performed in an Eppendorf T100 Thermal Cycler (Bio-Red, Hercules, CA, USA) by incubating at 65 °C for 10 min, followed by quick chilling on ice until cool. The incubation was then continued at 45 °C for 30 min and 85 °C for 5 min, and terminated by heating at 95 °C for 5 min. All cDNA samples were stored at −80 °C until further use.

For qualitative examination, the obtained cDNA was amplified using 18S rRNA under the following conditions: an initial denaturation at 95 °C for 4 min, followed by 35 cycles of 95 °C for 30 s, 55 °C for 30 s, and 75 °C for 1 min. A final extension was performed at 75 °C for 7 min, followed by holding at 4 °C. The PCR products were then run on a 1.5% agarose gel. 

For quantitative analysis, the expression levels were determined using the Applied Biosystems 7300 Real-Time PCR System (Applied Biosystems, Waltham, MA, USA). The 10 μL real-time PCR mixture included 1 μL of tenfold diluted cDNA, 0.25 μL (10 μM) of each primer, 2 μL of HOT FIREPol^®^ EvaGreen^®^ qPCR Mix Plus (ROX) (Solis BioDyne, Tartu, Estonia), and 6.5 μL of nuclease-free water. The PCR conditions were set as follows: an initial denaturation at 95 °C for 15 min, followed by 40 cycles of 95 °C for 30 s, 55–65 °C for 30 s, and 72 °C for 30 s. Each sample was analyzed in triplicate, and the expression levels were normalized to the 18S rRNA reference gene. The relative gene expression levels were calculated using the 2^−ΔΔCt^ method [60]. Data analysis was performed using IMS SPSS Version 22 (one-way ANOVA, nonparametric multiple-comparison test), with significance levels between different samples set at *p* ≤ 0.05. 

## 5. Conclusions

Our study underscores the complex genetic and molecular processes that regulate seed dormancy and germination in oil palm. Through transcriptome analysis, we identified critical stages, including ABA degradation, energy mobilization, starch breakdown, and cell elongation and division, that drive the transition from dormancy to active germination. Key regulatory genes across hormone signaling and metabolic pathways coordinate these processes, ensuring successful seedling development. The discovery of variety-specific gene expression differences opens up new opportunities for developing tailored approaches to dormancy management and germination enhancement. By leveraging these genetic insights, producers can adopt targeted strategies, such as customized incubation protocols and precise chemical treatments, to cater to the unique requirements of each variety. Such advancements will not only optimize germination consistency and speed but also increase the efficiency of large-scale oil palm seedling production, reducing costs and improving yield quality for commercial growers.

Additional information:

The datasets generated during the current study are available in Breaking dormancy in oil palm seed, accession no. CRA029979, (https://bigd.big.ac.cn/gsa/browse/CRA018610, accessed on 5 September 2024)

## Figures and Tables

**Table 1:** Data quality control.

Sample	Raw Reads	Clean Reads	Clean Bases	Error (%)	Q20 (%)	Q30 (%)	GC Content (%)
C_em	49,354,132	47,987,134	7.2G	0.01	97.84	94.37	51.43
H_em	39,702,154	38,351,078	5.8G	0.01	98.16	95.01	49.29
G_em	45,434,898	44,641,578	6.7G	0.01	98.05	94.81	49.73

C_em = seeds before incubation; G_em = seeds after 40 °C incubation for 45 days; H_em = germinated seeds after 32 °C incubation for 14 days.

**Table 2:** The ratio of unigenes annotated in all databases.

Annotated	Number of Unigenes	Percentage (%)
Annotated in NR	74,595	50.16
Annotated in NT	84,253	56.66
Annotated in KO	27,273	18.34
Annotated in Swiss-Prot	52,250	35.13
Annotated in PFAM	53,987	36.30
Annotated in GO	54,064	36.35
Annotated in KOG	23,333	15.69
Annotated in all Databases	13,779	9.26
Annotated in at least one Database	94,506	63.55
Total Unigenes	148,695	100

**Table 3:** Transcriptome data of oil palm seed development after being filtered via the RSEM program.

Sample Name	Clean Read Numbers	Total Mapped
C_em	47,987,134	36,003,414 (75.03%)
H_em	38,351,078	28,973,156 (75.55%)
G_em	44,641,578	33,173,008 (74.31%)

C_em = seeds before incubation; G_em = seeds after 40 °C incubation for 45 days; H_em = germinated seedlings after 32 °C incubation for 14 days.

## Data Availability

Data are contained within the article and Supplementary Materials.

## Supplementary Materials

The following supporting information can be downloaded at https://www.mdpi.com/article/10.3390/plants13192680/s1, Figure S1: Distribution of transcript length and unigenes; Figure S2: A Venn diagram illustrating the distribution proportion of unigenes across five out of the seven databases; Figure S3: The distribution of FPKM in each oil palm sample. C_em = seeds before incubation; G_em = seeds after 40 °C incubation for 45 days; H_em = germinated seedlings after 32 °C incubation for 14 days; Figure S4: Gene names, gene details, base sequence, and base selection for primer design; Table S1: Statistics of the assembly quality; Table S2: Gene expression of hormone signaling pathways and starch and sucrose metabolism determined via qRT–PCR; Table S3: Primer sequences for genes associated with plant hormone signal transduction and starch and sucrose metabolism.

## Abbreviations

ABA	abscisic acid
AUX1	auxin influx carrier protein 1
AUX/IAA	auxin/indole-3-acetic acid
ARF	auxin response factor
BAM	beta-amylase
B-ARR	type-B Arabidopsis response regulator
BIN2	brassinosteroid-insensitive 2
BGL	beta-glucosidase
BRI1	brassinosteroid insensitive 1
BSK	BRI1 suppressor kinase
BZR1/2	brassinazole resistant 1/2
cDNA	complementary deoxyribonucleic acid
C_em	seeds before incubation
COI1	coronatine-insensitive 1
CRE1	cytokinin response 1
CTR1	constitutive triple response 1
CYCD3	cyclin D3
DEGs	differentially expressed genes
E3.2.1.67	xylan 1,4-β-xylosidase
ETR	ethylene response 1
FPKM	fragments per kilobase of transcript sequence per million base pairs sequenced
FRK	fructokinase
GAE	galacturonosyltransferase-like 1
GAs	gibberellins
G_em	seeds after 40 °C incubation for 45 days
GO	Gene Ontology
GAUT1	galacturonosyltransferase 1
GDE	glycogen debranching enzyme
GlgB	glycogen branching enzyme
GlgC	glucose-1-phosphate adenylyltransferase
glgP	glycogen phosphorylase
GPI	glucose-6-phosphate isomerase
H_em	germinated seedlings after 32 °C incubation for 14 days
HC	hydrogen cyanamide
JAR1	jasmonic acid resistant 1
KEGG	Kyoto Encyclopedia of Genes and Genomes
KOG	eukaryotic orthologous groups
MGAM	maltase-glucoamylase
MYC2	myelocytomatosis 2
NCBI	National Center for Biotechnology Information
NGS	next-generation sequencing
NR	non-redundant protein
NT	nucleotide database
PE	pectinesterase
PFAM	Protein families database
PGM	phosphoglucomutase
PP2C	protein phosphatase 2C
PYR/PYL	pyrabactin resistance/pyrabactin resistance-like
qRT–PCR	quantitative reverse transcription polymerase chain reaction
RNA	ribonucleic acid
SEM	Scanning Electron Microscopy
SnRK2	SNF1-related protein kinase 2
SPS	sucrose-phosphate synthase
SS	starch synthase
SUS	sucrose synthase
TIR1	transport inhibitor response 1
TGA	TGA—binding factor
TPP	trehalose-6-phosphate phosphatase
TPS	trehalose-6-phosphate synthase
UGDH	UDP-glucose 6-dehydrogenase
UXS	UDP-glucuronic acid decarboxylase
xynB	xylanase B
