# Supplementary material for: Decoding the Transcriptomics of Oil Palm Seed Germination
Source: Plants (Basel). 2024 Sep 24;13(19):2680. doi: 10.3390/plants13192680 (PMC11479028; doi:10.3390/plants13192680)
Supplement: Supplementary file 1 [file plants-13-02680-s001.zip › plants-3185067-supplementary.pdf]

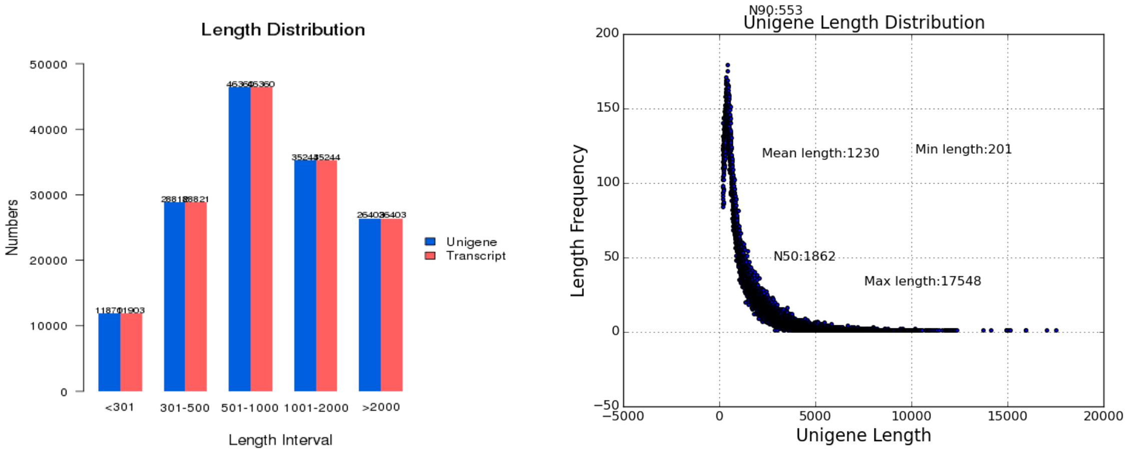

Supplementary Figure 1. Distribution of transcript length and unigenes.

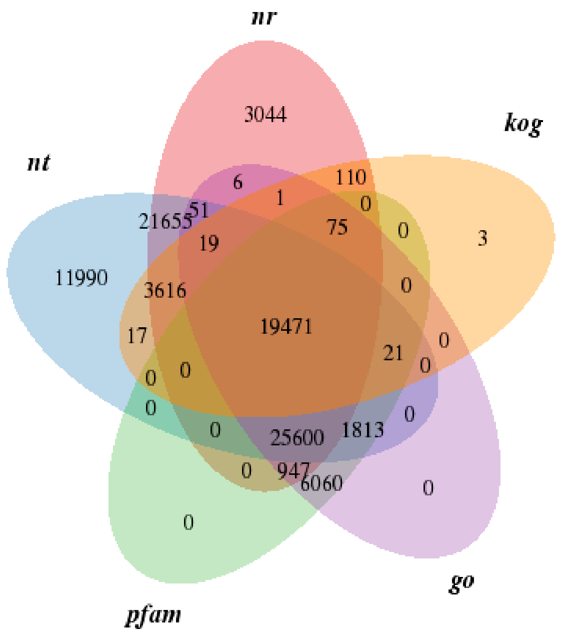

Supplementary Figure 2. A Venn diagram illustrating the distribution proportion of unigenes across 5 out of the 7 databases

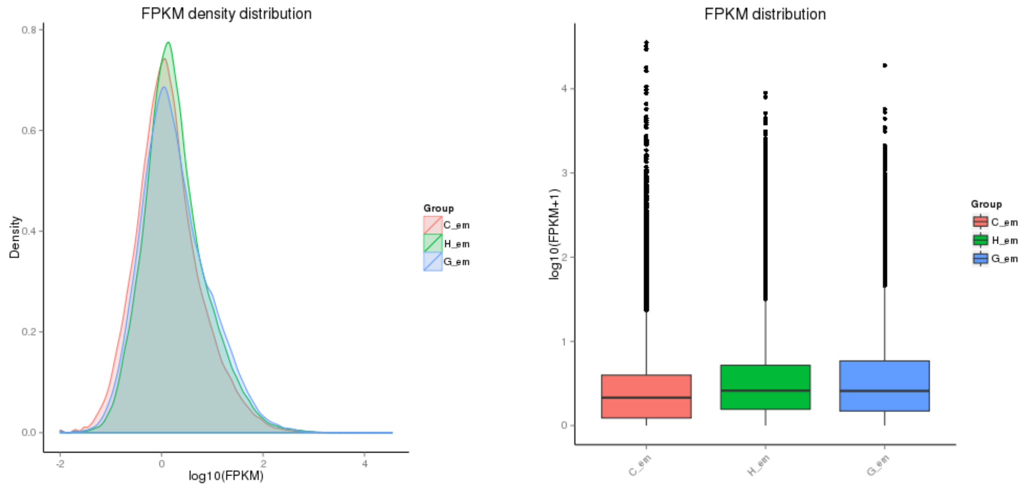

Supplementary Figure 3. The distribution of FPKM in each oil palm sample. C_em = seeds before incubation; G_em = seeds after 40°C incubation for 45 days; H_em = germinated seeds after 32°C incubation for 14 days

Supplementary Figure 4. Gene names, gene details, base sequence, and base selection for primer design.

GAUT1
2.4.1.43: alpha-1,4-galacturonosyltransferase
>Cluster-24380.56639;orf1 len=1473 frame:-2 start:1790 end:318 gi|743848988|ref|XP_010939506.1| PREDICTED: probable galacturonosyltransferase 9 [*Elaeis guineensis*]
GCTGAGGTAGGGGCGGAGGTAGGCGCCTGCGGCGGAGGGATCGCCGGCAACGGTGGAGTCC
GAGGAGGCGGAGGTGGCGGCGGAGGACGAGGGGTTGGCGGAAAGCAGCACCGAGAGGGCC
GCCAGGAAGAGGAGGGTGAGCATCGCCGACACGAACACCCGGTAGCTGAACACCACACGGA
TGCCCGTCGCCGGACGGCCAGGCCGGCCTCCCGCCAGCTCCGCGTCTTTGAGGGCCTCGCCG
CCTCCCTCTCCTCCCTCGCCGCCCGCCTCAATTCCGGCCTCCCCTCCGATGAGGACTCCCTCC
GCCCCCTCGAGAAGGAGGCCAAGGACCGGATCAAGCTCGCCCGCGCCCTCATCTCCGAATCC
AAGGAGTCCTTCGACACGCAGCTCAAGGTCCAGAAGCTCCGCGACACCATCTTCGCCGTCCA
CGAGCAGCTCCACCGCGCCAAGAAGCTCGGCGCCCTCACCAACCGCATCGCCGCTGGATCCA
CCCCCAAGAGCCTCCACTGCCTCGCCATGCGCCTCATGGAGGACCGCATCGCCCACCCGGAC
TCCTACCGCCGGCCCGGCCCCGAACCACCGGAGCTCGCCGACCCAGGCCTCTACCACTACGT
CCTCATCTCCGACAACGTCATCGCCGTCTCCACCGTCGTGAACTCCGCCATCCGGAACGCAGC
CGAGCCCTGGAAGCATGTCTTCCACATCGTGACCGACCCCATGTACCTCGACGCGATGCAGGT
CTGGTTCACCCTTCGCCCGCCCGCAAGCGGCGCCCGGGTGGAGGTCAAGTCGGCGGCGGACT
TCGGGTTCTTGAATTCCTCCTACTCGCCGGTGATGCGGCAGATTGAAGGCGCGCGGCGGGATT
TGGCGCTGCTGGACTACCTGAGGTTCTACTTGCCGGAGATGTACCCGAGGCTGCGGCGGGTG
GTGTTCTTGGAGGACGACGTGGTGGTGCAGAAGGATCTGGCGGGGCTGTGGAGGGTGGATTT
GGACGGGAAGGTGAACGGGGCGGTGGAGATGTGCTTTGGGGGCTTCAGGAGGTACGGTAGG
TACATGAACTTCTCCCATCCTGTGGTCCAGGAGCGGTTCAGCCCAAGGGCTTGCGCATGGGCC
TACGGGGTGAATGTCTTTGATCTCGACGCATGGCGGAGGGAGAGGTGCACTGAGCAATTCCA
CGAGTACCAGGATTTGAATGAAGATGGAATGCTCTGGAACCCAGGGACCGTGCTTCCAGCAG
GATTGATGACGTTTTACACAACCACTAAACCATTAGACAAATCATGGCATGTGATGGGACTTG
GTTACAATCCAAGTATTAGTCCAGAGAAAATCCTCAATGCTGCAGTCATACACTTCAATGGGA
ACATGAAGCCATGGCTCGATGTTGCCTTGAACCAATACAAGCATCTATGGACCAAGTATGTAG
ATACTGAAATGCAATTCCTCCCGCTCTGCAACTTTGGCCTCTAA
F: CCTCTACCACTACGTCCTCA
B: ACCTCAGGTAGTCCAGCAG

PE
3.1.1.11: pectinesterase
>Cluster-24380.63412;orf1 len=1797 frame:-2 start:2019 end:223 gi|743850281|ref|XP_010939846.1| PREDICTED: pectinesterase-like [*Elaeis guineensis*]
GATCTCCACTCTCCAACCGCAATGACAGCCCACCAGAATTCCGATTCCACTGCCTCCCAGTTT
GATAACTCAAAGCAGAGGAGAAAGCTCCTCCTCCTCCTCCTCACCTCCTTCCTCCTCCTCGCC
GCCGTCATCGCCACGGCCTCCACCCTCTTCGCCCGCCGCGCCGTCACCTCCCACGAGCCCACC
GTCTCCCATGCCATCCTCCGCTCCTCCTGCCGCTCCACCCGCTATCCCGACCTCTGTTACTCCG
CCGTCGCCGCCACCCCGGCACTGATCCAGACCATCTCAAAACCCAAGGACGTCATCCACGCC
TCCATCAACCTCACCATCACCGCCATCCAGCGCTCCTTCCGCCACGTCGAGCGGCTCTCCGCG
TCCTACCCCAACCTCACCACGCGGGAGCGTACTGCCCTCCATGACTGCCTCGAGATGTTCGAC
GAATCACTCGACGAGCTCAACCTCACCGACGCCGACCTCCGGGCATATCCCGCCGGCAAGCC
CCTCCGTCGGCATGCCCTCGACCTTGAGATTCTGGTCAGCGCCGCCATGACCAACCAGGAGTC
GTGCTCTGATGGCTTCTCCTACGAGGACATCGAGGGCCGCCTCCGCCGCGAGATCCGCTCCGA
GCTCACGCACGTGACCCGCATGTGCCGCAACGCCCTCGCCATGATCAAGAACATGATCGACG
CCGACATTGCTGCTGCGGAGGCGGAGGCGGGGGCGGACGGCGGGGCACTCAACCAGGGACG
GCGGATGTCGGGTTTCGACGTCGATGAGGAGGGTTTCCCGGATTGGATGGAGAAGAAGGATA
GGAGGCGGCTGCTGCAGGCCGCGGGGGAGGTGCAAGTGAATGCGGTGGTGGCAGCGGACGG

GAGCGGGGATTACAAGACAGTGGGGGAGGCGGTGGCTGCGGCGCCGGCAAAGAGCACGAC
GAGGTGGGTGATACGAATAAAGGCGGGGACGTATGAAGAGAATGTGGAGGTGCCCAAGAAG
AAGACGAATTTGATGTTCATCGGAGACGGGAGGACGGCCACCATCATCACCGGCAGCCGGAA
CGTCATGGACGGGAGCACCACCTTCCATTCCGCCACCTTAGCCGTTGTAGGGACAGGGTTCCA
AGCCCGCGGCCTCCGTATCGAGAACACGGCCGGCCCGTCGAAGCACCAGGCGGTGGCCCTCC
GCGTGAGCTCCGACCTCTCGGCCTTCTACGACTGTGACCTCATCGGCTACCAGGACACCCTCT
ACCTCCACTCCCTCCGCCACTTCTTCCGCAGCTGCCTCATCCAGGGCACCGTCGACTTCATCT
TCGGCAACGCCGCCACCGTCCTTCAGGACTGCGACATCCAGCTCCGCCGCCCCAACCCCAAC
CAAAAGAACATGGTCACCGCCGATGGCCGCGACGACCCCAACGAGCCCACCGGCATCACCAT
CCACCGCTCCCGCATCGCCGCCGCCCCCGACCTCGAGCCCGTTAAGAGCTCGTTTCGGTCGTA
CCTCGGGAGGCCATGGAAGGAACACTCAAGGACGGTGATAATGGAGTCGGACATCGGTGACG
TGATCGATCCCGCCGGGTGGCACGAGTGGAATGGACCCTTCGCGCTCGACACATTGTACTACG
GGGAGTATAAGAACACCGGGGCCGGTGCCGGCACCGCAGGCCGGGTCAAGTGGAACGGATA
CAGGGTTTTGACCGATGTCAGAGAAGCAGAGCAGTTTACTCCGGGGAACCTTCTTGGGGGTT
CGAATTGGTTGGGTTCTACTGGATTTCCCTACTCCCTGGGTCTGTCATCTTGA
F: GGAAGGAACACTCAAGGAC
R: AACTGCTCTGCTTCTCTGAC

E3.2.1.67
3.2.1.67: galacturan 1,4-alpha-galacturonidase
>Cluster-24380.48143;orf1 len=348 frame:3 start:1410 end:1757 gi|743889216|ref|XP_010910801.1| PREDICTED: exopolygalacturonase-like [*Elaeis guineensis*]
ACATTGCAAAGTCCTCCCCACTTCGGTCAAGTTCGTTGTCACATCAAACACTCTGGTACAGAA
CATCAAATCAAGTTTTTCCACATAGCTCTGGTTGGCTGCAAGAACTTCTGGGGCAAAAATATC
CAGATCACCGCCCCTTCAAACAGCCCCAACACCGACGGAATTCACATTGAACGGAGCAGAGG
CGTAACTATATATAACTCGGTGATCGGAACTGGTGATGATTGTATCTCCATTGGACATGGTAACT
CACAAGTATTACTGAGTGGCATCAGTTGTGGACCAGGGCATGGGATCAGGCAAGTCCTCTTGA
AGTTCTCCTGCTACAATATTGTTATGTTCTGA
F: CTTCGGTCAAGTTCGTTG
R: AGAGGACTTGCCTGATCC

GAE
5.1.3.6: UDP-glucuronate 4-epimerase
>Cluster-24380.23129;orf1 len=1416 frame:1 start:238 end:1653 gi|743767574|ref|XP_010914055.1| PREDICTED: UDP-glucuronate 4-epimerase 1 [*Elaeis guineensis*]
AGGGGAATAGGAGGAACAGAGACGAAGAAGACGAAGACGGTGACGGAGAAGAAGATGAGG
ATTCTGGAGGACGAGTTGTTCCCATCGACGCCGGGGAAGGTAAAGATCGAGCGAGCCCACGC
GATTAATCGGCAGTTCAACCGGTGCTTCGCGTCGACGAGCACCATGTTCCTGTGGGCGCTCTT
CCTGATCGCGCTCACGGCCTCGTACCTCAGCTTCCAGAGCTTCGTCGACACTTCCTCCAAGTA
TTTCTCGGCGTCGTGGGGTGGCCTCCATTGGGAGAAGCAGATCCGGGCCTCCGCCGAGCCCC
GACGGTCGCAGGGATTCTCCGTCCTCGTCACCGGCGCCGCCGGCTTCGTCGGCACCCACGTC
TCCCTTGCCCTCCGCAAGCGTGGCGACGGCGTCGTCGGCCTCGACAACTTCAATTCCTACTAC
GATCCATCCTTGAAGAAGGCGAGGAAGTCCCTCCTCGCCTCTCACGGCATCTTCGTCGTTGAG
GGCGACATCAACGACGGCCGCCTCCTGGCCAAGCTCTTCGACGTCGTCCCATTCACCCACGT
GATGCACCTCGCCGCCCAGGCCGGAGTGCGGTACGCGATCGAGAACCCGGCGTCCTACGTCC
ACAGCAACATCGCCGGACTGGTGACGCTTCTGGAGGCGTGCAAGGCGGCGGACCCCCAGCC
GGCGATCGTATGGGCCTCCTCGTCGTCGGTTTACGGCCTGAACGAGAAGGTCCCCTTCTCGGA
GTCGGACCGCACCGATCGCCCAGCTTCCCTCTACGCCGCCACCAAGAAGGCCGGCGAGGAGA
TCACCCACACCTACAACCACATTTACGGCCTCTCGATCACCGGCCTCCGCTTCTTCACCGTCTA
CGGCCCCTGGGGCCGCCCCGACATGGCCTACTTCTTCTTCACCAAATCCATCCTCTCCGGCAA
TGCCATTACCCTCTTCAAGACCCGCGACGGCGCCGACGTCCAGCGCGACTTCACCTACATCGA

CGACGTCGTCAAGGGCTGCCTCGGCGCGCTCGACACCGCCGAGAAGAGCACCGGCAGCAGG
GGCGGCAAGAGGCGCGGTGCCGCGCAGCTTAGGGTCTACAATCTCGGCAACACGTCGCCGGT
GCCGGTGGGGAAGATGGTTGCTATCTTGGAAGAGTTGTTGGGGAAGAAGGCGAAGAAGAAC
GTCGTGACGATGCCAAGGAACGGCGACGTGCCGTACACCCATGCGAACGTTAGCTTGGCGGA
GAAGGACTTCGGATACCGGCCGACGACCGACCTCACTGCCGGGCTCCGGCGGTTCGTGAAAT
GGTACGTGGAATACTACGGGGTGACGGCTTCCCATGCTAAGGGGAAGGGGAAGGGTGGTGTC
GGCGGGGAGCGGAAGCAAGTGGATACGGCGGGGGTTGCGTCCGCGTAG
F: GTCGACACTTCCTCCAAGTA
R: CAAGGATGGATCGTAGTAGG

xynB
3.2.1.37: xylan 1,4-beta-xylosidase
>Cluster-24380.74951;orf1 len=699 frame:-3 start:915 end:217 gi|743839636|ref|XP_010937054.1|
PREDICTED: beta-xylosidase/alpha-L-arabinofuranosidase 2-like [*Elaeis guineensis*]
TGTCACCATCTTGGTTGTAGGCACCGATCAATCGATCGAGAGGGAGGGCTTGGATCGGGTGAA
CCTGCTCCTCCCTGGGCAACAGCCAACCCTTGTAACAGAGATAGCAAAGGCGGCGAAGGGA
AAGGTGGGAGGTTGCCTGTGACATGGTACCCTGAATCATTCGCCGACAAGGTCCCGATGACC
GACATGCGCATGAGGCCTGATCCATCGACTGGTTATCCTGGACGGACATACCGGTTCTATACGG
GTGATGTTGTATACTCTTTCGGTGATGGTTTAAGCTACAATGACTACGCCCACCACCTAGTTCG
AGCTCCAAAGCTAGTATCAATCCCCCTAGAAGAGGGGCACTCATGCTACTCCAAGAGGTGTAA
GTCGATTGACGTCGTCGGAAGTGGATGCGAGAATGTGGCCTTCGACATCCATCTGAGAGTTCA
GAACACCGGTCGCATGGCCGGGAGCCACACTGTCTTCCTTTTCTCAACTCCGCCTTCAGTCCA
CAACTCTCCCCAAAAGCAGTTGCTTGGATTTGAGAAGGTCTACTTGGGCCCCCAAGGGGTGG
GCAACGTTGTGTTCAAGGTTGATGTTTGCAAGGACTTGAGCCTTGTGGATGAGCTTGGGAAC
AGAAAGGTCGCCTTAGGGTCTCATATCCTTCATGTGGGGAGTTTGATGCATTCACTTAAGGTG
AGGGTTTAG
F: GACATACCGGTTCTATACGG
R: GAACTCTCAGATGGATGTCG

uxs
4.1.1.35: UDP-glucuronate decarboxylase
>Cluster-24380.33815;orf1 len=1449 frame:2 start:2 end:1450 gi|743784429|ref|XP_010921662.1|
PREDICTED: UDP-glucuronic acid decarboxylase 2-like isoform X1 [*Elaeis guineensis*]
CCCTTCTTTCCAACAGCAAAGGGGCAAAAGGGAGCGGAGATCCTTTGCGGATACCCCCCTCG
AAATATCTTCCCTTTACGTTTTAGTCTCTCGTCCTCTTCCGCCGCGACCGCCGAGATGGGCTCG
GAGCTTATCTATCGAGGCCATGATCACCAGCCCCAGGAGGGCGCCGGCGGGTACTCGCCGAA
GCCCCTGAAGCAGTGGTGGTGGATCTCGAGGCCACTCCGCTACCTCCTCCGCGAGCAAAGGC
TCCTCTTTGTGCTCGTCGGAATGGCCCTAGCCACCCTCGTCGTCTTCCTTCTACCCCGGGCTTC
ACCGGCCGCGGGATCCGCCATCGGAGCCCACCTCATGGTCGACCTTGCCCGTCGCTCCACCG
CCGCCGTCGGCGGCGGAGGGCACTACAGCTACCGGACGGCGCTCCCGGCGAGAGGATTTGTC
GGGGGGAAGGTGCCGCTGGGGCTGAAGCGGAAGGGTCTGAGGATCGTGGTGACCGGCGGTG
CGGGATTCGTCGGGAGTCATCTCGTGGACCGTCTGATTGCTCGCGGGGACAGCGTAATCGTGG
TGGATAACTTCTTCACCGGGAGGAAGGAGAATGTGATGCACCACTTCGGAAACCCTAACTTC
GAGCTCATCCGCCATGATGTCGTGGAGCCGCTCCTCCTCGAGGTCGACCAGATCTACCACCTC
GCCTGCCCGGCTTCTCCCGTCCACTACAAGTTCAATCCCGTCAAGACTATCAAGACCAACGTA
GTCGGAACTCTCAACATGCTCGGCCTCGCCAAGAGGGTCGGCGCCCGTTTCCTTCTCACCAG
CACCAGCGAAGTCTATGGCGACCCTCTCCAACACCCCCAGGTTGAGACCTATTGGGGCAATGT
CAATCCCATTGGTGTTCGGAGCTGCTACGACGAGGGCAAGCGTACGGCCGAGACTTTGACCA
TGGACTACCATCGCGGTGCCCAAGTTGAGGTGAGGATTGCTCGGATCTTTAACACATACGGGC
CTCGCATGTGCATCGACGATGGCCGAGTCGTCAGCAACTTCGTGGCCCAGGCGCTGAGGAAG
GAGCCAATGACGGTATACGGGGATGGGAAGCAAACCAGAAGTTTCCAGTACGTCTCAGATCT

GGTCGAGGGGTTGATGAGGTTGATGGAAGGGGAGCACGTTGGGCCTTTCAACCTTGGGAATC
CTGGTGAATTCACAATGCTCGAGCTCGCCAAGGTGGTGCAAGAGACAATTGACCCCAATGCC
CGGATCGAGTTCCGGCCCAACACCGAGGATGACCCCCACAAGCGCAAGCCTGACATCAACAG
GGCGAAGGAACAGCTTGGCTGGGAGCCTAAGATCGCCCTCCGCCAGGGCCTTCCTCTCATGG
TCTCTGACTTCCGCAAGCGAATCTTTGGCGACCACACGGGCTCTGATGCCACCACCACTGGA
ACCGGCACTGCTTGA
F: CCAGAAGTTTCCAGTACGTC
R: GTCAGAGACCATGAGAGGAA

UGDH
1.1.1.22: UDPglucose 6-dehydrogenase
>Cluster-24380.61743; len=1062 start:1716 end:657; minus strand
XXTGTCCCAGTGAAGACTGCTGAGGCCATTGAGAAGATCCTAACCCACAACAGCAAAGGAAT
CAACTACCAGATCCTCTCCAACCCAGAGTTCCTTGCAGAGGGTACGGCAATCCAGGACTTGTT
AAAACCTGATCGGGTACTAATCGGCGGCCGGGAGACTCCGGAGGGCTGCAAGGCTGTTCAGG
CACTGAAAGCTGTGTATGCCCATTGGGTGCCTGAGGATCGGATCCTAACAACCAACCTGTGGT
CTGCAGAGCTGTCCAAGCTCGCAGCCAATGCCTTCTTGGCACAGAGGATCTCCTCTGTCAAC
GCCATTTCGGCACTCTGCGAGGCCACTGGGGCCAATGCGTCTGAGGTGGCCTATGCCGTGGG
GAAGGACTCGAGGATTGGTCCTAAATTCTTAAATGCCAGTGTTGGGTTTGGAGGGTCCTGCTT
CCAGAAGGACATCCTTAATCTGGTCTACATCTGTGAGTGCAATGGTCTGCCTGAGGTGGCTAA
CTACTGGAAGCAGGTCATCAAGATCAATGACTACCAGAAGAGCCGGTTTGTGAACCGGGTTG
TGTCCTCCATGTTCAATACCGTTTCTGGCAAGAAGATTGCGGTGCTCGGGTTTGCTTTTAAGA
AAGACACCGGTGACACGAGGGAGACCCCGGCAATTGATGTTTGCAAGGGTCTCTTGGGGGA
CAAGGCCACAATTAGCATCTATGATCCGCAGGTGACCGAGGACCAGATCCAACGTGACCTTGC
GATGAACAAGTTCGACTGGGACCACCCGATCCACCTGCAGCCGATGAGTCCGACAGCCGTCA
AGCAGGTGTCGGTGACATGGGATGCTTATGAGGCTACCAAGGGGGCCCATGGTGTCTGCATCC
TGACCGAGTGGGATGAGTTTAAGACCCTGGACTATCAGCGGATCTATGACAACATGAAGAAG
CCAGCTTTCATATTTGATGGGCGCAATGTGGTGGATCCAGAGAAGCTTAGGGAGATTGGATTC
ATTGTGTACTCAATTGGGAAGCCACTGGACCCATGGCTCAAGGATATGCCCGCTGTTGCCTAA
F: CTAACAACCAACCTGTGGTC
R: TTCCAGTAGTTAGCCACCTC

SPS
2.4.1.14: sucrose-phosphate synthase
>Cluster-24380.13417;orf1 len=1989 frame:2 start:2303 end:4291 gi|743806522|ref|XP_010927691.1| PREDICTED: probable sucrose-phosphate synthase 2 [*Elaeis guineensis*]
AAGTTGCAGGTTATTCCTCCTGGTATGGATTTCAGTAATGTTGTAGTTCAAGAAGACACAACT
GATGCTGATGGAGATTTAAAAGAACTTATAGGTTCAGAGGGAACTTCACCTAGAGCAGTCCCA
CCTATATGGTCTGAGGTGATGCGTTTCCTCACAAATCCTCACAAGCCGATGATCCTGGCCCTAT
CAAGGCCAGACCCAAAAAAGAATATCACTACCCTTGTGAAGGCCTTTGGAGAATGCCGTCCA
TTAAGAGAGCTTGCAAATCTTACATTGATAATGGGAAACAGGGATGACATAGATGAGATGTCC
GGTGGCAATGCTAGTGTCCTCATGACAGTTTTGAAACTGATCGATAAATATGACCTGTATGGAC
TAGTTGCTTTCCCCAAGCATCATAAGCAATCAGATGTTCCAGAGATATACAGGCTTGCAGCAA
AAACAAAAGGAGTCTTTATAAATCCTGCTCTGGTTGAGCCTTTTGGACTGACTCTAATTGAGG
CAGCTGCACATGGGCTTCCAATGGTGGCAACCAAAAATGGAGGTCCTGTCGACATTCATCGG
GCACTAAACAATGGCTTGCTCATAGACCCACATGACCAGAAAGCCATAGCTGATGCCTTGCTA
AAGCTAGTTGCAGACAAGAACCTATGGCATGAATGTAGGAAAAATGGGTGGCGGAATATTCAT
CTCTTTTCATGGCCCGAGCACTGCAGGACATACCTTACGAGAGTAGCTGCATGTAGGATGAGA
CATCCACAATGGCAGACAGACACTCCAACAGATGATATGGTTGTTGATGTTGAGGAGTCATTT
GGAGACTCCCTCAAGGATGTCCAAGAGTCATCTCTAAGGCTATCTGTAGATGGTGAGAAAAGC
TCCCCTAATGGCTCCTTGGAACACAACCAGGCTGAGTTCGAGAAGGTGGCTGAAGGGAAGG

GTGACACTGAGGTACAAGATCAAGTGAAGAAAATTCTGAACAAGATCAAGAAACAGGTGCC
TGAACCACAGGCTACTGGTAGCAGTAAGAAACAAACTGAGGTTTCAGGACAGACAATTAACA
AGTATCCTCTACTTCGAAGGCGTCGGAGACTATTCGTGATAGCTCTAGACTCGTATGACAGCA
AAGGAGCTCCTGAAAAGAAGATGTTGCAGGTGATACAGGAGGTCTTTAGGGCTATTCGATCG
GATTCTCAGATGTCGAGGATTTCAGGCTTTGCTCTCTCAACTGCTATGCCCATTTCTGAGACAC
TAGAGTTATTGAAGTCAGGGAAGATTCTTGCCACTGATTTTGATGCTCTAATCTGCAGTAGTGG
AAGTGAGGTCTACTATCCAGGTACTTCTCAGTGCATGGATGCAAATGGCAGGTTATGCGCAGA
CCCAGACTATGCCACCCACATTGAGTACCGTTGGGGTTATGATGGTGTAAAGAGGACTTTAAC
AAAATTGATGGCTTCTCAGGATGGTCAAGGTGATAGCAAACCCGAGAATTCTTCAAGCAACAT
AGAGGAAGATGTGAAGTCAAGCAATCCTCATTGTGTCTCTTTCTTCATAGAGGATTCAACCAA
GGCAAAGCCTGTCGACGATCTACGTCAGAAGCTCAGGATGCGTGGACTGCGATGTCATCTCAT
GTACTGCAGGAACTTGACGCGATTGCAGGTTATTCCTCTTCTGGCATCTCGGTCTCAAGCCCT
CAGGTACCTATTTGTCCGTTGGGGTCTGAACGTTCTGAACATGTATGTGATTGTTGGAGAGAA
AGGTGACACAGATCATGAGGAGCTCATATCAGGATCTCACAAGACAGTTATCATGAAGGGAGT
TGTCGAAAAAGGTTCAGAAGAGCTACTTAGAACAGCAGGGAGCTACCAGAAGGAAGACATT
GTTCCTGGTGAGAGTCCCCTTATTGTCTATACCAACAATGGAATTAATTCAGAGGAGATCATGA
AGGCACTGAAAGAAGCATCGAAGGCTGCTTCAGGGTTGTAA
F: CGGAGACTATTCGTGATAGC
R: GACCTCACTTCCACTACTGC

MGAM
3.2.1.20: maltase-glucoamylase
>Cluster-24380.19567;orf1 len=2556 frame:-1 start:2644 end:89 gi|695071127|ref|XP_009382622.1|
PREDICTED: neutral alpha-glucosidase C [*Musa acuminata* subsp. malaccensis]
AATAGGAGTTATTATAAAAACCGAACTTTGTCAAATCATATAAAACTTGGTAATTTGCTCATGAT
AGTTGAAATTTCTCTTGTGATCTATATTGGTTTTACCATTACAATTTGGTTTCCATATCTATTCTT
CTCCATTAAAATTTTAGGAACCATTTTTATGCCTCCCAAATGGTCACTTGGATATCATCAATGTC
GTTGGAGCTATGACTCTGCTGCAAAAGTTCTCAAGGTTGCCAGAACATTTCGAGAGAAACAC
ATACCTTGTGATGTCATATGGATGGACATTGACTATATGGATGGCTTTCGATGTTTTACATTTGA
CAAAGAATGCTTCCCTGATCCAAAATCCATGGTAAACGACCTTCATTCCATTGGCTTCAAAGC
AATCTGGATGCTTGATCCTGGGATTAAACATGAAAAGGGTTACTTTGTCTATGATAGTGGTTCT
CAAAGTGATGTCTGGATACTAAAAGCAGATGGGAAGCCTTTTGTTGGGGAAGTGTGGCCCGG
CCCTTGTGCTTTTCCTGATTTCACACAAGAAAAAGCACGTTTTTGGTGGGCTAATCTAGTGAG
GAACTTCATGTCTAATGGAGTTGATGGTATATGGAATGATATGAATGAGCCTGCTGTTTTCAAG
TCGGTAACAAAAACAATGCCAGAGAGCAACATTCACAGAGGAGATACTGAACTTGGTGGTCG
CCAAAATCACACTCATTATCACAATGTTTATGGCATGCTGATGGCAAGATCAACTTATGAGGGA
ATGAAAATGGCTAGTGGGACTAAACGACCATTTGTTCTCACCAGAGCTGGGTTCATAGGAAGT
CAACGTTATGCTGCAACCTGGACTGGAGATAACCTATCAAACTGGGAGCACCTACATATGAGT
ATACCTATGGTGCTTCAATTGGGGCTCAGTGGTCAGCCATTATCAGGACCAGATATTGGTGGGT
TTGCTGGAAACGCAACTCCAAAACTTTTCGGCCGATGGATGGCAGTAGGTGCTTTGTTTCCCT
TTTGTCGTGGGCATTCTGAGTCTGGGACCATCGACCACGAACCATGGTCTTTTGGAGAAGAGT
GTGAAGAAGTATGCCGCCTTGCATTGATGAGGCGCTATCGGCTTATACCACACATATATACTCT
TTTCTATATGGCTCATACAAAAGGAACTCCTGTTGCGGCTCCCACTTTTTTTGCTGATCCACGA
GATTCTCGTTTGAGGGCGGTTGAAAATTCCTTTCTGTTGGGACCACTTTTAATTTGTGCCAGCA
CTGTACCTGAGCAATGTTCACATGAACTGTCACATGTGCTGCCAAATGGAATCTGGTTGCGCT
TTGATTTTGGTGATTCACATCCAGATTTGCCAACTTTCTATCTGCAAGGTGGTTCAATTATCCCT
ACAGGTCCTCCTCTTCAACATGTCGGCGAGGCAAAACCAACTGATGAGATATCACTCATTATT
GCTTTAGACAAAGATGGGAAAGCTGAAGGTGTTGTCTTCGAAGATGATGGTGATGGATATGAG
TACACTCAAGGGAATTATCTCTTAACATATTATGTTGCCGAACTGCATTCTTCACTGCTGAAAG
TTAAAGTATCCAAGACTGAAGGATCATGGAAGAGGCCTAAACGTGCTCTCCATGCACAAATAC
TGCTGGGAGGAGGTGCCATGATTGATGCACGGGGTGTTGATGGAGAGGAACTACAGATTACA

ATGCCTCCGGAATCTGAAGTGTCTGACTTGGTGGCAGCCAGTGAAAATCAGTACCAAATGCG
CTTGGAAAAGGCCAAGTGTATCCCAGATGTAGATAGGCTTTCAGGACAAAAGGGCATTGAGC
TCTCGAAGACCCCTGTTGAACTAAAGAGTGGTGACTGGGTTCTTAAAGTGGTTCCTTGGATTG
GGGGTCGAATTATCTCTATGACGCATCTTCCTTCTGGTATCCAATGGCTTCACAGCAGAGTTGA
AGTTGATGGATATGAAGAATACAGTGGCGCTGAATACCGGTCTGCTGGATGCTCAGAGCAATA
TAAAGTTGTGCAGAGAAATCTTGAACAATCAGGAGAAGAGGAATCACTTTGCTTAGAAGGGG
ATATTGGTGGCGGGTTAATTCTCCAGCGCCAGATATCTTTTCCAGAAGATAATCCAAAGGTTAT
CCAAATTGATTCTAGAATTATAGCCCAAAATGTGGGAGCTGGTTCTGGTGGATTTTCAAGGTTG
GTTTGTTTGCGCGTGCACCCAACGTTCACTCTTCTGCACCCGACTGAAGTTTTGGTTGTATTCA
ATTCCGTTGATGGTTCCAAGCATGAAATAGGCCCTGAATCTGGAGAGCAATCATTTGAGGGGA
ATCTCCTCCCAGATGGGGAATGGATGCTGGTTGACAAATGTGCAGGACTGAGTCTCGTTAATC
GCTTTGACCCAAGCCAGGTAAGCAAATGCATGGTGCACTGGGGATCTGGAACAGCTAATCTA
GAGTTGTGGTCTGTAGAGAGGCCAGTGTCTAAAGACAGCCCTTTGAGGATTTGTCATGAATAT
GAGGTGAAGCAAAGCTTATAA
F: GAGAGCAACATTCACAGAGG
R: CATATGTAGGTGCTCCCAGT

SUS
2.4.1.13: sucrose synthase
>Cluster-24380.23100;orf1 len=2442 frame:-1 start:3063 end:622 gi|695052176|ref|XP_009414138.1|
PREDICTED: sucrose synthase 4-like [*Musa acuminata* subsp. malaccensis]
CCTCCAGCCATGGCGACACTCAAGCTCGGGCGCATCCCCAGCATGCGCGAGAGGGTGGAGGA
CACCCTCTCCGCCCACCGCAACGACCTCGTCTTTCTCCTCTCCAGGTATATGGGCAAGGGGAA
GGGAATCCTTCAGCCCCACGACCTCCTCGACGCCCTCGCCACCATCGACGACCACGGCCGCC
ACCACCTCTCCGAGGGCCCCTTCTTCGAGGTCCTCAAGTCCGCTCAGGAAGCCATAGTTCTGC
CCCCGTTTGTGGCCATCGCTGTGCGGCCGAGGCCTGGCGTGTGGGAATACGTGCGGGTTAATG
TGTACGAGCTCAGCGTCGAGCAGCTCAGCATCTCTGAGTACCTCCAATTCAAGGAAGAGCTC
GTCGACGAGCGTAGCTATAATGATCACTTTGTGCTGGAGCTCGACTTCGAGCCTTTTAATGCCT
CATTCCCACGGCCGAACCGATCGTCGTCCATTGGAAATGGGGTTCAGTTCCTCAACCGGCACC
TCTCGTCCATTATGTTTCGGAACAAGGAGTCCTTGGAACCCCTGCTTGATTTCCTCCGGGCCC
ACAGGTTCAAGGGTCATGTGTTGATGCTGAACGACAGGATACAGAGTGTGTCCAGGCTTCAG
TCTGTGCTGGCAAAGGCCGAGGAGCATCTGTCTAAGCTCCCACCTGCAACGCCATTCTCCGA
ATTTGCTCATGTGTTTCAAGAGATGGGCTTGGAGAAAGGTTGGGGTGATACAGCTGAACGAG
TGGTGGAGATGATCCATCTTCTGCTGGATATTCTTCAAGCTCCTGATCCCTCCACTTTGGAGAA
ATTTCTCGGGAGGATTCCCATGGTGTTCAATGTTGTAATAGTTTCCCCCCATGGGTATTTTGGCC
AAGCTAATGTATTAGGTTTGCCGGACACTGGAGGGCAGATTGTCTATATATTGGATCAAGTTCG
TGCACTAGAGAATGAGATGATTCTAAGAATGAAAAAACAGGGGCTGGATGTTGATCCCAGAA
TTCTTATTGTGACTAGGTTGATACCCGATGCAAAAGGGACAACATGCAATCAACGACTTGAGA
GAGTCATGGGAACACAACATACTTACATCCTACGGGTGCCTTTTAGGACTGAAAAGGGAATTC
TTCGGAAATGGATTTCACGATTTGATGTATGGCCTTACTTGGAAACCTTTGCAGAGGATGCAG
CTAGTGAAATGGCAGCGGAGTTACATGGCACTCCAGATCTGATTATTGGGAACTACAGTGATG
GAAATCTTGTTGCATCATTGTTATCTTACAAATTAGGAATTACCCAGTGCAACATTGCTCATGC
ACTTGAGAAAACAAAATATCCAGATTCAGATATATATTGGAAAAAGTATGACGACAAGTACCA
TTTTTCTTGCCAGTTTACCGCTGATCTAATCGCTATGAACAATGCAGATTTTATAATTACTAGCA
CCTACCAGGAGATTGCGGGAAGCAAGAACACAGTGGGACAGTATGAGAGCCATACTGCTTTC
ACTCTTCCTGGTTTGTATCGTGTTGTCCATGGGATTGATGTTTTTGATCCTAAGTTCAATATAGT
CTCCCCCGGAGCAGATATGGGTATTTACTTTCCATACTCAGAAAAGGAAAGGAGGCTTACTTC
TCTTCATGGTTCCATTGAAAAGCTGCTGTATGATCCAGAGCAAAATGACGAACACATTGGTTG
GTTGGATGACCGGTCTAAGCCCATCATCTTCTCCATGGCAAGGCTTGACCGGGTGAAAAACAT
AACAGGACTGGTTGAATGCTTTGCTAAGAACACTAAACTAAGAGAATTGGTAAACCTTGTTGT
AGTTGCTGGTTACAATGATGTGAAGAAATCGAGTGACAGAGAAGAAATTCAAGAGATCGAGA

AGATGCACCAACTTATAGAGGCCTATAACTTATTTGGCCAGTTCCGCTGGATTTCTGCCCAAAC
AAACAGAGCACGTAATGGTGAGCTATATCGTTACATAGCTGACACTGGAGGTGCTTTTGTGCA
GCCTGCTTTTTATGAAGCCTTCGGACTCACTGTTGTGGAGGCCATGACTTGCGGTCTTCCAAC
ATTTGCCACTTGTCATGGAGGGCCTGCAGAAATTATTGAGCATGGGATATCAGGTTTCCATATA
GACCCATATCATCTGGACCAGGCTGGTGCAATCATGGCCGACTTCTTTGAACGGTGCAAAAAA
GACTCTGGGTATTGGAAGCAGATATCCGATGGAGGGCTCCGTCGAATCCATGAAAGGTATACG
TGGAAAATCTATTCTGAAAGGCTAATGACGTTGGCGGGGGTTTATGGGTTCTGGAAGTATGTT
TCGAAGCTTGAAAGACGTGAGACGCGTCGTTATCTTGAGATGTTCTACATATTGAAGTTCAGG
GACTTGGTGAAATCTGTTCCCCTAGCGGTTGATGATGAACATTGA
F: GATACAGCTGAACGAGTGGT
R: TCGGGTATCAACCTAGTCAC

TPS
2.4.1.15: trehalose 6-phosphate synthase
>Cluster-24380.18262; len=1170 start:1168 end:1; minus strand
XXCTCCGACAACTCCGCCGGCCCGTCCTCCTCCCCCCGTGACCGCACCATCGTCGTCGCCAA
CCACCTCCCCATCCGCGCCCACCGCCGCCCCGACGGCCGCGGCTGGACCTTCTCCTGGGACG
AGGACTCCCTCCTCCTCCAGCTCAAGGATGCCATTGGAGATCACTGTGACATGGAATTCATCT
ACGTCGGCTGCCTTCGCGACGAGATCCCCCCCGCCGACCAGGACGAGGTCAGCCAGATCCTC
CTCGACACCTTCAAGTGCGTGCCGGCCTTCCTGCCGCCCGACCTCCTCGCCCGATTCTACCAT
GGCTTCTGCAAGCAGCAGCTCTGGCCGCTGTTCCATTACATGCTCCCGCTGTCGCCGGAGCTC
GGCGGCCGCTTCGACCGCTCCCTCTGGCAGGCCTACGTCTCCGTTAACAAGATATTCGCCGAT
AAGATTCTCGAGGTTATCAACCCTGATGACGATTTTGTCTGGGTCCATGACTACCACCTCATGG
TCCTGCCAACCTTCCTCAGGAAAAGGTTCAACCGCGTCAAGCTCGGATTTTTCCTCCATAGTC
CGTTCCCCTCGTCGGAAATCTATAAGACGTTGCCCGTCCGGGAGGAGCTCCTGCGTGCCCTGC
TGAACTCCGACTTGATCGGATTCCACACATTCGATTATGCTCGCCATTTTCTGTCCTGCTGTAG
TAGGATGCTCGGGCTGTCGTATGAGTCCAAGAGGGGTTATATTGGATTGGAGTATTATGGCCGG
ACGGTTAGCATTAAGATACTGCCTGTCGGAATTCATATGGGTCAGCTCCGCTCGGTGCTGAGC
CTCCCGGAGACAGAGGCGAAGGTGGCGGAGCTCATCGAGCAGTTCCGGGATCGGGGAAGGG
TGATGCTTCTCGGTGTCGACGACATGGATATCTTTAAAGGGATCAGCTTGAAGCTGCTGGCCA
TGGAGGAGTTGCTCAAGCAGCACGCCGAGTGGCGGGGGAAGGTGGTCTTGGTTCAGATCGC
TAACCCAGCGAGGGGGAGGGGGAAGGATGTGAGGGAAGTTCAGGAGGAGACCCATGCGACG
GCGAAGAGGATCAATGATGCCTATGGTGGGAATGGGTATGAACCTGTTATTTGATCAATGACC
CGCTGCAATTCTATGAGAGAATGGCTTACTATGTGGTGGCA
F: GTCCATGACTACCACCTCAT
R: AACCCCTCTTGGACTCATAC

TPP
3.1.3.12: trehalose 6-phosphate phosphatase
>Cluster-24380.18259; len=996 start:1 end:993
XXGGCTTACTATGTGGTGGCAGAGTGTTGTCTGGTGACGGCGGTGAGGGATGGCATGAATCTT
ATCCCTTATGAGTACATCATTGCTCGACAAGGGAATGAGAATTTGGATAGTGTCTTGGGTTTGA
ATCCTTCTTCGCCGAGGAAGAGTATGTTGGTGCTTTCGGAGTTCATCGGGTGCTCGCCGTCATT
GAGCGGGGCAATCCGGGTGAATCCATGGAATATAGAAGCGGTGGCTGATGCAATGGTCTATGC
CTTGGAGATGTCTGAGCCAGAGAAGCAGCTAAGGCATGAGAAACACTATAGGTATGTGAGCA
CACATGATGTGGGCTACTGGGCAAATAGTTTCTTGCAGGATTTGGCGAGGACGTGTAGGGACC
ATTCTAGGAGGAGGTGTTGGGGAATTGGGTTTGGTTTGGGGTTCAGGGTTGTGGCTCTTGATT
CAAACTTTAGGAAGCTGGCGATGGAGCACATTGTTTCAGCGTATAAGAGAACTAAAACTCGG
GCCATCCTGTTGGATTATGATGGTACTTTGATGCCGCAAGCGACGATCAATAAGAGCCCCAGCT
CAAGGTCCATCGAAATCTTAAATAGCTTGTGTTCCGATACGAATAATGTGGTCTTTCTAGTGAG
TGCTAGAAGTCGAAGCACTCTCAGCGAGTGGTTTTCGCCTTGCGAGAATCTGGGTATGGCTGC

AGAGCATGGCTACTTCCTCAGGTCGAAGAGAGATGCTGAGTGGGAAACATGCATTCCTGTTG
CTGACTGTGGCTGGAAGCAGATTGCGGAGCCTGTAATGAATCTATACACTGAAACAACTGATG
GTTCGACCATCGAAGATAAGGAAACTGCACTTGTCTGGTCCTATGAGGATGCTGATCCTGACT
TTGGGTCCTGCCAAGCCAAGGAGCTCCTTGATCATCTTGAGAGTGTTCTCGCAAATGAGCCGG
TATCGGTGAAGAGTGGTCCACACATTGTGGAGGTTAAACCACAGGGTGTX
F: GACGTGTAGGGACCATTCTA
R: CTGAGAGTGCTTCGACTTCT

BGL
3.2.1.21: beta-glucosidase
>Cluster-24380.19254;orf1 len=1548 frame:-2 start:1745 end:198 gi|743874038|ref|XP_010906974.1| PREDICTED: beta-glucosidase 1-like [*Elaeis guineensis*]
TTCTTGGTTTTAGATATGAGGTCCCTTGGCCACCTCCTACTCCTCCTCTTTGTTCTTGCGGTGGT
CCGGTGCTCCGCCGCCGGGCCTCCGGCCACCGCCCACCCGCCGTCGAGGGCCCGGTTCGACA
CTGGCGGGCTTAGCCGGGCGGCCTTCCCGGCCGGGTTCGTTTCGGGACGGCGGCGTCGGCG
TACCAGGTGGAGGGCATGGCGCTGAAGGACGGGCGCGGCCCCTGCATCTGGGACGTCTTCGT
TAAGATCCCAGGGAATATTGCAAATAATGCCACTGCGGACGTTTCAGTAGATGAATACCATCGC
TACAAGGAGGATGTGGACATCATGAAGAAGATGAATTTTGATGGGTACCGGTTCTCGATCTCA
TGGTCCCGGATATTTCCAAATGGAGTGGGGAAGGTAAATTGGAAGGGAGTGGCGTATTACAAT
CGACTTATAGACTACATGTTAAAGCAAGGCATTACACCGTATGCAAATCTCTATCACTATGACC
TCCCACAAGCACTTGAGGTGCAGTACAATGGGCTGTTAAGCGCAAAAATTGTGGAGGCATAT
GCAGATTATGCTGACTTCTGTTTCAAGACATTTGGTGATAGGGTGAAAAATTGGTTCACATTCA
ATGAGCCAAGGGTGGTGGCAGCTCTTGGATATGATGATGGTATGTTTGCCCCAGGAAGGTGTA
CAAACTGTTCTGCTGGTGGGAACTCTGCTACTGAGCCTTATATTGTTGCCCATAATCTCATCTTA
TCTCATGCTGCTGCTGTTAAGCGGTATCGCGACAAGTATCAGGCAACACAAAAAGGCAGGATT
GGAATTCTCCTGGATTTTGTTTGGTATGAACCTCTGACCAATTCGGAAGCAGACCAAGCAGCA
GCTCAAAGAGCAAGGGATTTTCATTTGGGATGGTTTCTTCACCCTATTGTATATGGTGAGTACC
CAAAGACATTGCAGGACATAGTTAAAGATAGGCTTCCAAAATTCACAGTGGAAGAGATTAAG
ATGGTGAAGGGCTCTATAGATTATGTGGGAATCAACCATTATACTTCTTACTACATGTATGATCC
GCATCTTCCTGAGCCACAAAAGCCCACTCGTTATGCGAATGATTGGAATTGTGGATTTGCTTTT
GAGCGCAATGGTGTACCTATTGGACCCCGGGCATATTCTTATTGGCTGTACATTGTACCATGGG
GGATGTACAAGGCAGTGACCTATGTTAAGGAACAATATGGCAACCCAACTGTGATCCTATCTG
AAAATGGAATGGATGATCCTGGCAATGTCACTTTACCAAATGGTTTGCATGACACAACAAGAA
TCAATTACTACAAGTCCTATATAACTGAGCTGAAGAAGGCAATTGATGATGGGGCAAACGTGA
TCGGTTACTTCGCTTGGTCTTTGCTTGATAACTTCGAATGGAAATTAGGATACACATCAAGATT
CGGGCTGGTCTATGTGGACTATAGAAACCTAAAGCGGTACCCTAAGATGTCAGCTTATTGGTTC
GGACAGATGCTTCAGAGGAACAAGTAG
F: CACCTCCTACTCCTCCTCTT
R: CCTGGGATCTTAACGAAGAC

FRK
2.7.1.4: fructokinase
>Cluster-24380.56530;orf1 len=1227 frame:2 start:269 end:1495 gi|743823905|ref|XP_010932642.1| PREDICTED: probable fructokinase-6, chloroplastic [*Elaeis guineensis*]
GTTTGTCGCCGGCGGACACCGGAGAGGAGCGTTAAAAGCCGTCCGATGGCTCTCCACGCTGC
TGCTTTCTCTTTTACCGGCCTCGCTTCAAACTCCCGACTCCGTGCCCAGAACTTCTCGGAGAG
GGGTGGTTTCAAAAGAGGATTTGTTAGAGCGGTAGCCGTTCCTCCTCTTTTCTCCTCGCCTCG
GCCTCTCAGTAGAATTTCAGGTTTTGGAGGGGCATTTGCAGGCAACAATAGCTCTCTCGAGAC
TAGTGATTCTTCACTTGTCGTGTGTTTTGGGGAAATGCTAATTGATTTTGTCCCCACGATTAGT
GGCTTATCATTAGGTGAATCTCCAGCTTTCAAAAAGGCTCCTGGAGGAGCACCGGCTAATGTT
GCTGTTGGCATAGCTCGTCTTGGTGGTTCATCAGCCTTTATTGGGAAGGTTGGTGAAGATGAG

TTTGGGTATATGCTAGCTGATATTTTAAAAGAAAATAATGTGAACAATCAAGGAATGCGGTTTG
ATCCGGGTGCTCGAACAGCTTTGGCTTTTGTTACATTGAGAAATGATGGTGAACGTGAGTTCA
TGTTTTATCGTAATCCCAGTGCTGATATGCTGCTCGAAGAGAAAGAACTTGACCTTGACATCAT
TAGGAAGGCAAAGATCTTCCACTATGGATCTATAAGTCTTATCACCGAACCATGTAAATCAGCA
CATATTGCAGCAGCTATGGTTGCTAAGGATGCTGGAGTGCTTCTTTCTTATGATCCAAATCTGA
GGCTTCCACTATGGCCATCTGCAGAGAGTGCTAGAGATGGAATCTTGAGCATATGGGATGCTG
CTGATATTATCAAGATAAGTGAAGAAGAAATTTCCTTTTTGACTAAAGGAGAAGATCCATATGA
TGATGCAGTTGTACGCAAGCTCTTCCACCCAAATCTTAAGTTGCTTCTTGTCACTGAGGGCCC
ACAGGGCTGCAGATATTATACCAAGGAATTCAGTGGGAGGGTAAGCGGACTAAAAGTGAATG
CGGTGGACACTACTGGTGCTGGGGATGCTTTTGTTGCTGGAATATTATCGCAATTAGCCACTGA
TATTTCCTTGCTCCAGGATGAAGGTCGACTAGTAGAAGCCCTTAGGTTTGCAAATGCATGTGG
TGCACTGACCGTGATGGAGAGAGGAGCAATCCCTGCTTTACCAACTCGAGATGCGGTGATGA
ATGCCTTGGTTAATATAGTTGCTTAA
F: GTTGCTTCTTGTCACTGAGG
R: GCAAACCTAAGGGCTTCTAC

GlgC
2.7.7.27: glucose-1-phosphate adenylyltransferase
>Cluster-24380.60456;orf1 len=1689 frame:2 start:2 end:1690 gi|743858996|ref|XP_010942522.1| PREDICTED: glucose-1-phosphate adenylyltransferase small subunit, chloroplastic/amyloplastic [*Elaeis guineensis*]
CGGAAGATGGAGAGGGAGTGGCCTTCCCTGCTCCTCTCTACAAGTAACGGAACTTCCCCCAC
GCACACTCTCTCTGTTCGAGAGGTCACTCGCATGGCGATGGCTGCGATGGGGATCTCGCGAAT
CCCGCTTTCCCAAACCCTAATCCCTTCGACCTCTCCCTCTCCATCTTCCGATCGGGTCTCCAAT
GATCCACCGCGGTGCCTCAAATCTTCTTCGAACCGAGGCCTCTCCTCCTCTTTCCACTTCTCCG
GCGAGGCACTCTTCTCAAAGGCCGTCCTTGGGCGCCGGAGAACGGCATCCGGGGGGAGGAC
GCCGGTTCTGGTTTCCCCCAAGGCTGTCTCGGACTCGAGGAGCTCGCAGACGTGTCTCGATC
CTGACGCCAGTCGTAGTGTTTTGGGAATAATACTTGGTGGTGGAGCTGGAACCCGCCTGTACC
CTCTAACAAAGAAGAGAGCCAAACCAGCAGTTCCTTTAGGAGCCAACTACAGGCTGATAGAC
ATACCTGTCAGCAACTGCTTGAACAGTAATATTTCAAAGATCTATGTTTTGACGCAATTCAATT
CTGCTTCGCTCAATCGCCACCTTTCACGAGCTTATGGAAGTAACATGGGTGGATACAAGAATG
AGGGGTTCGTGGAAGTCCTTGCTGCTCAGCAGAGTCCAGAGAACCCAAACTGGTTTCAGGGT
ACAGCTGATGCTGTAAGACAATATCTTTGGCTTTTTGAGGAGCACAACGTTATGGAGTTTCTTG
TCCTTGCTGGAGATCATTTGTATCGCATGGACTATGAGAGGTTTATCCAGGCACACAGAGAAA
CAGATGCGGATATCACGGTGGCTGCACTTCCCATGGATGAAAGGCGTGCAACTGCTTTTGGCC
TGATGAAGATTGATGAAGAAGGACGCATAATTGAATTCGCAGAGAAGCCAAAAGGGGAGAA
GCTGAAAGCGATGAAGGTCGATACTACCATTTTAGGCCTTGATGAGAAAAGAGCAAAAGAGA
TGCCATATATTGCTAGCATGGGCATCTATGTGATCAGCAAAGACATAATGTTGCAGCTACTTCG
GGAAAAGTTTCCAGGAGCTAATGATTTTGGAAGTGAAGTAATTCCTGGTGCTACCAATGTTGG
AATGAGGGTGCAAGCATACTTGTATGATGGCTATTGGGAGGACATTGGGACCATTGAGGCATT
TTATAATGCAAATCTGGGAATAACTAAGAAGCCAGTACCAGATTTCAGCTTTTATGATCGTTCA
TCTCCAATTTACACACAAGCTCGATATTTACCACCTTCCAAAATGCTTGATGCTGATGTTACAG
ATAGTGTTATTGGTGAGGGATGTGTAATTAAAAACTGCAAGATTCACCATTCTGTAGTTGGGCT
TCGTTCTTGTATTTCTGAAGGTGCAGTCATAGAAGACTCATTACTGATGGGAGCAGATTATTAT
GAGACGGATCAAGAGAAGAGGTTCCTAGCTGCAAAAGGTAGCGTTCCAATTGGCATCGGAAA
GAATTCTCACATCAAAAGAGCCATCATAGACAAGAATGCTCGCATTGGAGAAAATGTCAAGAT
CATCAACGGCAATGATGTGCAAGAAGCAGCAAGGGAGACCGATGGATACTTCATCAAGAGCG
GTATCGTCACCATAATTAAAGATGCTTTGATCCCGAGTGGAACTGTCATATAA
F: CTCCTCCTCTTTCCACTTCT
R: CCATAAGCTCGTGAAAGG

SS

2.4.1.21: starch synthase

>Cluster-24380.44636;orf1 len=1818 frame:-2 start:4048 end:2231 gi|743771373|ref|XP_010915972.1| PREDICTED: soluble starch synthase 1, chloroplastic/amyloplastic isoform X1 [*Elaeis guineensis*]
TTCTCTCCTCTTTTCTGGTCCCCAGAAATGGATGCGGCTCTCCGCGTGTGCTCCTCCCCCGTCG
CTCCCCGCTGCTCCGCGAAGCCCTCGCCGTTGGATCCGTCCATATGTCAGCCCACGGCCGTCG
GATTGCCCTGCTGGAAAAGGCCGGCGTGCGGGCCCCTGTTCACAAGGAGGCGGTGCGTGGC
GGAGCGCAGCAAAGAGGACCCGCCGGTCCCCGCCCTCGCAGAAACAGAGGACGAGAAAAC
GCAGTCCGATGGTGACACAGAAGTCTCAGTTTCTAGCACTGCTGTTATTGATAATAGAGAGGA
AGAGGAAGAAGCTGAAGACACCAAAGAAGAGGAGCAACCGGAAACTAGATTAACATATAAT
ATTGTTTTTGTCACTGCTGAAGCCGCACCTTACTCAAAGACGGGAGGACTAGGAGATGTCTGT
GGTTCCTTACCTATTTCTCTTGCATCTCGTGGTCATCGTGTGATGGTAGTGTCTCCAAGATATTT
AAATGGGGATTCGGATAAAAATTTTGCTAAGGCAGTTGATGTTGGAAGACGCATTAAAATCCC
ATGCTTTGGTGGAGAGCAGGAAGTCGCATTTTTTCACGAATATAGGGCTGGCGTTGATTGGGT
GTTTGTTGATCATTTTTGTTATCATAGAGCTGGAAACCCGTATGGTGACAGATATGGAGCTTTT
GGTGACAATCAGTTTAGGTATACATTGCTTTGCTACGCAGCATGTGAGGCTCCATTAGTACTTA
CATTGGGAGGTTACATGTATGGCCAGAATTGCTTATTCCTTGTCAATGATTGGCATGCAAGCCT
TGTTCCGGTCCTTCTAGGGGCAAAATACCGTCCGCATGGTGTTTATAAAGATGCAAGGAGCAT
TCTTGTCATACATAACTTGGCACATCAGGGGTGGAAGCAGCAACCACATATCAGAACCTGGGA
TTGCCTCCTGAGTGGTATGGAGCTCTAGAGTGGGTGTTTCCCCCATGGGCAAGGACACATGCA
CTCGACAAGGGTGAGGCTGTCAATATTCTGAAGGGTGCAATTGTGACAGCTGATCGGATAGTG
ACAGTTAGTCAGGGTTACTCTTGGGAGATTACAACTGTTGAAGGTGGGCATGGTCTCAATGAG
CTTCTTAGTAGTCGAAAGAGTGTACTGAATGGAATCACAAATGGTATTGATGTTGATGACTGG
GACCCTGCTTCAGACAAATATATCCCATTTCATTACTCAATCGATGATCTCTCTGGAAAGGCTC
AATGCAAAGCAGCATTACAGAAGGAATTAGGACTTGCCATTAGGCCTGACTGCCCACTGGTTG
GATTCATTGGAAGATTGGACTACCAAAAGGGCACTGATCTAATTAGGTTAGCCCTGCCTGAGC
TTCTGCAAGACGACATCCAGTTCATTATGCTTGGATCAGGGGACCCAGATACTGAAGATTGGA
TGAGATCTGCAGAGTCTACCTACAGAGAAAAGTTCCGTGGATGGGTTGGATTTAATGTCCCTA
TTTCCCACCGGATCACTGCAGGGTGCGACATATTCTTGATGCCCTCCAGATTCGAGCCATGTGG
TCTTAATCAGTTGTATGCAATGAGATATGGAACCGTACCAGTCGTCCACTGCACTGGTGGCCTC
AGAGATACGGTGGAGAATTTCCACCCATTTGCTAAAGAAGATAGTGGTCAGGGGACGGGGTG
GACTTTTTTTCCATTATCAAAAGACAGTATGTTGGGGGTAAGTTTAATTGTTCATTATTCACCAA
AGATCTTTTTAAAATTCAGGAGACTGGCACCAGTTCTCATTTCATCATAG
F: TGGTGACACAGAAGTCTCAG
R: CACTACCATCACACGATGAC

PGM

5.4.2.2: phosphoglucomutase

>Cluster-24380.49157; len=978 start:1572 end:596; minus strand
ATGTCCTGCAGGATTGCATTTXCAAATGGTGAGCCTTTAGAAGATTTTGGGCATGGACATCCA
GACCCTAATCTTACGTATGCTAAAGATCTGGTTAACATTATGTATGCTGTGAATGCACCTGATCT
AGGAGCTGCAAGTGATGGTGATGGTGATAGAAACATGATTCTTGGAAGAGGCTTTTTCATTAC
ACCATCAGATTCTGTTGCAATTATTGCAGCTAATGCACAGGCGGCCATTCCCTACTTTAAAAAT
GGTCCTAAGGGTCTTGCCAGGTCTATGCCAACTAGTGGTGCTCTTGATTGTGTTGCAGCAAAA
CTGAATGTTCCATTCTTTGAGGTTCCTACTGGCTGGAAATTCTTTGGGAACTTAATGGATGCCG
GGAAGATATCTATCTGTGGGGAAGAAAGTTTGGAACTGGATCTGATCACGTTCGTGAGAAGG
ATGGCATATGGGCTGTTTTGGCTTGGCTTTCTATCATTGCTTACCGCAACAAGGACAAAAAGGT
TGGGGAGAAGTTGGTCTCAGTTGCAGATGTTGCTAAAGAGCACTGGGCAACCTATGGCAGGA
ACTTCTTTTCTAGATATGATTATGAGGAATGTGAATCTGATGGAGCCAATCAGATGATGGAGTA
CTTGAGGGACCTGATTTCTAAAACCCAGTCTGGAGAAAAATATGGAAATTATATCCTTCAATTT
GCTGATGACTTTAGCTATACTGATCCGGTTGATGGCAGTGTAGCATTGAAGCAAGGCCTCCGC

TTTGTATTTACAGATGGATCAAGGATTATATATCGTTTATCGGGCACTGGATCAGCAGGTGCAA
CTATCCGCATTTATATTGAACAATTTGAGCCTGATGTCTCCAAGCATGAGATGGATGCACAAAT
AGCATTGAAGCCCTTGATAGATTTGGCATTATCAATTTCAAAGCTCAAGGAGTTCACTGGCAG
GGAGAAGCCCACAGTTATCACATAA
F: GGACATCCAGACCCTAATCT
R: AGCACCACTAGTTGGCATAG

GPI
5.3.1.9: glucose-6-phosphate isomerase
>Cluster-24380.58951;orf1 len=1581 frame:-1 start:4192 end:2612 gi|695040240|ref|XP_009407687.1| PREDICTED: glucose-6-phosphate isomerase, cytosolic [Musa acuminata subsp. malaccensis]>gi|695040242|ref|XP_009407688.1| PREDICTED: glucose-6-phosphate isomerase, cytosolic [*Musa acuminata* subsp. malaccensis]
TCTCTGGACTGCGAACGAGAACTCTCATCTCATCTCATCTTCCTTCCGCTTCCGGAGGAAGTG
TTTTCAATGGCTTCAACGACTCTTGTTTGTGACACGGAGCCTTGGAAGGACTTGAAGGGTCAT
GTTAAGGAGATAGAAAAGACACATTTACGTGATTTGATGAATGATGTTGAGCGGTGCAAGTCA
ATGATGGTAGAGTTTGATGGAATACTTTTGGACTATTCCCGGCAACGTGTTCGTCAAGACACT
GTGGAAAAGCTATTCAAACTGGCTGAGGCAGCACATCTTAAACAAAAGATCGACAGAATGTA
CAGTGGAGAGCGTATAAACAGTACAGAGAATAGATCTGTGCTGCATATAGCTTTGCGAGCCCC
AAGGGATAAAGTTATTTGCAGTGATGGGAAAAATGTGGTACCAGATGTTTGGACTGTTTTAGA
CAAAATCAAAGACTTTTCAGAAAGGGTCCGAAGTGGTTCATGGGTTGGAGCAACAGGAAAG
GTATTAAAAGATGTTGTGGCTATTGGTATTGGTGGCAGCTTTTTAGGCCCACTATTTGTGCATAC
AGCCCTTCAGACAGATCCAGAGGCTGCAGAAAGTGCAAAAGGACGACAACTGCGATTTCTTG
CAAATGTAGATCCAATTGATGTTGCTCGTAACATCACTGGGTTGAATCCTGAAACAACCTTAGT
TGTGGTGGTTTCAAAGACTTTTACGACAGCTGAAACTATGCTGAATGCTCGAACATTGAAGGA
GTGGATTCAAGCTGCTCTTGGGCCTCAGGCAGTTGCAAAGCATATGGTTGCTGTCAGCACAAA
TATTCCGCTTGTGAAGGAGTTTGGGATTGACCCTGCCAATGCTTTTGCATTTTGGGACTGGGTT
GGTGGTCGCTATAGTGTTTGCAGTGCTGTTGGTGTGCTGCCTTTATCTCTCCAATATGGTTTTCC
AATTGTCCAGAAATTTCTGAATGGAGCTTCTAGCATCGACAACCATTTCCATTCAACTTCATTT
GAAAAGAATATACCTGTACTCCTAGGTTTGTTGAGTGTATGGAATGTTTCTTTCTTGGGATATCC
TGCGAGAGCCATATTGCCGTACTCTCAAGCACTCGAGAAATTTGCTCCACATATACAACAGGT
TAGCATGGAGAGTAATGGAAAAGGTGTATCCATTGATGGTATTCCCCTTCCCTTTGAGGCTGGT
GAAATAGATTTTGGAGAACCTGGAACAAATGGTCAGCATAGCTTCTACCAGCTAATCCATCAG
GGAAGAGTTATTCCTTGTGATTTCATCGGTATTGTAAAAAGCCAGCAACCTATTTACTTGAAAG
GGGAAGCAGTGAGTAACCATGATGAGCTGATGTCAAATTTTTTTGCACAGCCAGATGCTCTTG
CTTATGGGAAGACTCGAGAACAGTTGCTTGGTGAGAAGATCCCTAACCACCTTATTCCTCACA
AGACTTTTTCTGGCAACCGTCCATCACTAAGTCTACTACTACCCTCATTAAGTGCTTACAACAT
TGGACAGGTATGTGCAGTCTTGATGGATACCTTTTTTGTTGATGCTGTGCGAAAATGTTAG
F: CTACCAGCTAATCCATCAGG
R: AAGACTGCACATACCTGTCC

glgP
2.4.1.1: glycogen phosphorylase
>Cluster-24380.17009;orf1 len=1401 frame:3 start:3 end:1403 gi|502091132|ref|XP_004489452.1| PREDICTED: alpha-1,4 glucan phosphorylase L isozyme, chloroplastic/amyloplastic [*Cicer arietinum*]
GTTGTATCCGAAGAAGTTGAATATGAAGAAGTTGAATCCAAAGAAGTTGAATCCGAAGAAGT
TGAATCTCAAGAAGTTGAATCTGAAGAAGTTGAATCTCAAGAAGTTGAATCTGAAGAAGTTG
AACCAGAAGAAGAGGATCTTGGCAATGAAGAACCAACATTTTTAAAATCAGATCCTAGATTGC
CAAAAATGGTTCGTATGGCTAATCTCTGTGTAGTTGGTGGGCATGCGGTGAATGGAGTTGCTG
AAATTCACAGTGAAATTGTGAAGGAGGATGTATTCAACAGTTTCTACAAGTTGTGGCCTGAGA
AATTTCAAAACAAAACAAATGGGGTAACTCCAAGACGCTGGATCAAATTCTGCAATCCTGATC

TAAGTAACATAATTACTAAGTGGATTGGTACAGATGATTGGGTTCTGAACACTGAGAAACTGG
CAGAACTCAGGAAGCTTGCTGATAATGAGGATCTCCATTTGGAGTGGAGGCAGCTAAAAGAA
GCAATAAGATGAAGGTTGTTTCTTTCATAAAAGAGAAAACAGGATATGTTGTCAGTCCGGATG
CGATGTTTGATATACAGGTGAAGCGGATACATGAATATAAACGTCAGTTGCTGAATATCTTAGG
AATTGTCTACCGCTACAAGAAGATGAAAGAAATGAGTGCCAAGGAAAGGATATCTAGCTTTGT
TCCGAGGGTCTGCATATTTGGGGGTAAAGCATTTGCTACATATGTGCAGGCAAAGAGGATAGT
AAAGTTCATTACAGATGTTGGGGCTACCATTAATCATGATCCTGACATTGGAAACCTATTGAAG
GTTGTCTTTGTTCCCGATTATAATGTCAGTGTGGCAGAGATGCTTATTCCAGCCAGTGAATTATC
TCAGCATATCAGTACTGCTGGGATGGAAGCAAGTGGAACCAGCAATATGAAATTTGCTATGAA
TGGCTGTGTTCTGATTGGAACATTGGATGGTGCAAATGTCGAAATAAGGCAAGAGGTTGGTGA
AGACAACTTTTTCCTCTTTGGTGCCCGAGCTCATGAAATTGCAGGTTTAAGGAAAGAAAGAG
CAGAAGGCAAGTTTGTGCCAGACCCAAGATTTGAGGAGGTCAAGAAATTTGTCCGTGATGGT
GCCTTTGGAAGTTATAACTATGATGAGTTAATTGGATCCTTAGAAGGGAATAAGGGATTTGGTC
GTGCAGATTATTTTCTTGTTGGCAAGGACTTTCCAGATTATCTTGAGTGCCAACAGAAAGTTG
ATGAAGCGTACAGTGACCAAAAGAAATGGACAAAAATGTCAATTTTGAATACAGCTGGCTCC
TACAAATTCAGCAGTGATCGGACAATTCATGAGTATGCGAGGGATATCTGGGATATCAAGCCTG
TTGTGTTGCCATAG
F: CTGAGAAACTGGCAGAACTC
R: ATCCTTTCCTTGGCACTC

BAM
3.2.1.2: beta-amylase
>Cluster-47525.6;orf1 len=612 frame:-1 start:3061 end:2450 gi|743771415|ref|XP_010915994.1|
PREDICTED: beta-amylase 1, chloroplastic-like [*Elaeis guineensis*]
CCTCCCTCCCCCCCCCCCCCCCCACCCCCCCACCACTCTCTCTCCAACTCCTCCGCTCGAATG
GCGGCGATCTCCGCTCCGCCGCCGTCGAGCGTCGCCGCCGCTACCCTCGGCCGCTCCCGCCCC
TTCCCTTCCCGCCTCGCCCTTCCCCTCCTGATCTCGGGGCCCCGCCGACTCCCGCTCCGGCTC
GCCGTCTCGTCTCGCCTCTACTCCTCCAAGCCCTCCTCCGGCGCCAACGGATCCCTCGATAATC
CCTCTTCCTCCGGCGGCGGCGATGAGCTCCACCACGCCCTCCCCCTGCCACCGCGCTCCGAG
AAGGGCGCGCCGGTTTTTGTCACGCTCCCCATGAACGCCGTCGGGGCGACGGGACAGATGGC
CCGGCGGAAGACGATGGGAGCGTCGTTCATGGCCCTCGCGGCCGCCGGCGTGGAGGGGATC
GCGGTGGAGTGCTGGTGGGGGATCGTGGAGAGGGAGGCACCCGGCGTGTACGATTGGGGCG
GGTACATGGAATTGGTGTCCTTGGCGCGCCGTTTCGGCCTCAAGGTGCGGGCGATCATGGCAT
TCCACCAGTGCGGGACGGGCCCTGGTGATCCTTGCTGGTTTGTTGCCTGA
F: CACCACTCTCTCTCCAACTC
R: GAGGAAGAGGGATTATCGAG

GDE
2.4.1.25: glycogen debranching enzyme
>Cluster-24380.67714;orf1 len=2907 frame:3 start:147 end:3053 gi|672144566|ref|XP_008796184.1|
PREDICTED: 4-alpha-glucanotransferase DPE2 isoform X2 [*Phoenix dactylifera*]
GAAGACATGGTGAATTTGGGGTCATATTCTGGAAAGAAGTCCTTGAGTACAGTGACTTTGCTC
TTCAAAATACCATATTATACCCAGTGGGGGCAGAGCCTGCTTATTTCTGGCTCTGAAGCAGTGC
TAGGATCTTGGAATGTGAAGAAGGGTCTGGTTTTAAGTCCATTTCATCAAGGCAATGAGCTTAT
CTGGTGTGGAAGAACTTCTGTCCCCATTGCTTTTACATGCGAATACAGTTACTATTTGGTGGAT
GATGATCGGAATATTTTGAGATGGGAGGGAGGGAAGAAGCGGAACTTAATCCTGCCTGAAGG
ACTTCTGGAAGGAGAGGTAGTGGAAATTCATGATCTGTGGCAGAATGCTTCAGAAGCTCTCTT
CCTTAGAAGTGCTTTCAAAAATGTCATCTTTGGTGGAGATAAGAACTTGGAAGCAGAAACATA
TTCTGGGGCTCTTCAGAAAAACCTGGAACGGAAAGATTCCATTGTTGTCCAATTTGTGATTAG
CTGCCCATATTTAGAAGTTGGATCATCTGTTTGTGTTACTGGTAGTGTGCCACAATTAGGACAG

TGGAAGGCTGAAGATGGACTGGAACTTAGCCATGCAGGAGGATCTATATGGAGAGCAGATTG
TTTAATGCGAAAAGATGACTTCCCAATAAAATACAAGTATTTCCGAGTTAGCAAAGTTCAAGG
TGCTTCTTTGGAAGTTGGCCCTAACAGGGAGCTAGTTGTTGAGTTGGCATCAAAATCTCCACC
AAATTATATTATATTATCTGATGGTACCTTCCGGGAAATGCCATGGAGGGGTGCTGGTGTTGCC
ATACCAATGTTCTCTGTTAGGTCCAACGATGACCTTGGAGTTGGAGAGTTTCTCGATCTAAAAT
TACTTGTTGATTTGGCTGTGGATTCAGGCTTTCATCTTGTTCAACTCTTGCCAGTAAATGATACT
TCAGTCCATGGGATGTGGTGGGACTCATATCCCTACAGCTCTCTTTCTGTATTTGCATTGCATCC
CCTGTATCTGAGAGTACAAGCACTTTCGGAAAATATTCCAGAAGAGATAAAGCAAGAGATCTC
GAGGGAAAAAGAAAGGCTGAATCAAAAGAATGTTGATTATGAGGCTACAATGACTGCTAAAT
TATCAATTGCAAAAAAAGTATTCAATCTGGAAAAAGATAAAATTCTTGATTCCAGCTCCTTCAA
GAAATTCTTCTCTGAGAATGAGGAATGGCTGAAACCCTATGCAGCATTTTGTTTTTTGAGGGA
CTTCTTTGAGACATCAGATCATACCCAATGGGGTCGATTTTCTCATTTTTCCAAAGAAAAGCTT
GAGAAACTTGTTTCAGAGGACAGTTTACACTATGATGTCATATGCTTTCATTACTATGTTCAGTT
TCATCTGCATTTACAATTATCAGAAGCAGCAGCCTATGCGAGGAAGAAGAAAGTAGTCTTGAA
AGGAGATTTACCAATAGGTGTGGACAGGAATAGTGTGGATACTTGGGTATATCCTAATTTATTT
CGTATGAACACATCTACTGGAGCACCTCCAGATTATTTTGACAAAAATGGACAAAATTGGGGT
TTCCCCACATACAACTGGGAGGAAATGTCAAAAGATAACTATGCATGGTGGCGAGCCCGATTG
TCACAGATGGCAAAATATTTCACTGCCTATAGAATAGATCATATCTTGGGTTTCTTTAGAATATG
GGAGCTTCCTGATCATGCTGTTACTGGTTTACTTGGAAAATTCCGTCCATCCATTGCTTTGAGC
CAGGAGGAACTTGAGAGGGAAGGCATTTGGGATTTCAATCGCTTGAGCCGTCCATATATCCGG
CAAGAAATTTTACAGGATAAATTTGGATCCTTGTGGACTGTTATTGCTGCAAACTTTTTGAATG
AATATCAAAAGCTATGCTATGAGTTCAAAGAAGACTGCAATACAGAGAAGAAAATTATTGCGA
AGCTTAAATCCAGTCCTGAGAAGTTATTGTGGTTGGAAAAAGAAGACAAGATACGGAAAGAC
CTTTTTGATCTTCTACAGAATATAGTTCTAATTAGAGATCCTGAAGACTCAAGAAAATTTTATCC
TCGTTTCAATCTTGAAGATACCTCCAGTTTTAAAGATTTAGATGAACACAGTAAAAATGTTCTC
AAAAGATTGTACTATGATTACTATTTCTGCCGGCAAGAAGCTCTTTGGCGTCAAAATGCACTG
AAGACTCTGCCTGTCCTATTGAACTCATCAGATATGTTGGCTTGTGGAGAGGATCTTGGCCTTA
TTCCTTCCTGTGTCCATCCAGTCATGCAGGAACTAGGGTTAATAGGTTTACGTATTCAAAGAAT
GCCAAGTGAGCCAGGCCTAGAATTTGGTATTCCATCTCAGTACAGCTATATGACGGTATGTGCA
CCATCATGCCATGACTGCTCCACTTTGCGTGCTTGGTGGGAAGAAGATGAAGAGAGAAGCTG
CCGTTATTACAAGACTGTGGTTGGATGCAATGATGTGCCCCCTTCTTGTTGCACCCCAGAAATA
GCATACTTCATCATTCAGCAGCACTGTCAAGCTCCATCAATGTGGGCAATCTTCCCACTTCAGG
ACTTGCTGGCACTGAAAGAGGAATACAGAAGTAGACCAGCAGTAGAGGAAACAATCAATGAT
CCCACAAACCCAAAGCATTATTGGCGATACCGTGTACACGTGACATTAGAGTCCTTACTGGGT
GACGAGGATCTAAAGACAACCATAAAAGATCTTGTGAGAAGTAGTGGGAGGTCATTTCCTGT
GACTGTAGGGTCTGACGTGCAAGAGAACCAAAACGATATTGCAGGCTTGGTGAAGAAGCAA
ATTAAAAATGAGCAGGAAAAGATTTCTATGGTCCATTTAAATGATCATTCATAG
F: AAGGACTTCTGGAAGGAGAG
R: GGCACACTACCAGTAACACA

GlgB

2.4.1.18: 1,4-alpha-glucan branching enzyme

>Cluster-24380.71026;orf1 len=2541 frame:-3 start:3073 end:533 gi|672121366|ref|XP_008783983.1| PREDICTED: 1,4-alpha-glucan-branching enzyme 1, chloroplastic/amyloplastic [*Phoenix dactylifera*]
TCGATGGCCTTCGCGTTCGCGGGGATTCGCTTCACCGCTGCGCAAGCTCCGGGGCTATCACTT
CGCTCTGTGATGCGTGGCGATCGCAAGAATGGCGGAAATGCATCTCTCGCCTTCTTGAAGAAG
GACTCTTTCCCTTGGAAACTACTTGCTGGAAATTCTTCTTATGAACCTGATTCTGCATCCACAA
CTGTTGCTGCATCTGGAAAGGTGCTGCTTCCAGGTGGCGAAAGTGATGGCTTGTCATCTTCTA
CAGATTCAATTGGGAACCCTGAAGTTTCTCCTGATGACCTGCAGGTATTACAAGAATCAACAG
GTCTATCCATTAAAGATGATGGTAAGGTAGAAGCTGGGCAAACACCCATGTCTTCAGAAGTCA
TGGATGATGAAATCATGAATGAAGCTGCTAAACAGTCTGTACATTCTCAAGCCAATCAAACTAT

TGAAAAAGTTGAAGAGAAATCAAGATTTATTCCTCCACCTGGTGGTGGACAAAGAATTTATGA
AGTTGACCCATATTTGGAAGGCCATCGTGAACATCTTGATTATCGATATAGCCAGTACAAGAAT
ATGCGAGGGCTGATTGATCAGTATGAAGGTGGCTTGGATGCATTCTCCCGTGGCTATGAGAAA
TTTGGTTTTGTCCGCAGTGCTGATGGTGTTACTTATCGGGAGTGGGCCCCTGGAGCAAAGTCA
GCAGCACTCATTGGAGACTTTAACAATTGGAATCCCAATGCAGATGTCATGAATCGGAATGAG
TTTGGTGTATGGGAGATTTTTCTGCCCAATAATGCTGATGGCTCACCCCCTATACCCCATGGTTC
TCGTGTCAAGATACACATGGATACTCCATCCGGCATTAAAGATTCGATTCCTGCATGGATAAAG
TTTTCTGTACAGGCCCCAGGTGAAATACCATACAATGGAATATATTATGATCCCCCTGAGGAGG
AAAAATATGTTTTTCAACATCCTCAACCAAAAGCTCCAAAGTCATTGCGCATTTATGAATCACA
TGTTGGGATGAGTAGTCCAGAGCCCAAGATTAACACATATGTTAGCTTTAGGGATGATGTGCTG
CCTCGAATCAAAAGGCTTGGATACAATGCTGTTCAGATAATGGCCATTCAGGAGCATTCATATT
ATGCGAGCTTTGGGTATCATGTTACTAATTTTTTTGCACCTAGTAGCCGTTTTGGAACGCCAGA
TGAACTCAAGTCTTTGATCGATAGAGCTCATGAGCTTGGTCTGCTTGTTCTCATGGATATTGTT
CACAGCCATGCATCAAATAACGTCCTGGATGGGTTAAATCAGTTTGATGGAACTGACACACAT
TACTTCCATTCTGGTTCACGGGGATATCATTGGATGTGGGATTCTCGCCTTTTCAACTATGGAA
GTTGGGAAGTGCTAAGGTTTCTACTATCAAATGCAAGATGGTGGCTAGAGGAGTACAAGTTTG
ATGGGTTCAGATTTGATGGTGTAACATCAATGATGTATACTCACCATGGGTTACAAGTAGCATT
TACTGGGAATTACAATGAGTACTTTGGATATGCTACTGATGTGGATGCAATGATCTATCTGATGC
TGGTAAATGATATGATTCATGGACTTTATCCTAAGGCTGTTACCATTGGTGAAGATGTCAGTGG
AATGCCAGCATTCTGCATCCCTGTTCAAGATGGTGGTGTAGGATTTGATTATCGCCTTCATATGG
CTATTGCTGATAAATGGATTGAACTACTCAAGTTAAATGATGAGCATTGGAAAATGGGAGACAT
TGTTCACACTCTCACAAATAGGAGATGGTTAGAGAAATGTGTTGCTTATGCAGAAAGCCATGA
TCAAGCTCTTGTTGGTGACAAGACTATTGCATTCTGGCTAATGGACAAGGATATGTATGATTTT
ATGGCTCTGGATAGACCATCAACACCCCGGATAGATCGTGGAATAGCTCTACACAAGATGATTA
GACTGATCACGATGGGATTAGGTGGAGAAGGATATCTTAATTTTATGGGAAATGAATTTGGGCA
TCCAGAATGGATAGATTTTCCTAGAGGTGACCAACATCTTGCTGGTGGAGTTGTGATACCAGG
GAATAATAATAGTTTTGATAAATGCCGCCGTATGTTTGATATAGGAGATGCAGATTATCTCAGAT
ATCGTGGAATGCAAGAATTTGATCAAGCAATGCAGCATCTTGAAGAGAAATATGGTTTCATGA
CATCAGAGCATCAATATATATCACGAAAAGATGAAGGGGATAGGGTGATTGTATTTGAGAGAG
GAGATTTGCTGTTTGTTTTCAATTTTCATTGGACTAATAGCTACTTTGACTACCGAGTTGGTTGC
CTAAAGCCTGGAAAATACAAGGTGGTTTTAGATTCTGATGACAAACTTTTTGGTGGATTCAAC
CGAATCGATCACACTGCAGAATACTTCAGCAATGAAGGTTTTTATGGTAATAGGCCCCGTTCAT
TCTCGGTATATGCCCCAAGTAGAACAGCTGTCGTGTATGCTCTGTTTGAAGACTGA
F: ATGGTGGCTAGAGGAGTACA
R: CCTACACCACCATCTTGAAC

AUX1: auxin influx carrier

>Cluster-42569.10;orf1 len=1470 frame:-2 start:1699 end:230
gi|672143158|ref|XP_008795448.1| PREDICTED: auxin transporter-like protein 2 [Phoenix dactylifera]>gi|672143160|ref|XP_008795449.1| PREDICTED: auxin transporter-like protein 2 [*Phoenix dactylifera*]
CAGAGCAAGGACGAAAAGATGCTGTCGCAGAAGCAAGCGGAGGAGGCCATCGTGGCCAGCT
TCAACGAGACGGAGCAGGACGGGAACGGCAAGGAGGAGGAGGAGGAGAGGGCGGAGGAG
CCGCAGCCCATGTTCAGCATGAAGAGCCTCCTCTGGCACGGTGGCTCCGTCTGGGACGCCTG
GTTCAGCTGCGCCTCCAATCAAGTGGCCCAGGTTCTGTTGACGCTGCCCTACTCGTTCTCGCA
GCTGGGGATGCTGTCCGGGGTGCTGCTCCAGCTGTTCTACGGTTTCATGGGCAGCTGGACGGC
CTACCTCATCAGCGTTCTCTACGTCGAGTACCGCACCCGCAAGGAAAAGGAGAACGTCAGCT
TCAAGAATCACGTCATCCAGTGGTTCGAGGTGCTGGATGGGCTGCTGGGCCCTTACTGGAAA
GCCATCGGTCTGGCCTTCAACTGTACCTTCCTCCTCTTCGGCTCCGTTATCCAGCTGATCGCTT

GTGCCAGTAACATTTACTACATCAACGACCGGCTGGACAAGAGGACGTGGACGTACATATTCG
GCGCCTGCTGCGCAACCACGGTGTTCATCCCTTCGTTCCACAACTACCGGATATGGTCCTTCCT
TGGTCTGGGCATGACCACCTACACCGCCTGGTACCTCACCGTCGCCGCTCTCGTCAACGGCCA
GGTTGAAGGCGTGGCGCACTCGGCTCCCGCAAAGCTGGTCCTCTACTTCACCGGCGCCACCA
ACATACTCTACACTTTCGGCGGCCACGCCGTCACCGTGGAGATCATGCACGCGATGTGGAAGC
CCCAGAAGTTCAAGTACATATACCTGGTGGCGACGCTATACGTGTTCACGCTGACGCTACCGT
CGGCAGCGGCCATGTACTGGGCATTCGGGGACCAGCTGCTGACTCACTCCAACGCCTTCTCTC
TGCTGCCCAAGTCGCGGTTCAGGGACGCCGCGGTGATCCTGATGCTGATCCACCAGTTCATCA
CCTTCGGATTTGCGTGCACGCCCCTCTACTTCGTGTGGGAGAAGGTGATCGGCATGCATGACA
CCAAGAGCATCTGCCTGCGCGCGCTGGCCCGTCTCCCCGTGGTTATCCCCATCTGGTTCCTCG
CCATCATCTTCCCCTTCTTCGGGCCCATCAACTCTACCGTCGGGGCCCTCCTCGTTAGCTTCAC
CGTCTATATCATCCCTTCCCTCGCCCACATGCTCACCTACCGCAAGGCCTCTGCCAGACAGAAC
GCGGCGGAGAAGCCACCCTTCTTCATGCCTAGCTGGACCGCCATGTACGTGTTGAACGCGTTC
GTGGTGGTGTGGGTGCTGGTGGTGGGGTTCGGGCTCGGCGGGTGGGCCAGCATGGTCAATTT
CGTCAGACAGGTGGACACCTTTGGGCTCTTCGCCAAGTGCTACCAGTGCCCCAAGCCCCCCG
TCGCGGCGCCCCCACCACAGCAGCACTGA
F: CACAACTACCGGATATGGTC
R: GTCAGCGTGAACACGTATAG

TIR1: transport inhibitor response 1
>Cluster-24380.25329;orf1 len=573 frame:-2 start:575 end:3 gi|743856218|ref|XP_010941793.1|
PREDICTED: transport inhibitor response 1-like protein Os05g0150500 [*Elaeis guineensis*]
CTCCCGACGCCGCCGATCCGACGCCCGATCATGCCCGCCGGTGGACGGTGGGCCGTCATGTTC
CCTGACGAGGTCTGGGAGCACATCTTCTCCTTCGTCGCTGACGACCGCGACCGCAACGCCGT
CTCCCTCGTCTGTAGCGGCTGGTACCGTATCGAGCGCCGCTCCCGCCGCCGGATCTTCGTTGG
CAACTGCTACGCTATCTCCCCCGCCGCCGCCGTCCGCCGCTTCCCCGAGGTCCGGGCCGTCTC
CATCAAGGGCAAGCCCCACTTCGCTGACTTCAACCTCGTACCCAGCGACTGGGGCGGTGGGG
CTGAGGCTTGGGTCGCCGCCATGGCCGAAGGGTGGCCGCAGCTCGAGGAGCTCCGCCTCAA
GCGGATGGTCGTCACCGACGACTGCTTCGAGCTCATTGCCCGATCGTTCAAGAACTTTAGAGT
CCTCGCGCTTTCCTCCTGCGAGGGGTTCAGCACCGCCGGCCTCGCCGCAATCGCCGCCAATTG
CAGAAATCTAAGGGAGATTGACTTACATGAAAATGAAGTAGAGGATAATTCTGTGCACTGGCT
CAGCCATTTT
F: CAACTGCTACGCTATCTCC
R: CAATGAGCTCGAAGCAGT

AUXIAA: auxin-responsive protein IAA
>Cluster-24380.68446;orf1 len=1029 frame:1 start:511 end:1539 gi|743756123|ref|XP_010915232.1|
PREDICTED: auxin-responsive protein IAA6-like isoform X1 [*Elaeis guineensis*]
GAAGGACTCCAAAGAAGAAGTCATATGGAAAAAGATTATAAAAACAAACCAGATGCCTGTCC
TCAGCTCCTTGATTTGATTCCAAAGGAGAGAGACTGGATGGTGAGAGTCTCGGGAGGAGGAG
GATGTGGAAGTGGTTCAAAAGTTTCCGAGGAGAGAAAGCTAGAGCTGAGGCTTGGTCCTCCT
GGAGGAGAAGAGGAGTCCTCTGTTCTATCTCTTGGATACTTTTCCAAGGCCTCAAAGACTACC
AATCCGTGTGCTGGAGCCAAAAGGGGATTTTTAGACACAGTTGAGTCCAAAACAGAAGATCC
TCATCAGCAGCAGAGTGGGTTCTTTCAGCTACATAGCAGAGGTGGCTTGGGACAGGAATTATC
ACAGAGAACAAATCGCAACGTAGAGCAGCAGCCGCAGCAGCAGAGCCTTGAGAGGAAGGC
ATGCAGTTCATTACCACCAGTACATGCTGCTCCTGGGAGAAAAGCTGGAGCGAACAACACCT
CTCAAACAAGAAACAGAACTACCTCTCCGGCTGTGGTTGGGTGGCCTCCTATCCGTTCGTTCA
GGAAAAATCTGGCAAGCAGCTCTTCTAAACCAACATTTCAATCACAGAATGGGAACTCAGAG
ACTAGACCAAAGCTTGAGAACTGCAGGAAGGGTTTATTTGTGAAAATTAACATGGATGGGATC
CCGATAGGAAGGAAAATAGATTTGAAGGCCTATGATGGCTACGAGAAGCTCTCTTCAGCTGTA
GAAGAGCTCTTCCAAGGCCTTCTTTCAGCTCAAAGGGATCCTTCTACAGCTGAGACCCAGAA

GGATGCAGAAGAAAGAAAAGCATTTACAGGCTTATTAGATGGGACTGGTGAATATACCTTGGT
TTATGAAGACAATGAAGGAGACAGAATGCTGGTTGGGGATGTTCCTTGGGACATGTTTGTCTC
TGCTGTTAAGAGGTTGAGGGTGCTGAAGAGTTCCGATCTTTCTGTGCTATCTTTGGGAGCAGT
CTGCCGGAAAAGAACTGCGACAGAGTGTTGA
F: CCTATGATGGCTACGAGAAG
R: CAGACTGCTCCCAAAGATAG

ARF: auxin response factor
>Cluster-24380.18916;orf1 len=294 frame:-3 start:294 end:1 gi|743756901|ref|XP_010919282.1|
PREDICTED: auxin response factor 15-like isoform X2 [*Elaeis guineensis*]
ATACTGCAGGTTCACAAGCAAGGAAGTGTTGTAGGCAGGGTGGTTGATCTCTCAAAACTTGA
TGGATATGATGATCTGATTTGTGAGCTTGAACGGCTGTTTGATATGAAAGGGCTGTTAAATGAC
CCACAGAAAAAGTGGAATGTTGTCTATACCGATGATGAAGATGATATGATGCTTCTTGGGGATG
ATCCTTGGCAGTTGAGTGCTAGCACCTGCCTCTGTTATCTGAACTATTATACATTTGTTGTACTG
CTGCCTGACACTGAAGTTAGCATTTTCATTTCTCTGCAG
F: GCAAGGAAGTGTTGTAGGC
R: GATAACAGAGGCAGGTGCTA

CRE1: arabidopsis histidine kinase 2/3/4 (cytokinin receptor)
>Cluster-24380.14000;orf1 len=3075 frame:3 start:1362 end:4436 gi|743806685|ref|XP_010927761.1|
PREDICTED: histidine kinase 3-like isoform X1 [*Elaeis guineensis*]
GTACTCTGCATGCATTTGGGCTTGGATTTGGTGTTACTGTTTTTGATGGTATGTTGGTGGCTATT
CTCTGAGATCCCCATGACTTGTATCATTTATGCTGATATAATGGAGCGGAAGGCTGCTTTCCTTG
GTGATAAGAGCAAGCTCTGGTTGGGAGACAAATTCCTGCCCCGAGGCTGGAAGAGTCATCTT
TATAACCACTACTTTGGGTCGAAGAAGGTCAGAGAAACATGGTGGAGGAAGCTTCTGATTTTG
TGGGTGGTTGGTTGGTTCTTAGTGTCTCTGTGGATCTTCTGGTTGATGAACTCCCAGGCTGTCG
AGAAGAGTAGGGAGACGCTTGCTAGCATGTGCGACGAGCGTGCTCGGATGCTTCAGGACCAG
TTCAATGTGAGCATGAACCATCTTCAGGCGTTGGCAATTCTTGTCTCAACGTTTCATCATTCCA
AGGAGCCATCTGCCATTGATCAGATGACTTTTGCAAGATATGCAGAAAGAACTGCTTTTGAGA
GGCCGCTGACGAGCGGTGTGGCTTATGCTGTGAAGGTTCTGCATTCTGAAAGAGAAGAGTTT
GAGAGGCAACAAGGATGGACTATCAAGAGAATGGACTGCACAGAGCAATCCCCAGCTCGTAA
TGATGACTCGTTCCCTGAGACCCAGGAGATATCACCAGCGCAAGATGAATATGCTCCTGTCAT
CTTTGCCCAAGATACCTATAAGCATGTGATATCTGTGGATATGTTATCTGGAAAGGAGGATCGT
GAAAACATTCTACGAGCTAGGAAATCTGGGAAGGGAGTCCTAACTGCTCCTTTTCAGCTGCTA
AAATCAAAACGCCTTGGGGTTATTCTGACATATGCAGTGTACAAAAGAGAAATTCCTTCAAAT
GCAACACCTGTGGAACGTATCCAAGCAGCCATAGGATACTTAGGAGGAATCTTTGATATAGAA
GCGCTGGTAGATAAATTACTTCACCAACTTGCTAGTAAGCAATCCATTATTGTCAATGTGTATG
ATACCACCAATCCTGACGAACCAATCAGGATGTATGGTCCAAACATGACCGGTACTGGTATTTA
CCATAACAGCACCCTTCACTTTGGCGATCCATTAAGAAAGCATGAGATGCATTGCAGGTTCAA
ATACAAGCCACCATTGCCATGGCTTGCCATAACAACATCAATCGGAGCTCTTGTGATTGCCTTA
CTAATTGGATATATTTTTCATGCTACTGTCAACCGCATTGCTAAAGTGGAAGATGATTACCGGG
AGATGATGGAACTCAAAAAACGTGCAGAAGCAGCAGATGTTGCAAAATCTCAGTTCTTGGCC
ACTGTATCTCATGAAATCAGAACTCCGATGAATGGAGTTTTAGGGATGCTGCAAATGCTTATGG
ATACTGATCTGGATATCACACAGCAAGATTATGTGAGAACAGCCCAAGAAAGTGGAAAAGCT
CTTGTATCACTTATAAATGAGGTTCTGGATCAGGCAAAGATTGAATCCGGCAAGCTTGAGCTT
GAGGCTGTCAGATTTGACTTGCGAGCAGTTCTGGATGATGTCTTGTCACTTTTCTATGGAAAG
TCCCAGGAAAAAGGAATAGAGTTGGCAGTTTTTGTTTCTGATCAAGTTCCCGAATTTCTTGTC
GGTGATTCTGGAAGAATCCGGCAAATCATCACAAATCTCATGGGAAATTCCATAAAATTCACA
GAGAAAGGGCATATTTATGTGACTGTTCATCTTGTCGAAGAGATGATGAATTCATTGGAAGTG
GAAACAGAAGCTCAGCCGGTGAATACCTTGAGTGGTTTTCCTGTGGTGGATAGAAGACGCAG
CTGGGAGAGCTTTAAGATATTCAATCAGGATTTGTCTATGACTGAGCTGTCTTTTCTGTCAACC

TCTTCGGATCCTATTAACCTAATCATATCTGTTGAAGATACAGGGGTGGGAATCCCTCAAGAAG
CCCAGTGTCGGGTGTTCACCCCTTTTATGCAGGTAGGCCCATCAATTTCACGCATTCATGGGGG
CACTGGCATTGGACTTAGCATCAGTAAATGTTTGGTTAGTCTCATGAAGGGAGAGATTGGATTT
GTGAGTGAACCCCAGATCGGTTCCACTTTCACCTTTACTGCTATCCTTACGAGAACATGCAAC
AATTCAAACGAGTACAAATCATCCGAATTTCAAGGGATGACTGCATTAGTGGTCGACCACAGA
CCGGCCCGTGCAAAGGTCACAAGGTACCATCTCCGGAGGCTTGGTATTCATGCTGAACTAGCC
AGTGACCTAAACCAAGTTCTTCCCAGAATAACTAATGGAACATTAATGGTAAACATGGTTCTT
GTCGACAAGGAAACATGGCTGAAGGATGCTAATCTCTGGCCTCTTTTTATTAGCAAATTAAGG
AAGGATGACCAGACAAATATTCCAAAACTTTTTCTATTAACAAATCCTAGCAGTTCTCCCAAA
AACAGTCCCGCTAGCTCTGCAGAATATTTTTCAACTACAATCATGAAGCCACTCAGAGCGAGC
ATGTTACAAGTATCTCTGCACCGAGCTATGGGTGGTGGAGACCGGGACAATTGCCGAAATGGA
GGAGTGCCTCGATTGTCATTGCACAATCTTCTTCATGGGAAGCATATTCTGGTTGTGGATGATA
ATATCGTGAATCTAAGAGTAGCTGCCGGTGCTTTGAAGAAGTATGGAGCTGAAGTAACTTGTG
CAGAGAGTGGGAAGAAGGCAATAGCAATGCTCAAGCCACCACACAAATTTGATGCTTGTTTC
ATGGATATTCAGATGCCAGAAATGGATGGATTTGAAGCTACCAAAAGGATACGAGAGATGGAA
AATGATCTGAATGATCGAATACAACATGGGGAAGTGTCACTAGAAGCTTATGGAAATGTCTTG
CATTGGCAGACTCCCATATTGGCCATGACGGCAGATGTGATCCAGGCAACACAGGAGGAGTG
CCTAAGGTGTGGAATGGATGGCTATGTATCAAAGCCATTTGAAGGAGAACAACTGTACAGAG
AAGTAGCTCGCTTTTTCAAAACCACCATGAAGAAAACTCAATAG
F: GAGCAGTTCTGGATGATGTC
R: GTCTTCTATCCACCACAGGA

B-ARR: two-component response regulator ARR-B family
>Cluster-24380.70438;orf1 len=2121 frame:1 start:373 end:2493 gi|743774335|ref|XP_010917519.1|
PREDICTED: two-component response regulator ARR1-like [*Elaeis guineensis*]
GGTGGATTTGAGGTGGTAGGTTTTGGTGTGTCTGAAGGGAGATTTGAAGTGATGGCTACTGTT
CAGAAGTTGCCGGGGTCGAGCACGAGCACGGCGAGCAGCTACGGTTCTTGCAGGGTGGATG
ATGAGCAGTTCCCTGCGGGGCTGAGGGTTCTTGTGGTGGATGATGATGTGACTTGCTTGAGGA
TTTTGGAGCACATGCTGCAGAGGTGCCGATACCATGTTACAACTTGCTGTCAGGCTACACAAG
CTTTATCCCTTCTTAGAGAAAGGAAGGGTGGTTATGATGTTGTGATAAGTGATGTTCATATGCC
TGACATGGATGGATATAAGCTACTTGAACTTGTTGGACTTGAAATGGATCTTCCTGTTATTATGA
TGTCTGCTGATGGGAGAACTAGTGCGGTGATGAGAGGGATAAAGCATGGGGCATGTGATTATT
TGATTAAGCCTGTACGCATAGAAGAGTTGAAGAACATATGGCAGCATGTTATTAGAAAAAAAT
GGAACGAAAATAGAGATCTTGAACATTCAGGTACCATGGAGGAATCTGATCGTTTTAGACGTG
TTGTTGATGATACTGAGTATGCTTCATCAGTGAATGATGGAACAGATGGTACTTGGAAATCCCA
GAAAAAGAAAAGGGATACTAAAGAAGATGAAGATGATGGTGAACTGGACAATGAGGATCCTT
CTGCATCAAAGAAGCCACGTGTGGTATGGTCAGTGGAGCTCCATCAGCAATTTGTCAGTGCTG
TGAATCAACTTGGCATTGACAAGGCTGTCCCTAAGAGAATTCTAGAATTGATGAATGTTCCTG
GCCTGACTAGGGAAAATGTTGCAAGCCATTTGCAGAAATTCAGACTATACTTGAAGAGGTTAA
GTGGGGTTGCTCAGAACCAGAGTGTGCTTCCTAATTCATTCTGTGGATCTGTAGAACCAAATG
CCAAATTGGGCTCATTGGGCAGATTTGATTTCCAAGCTTTGGCTGCTTCAGGTCAAATTCCAC
CACAAACCCTAGCTGCCTTGCATGTTGAGCTCTTAGGCCGACCCTCAGGCAGCTTAGTGTTGC
CAGCAGTTGATCAACCAGTTCTTCTACATGCTTCGATGCGAGGACCTAAGTGCATTCCTGTTG
AACAAGGAGTGGCATTTGGTCAACCTCTATTGAAATGCCAATCCAGTACACAGAAGCAGCTTC
CTCAGTCCAACATTGCTGTTGAAGACATGTCTTCTGGTTTTTCAGCTTGGTCTGGTAATCAATT
CAGTCCAACAGGTACTGCTGGCAATCTTGGAGGGGTGAACAAAAGTCAGAGTGGTAACTTGC
TGGTGCAAATGTTACAACAGCAACACCCCTATCCTGTGCTATCAGAATCTAATCATGCAATTAA
CGTACAACCTTCTTGTCTTATAGCTCCCTCACAGTCATCAAATGGTTTCCAGATAGGGAACAGT
AATGTTCCAATAAATCGGAACTCTACAGGGGATACCTCTCTGTCATCCACAAGCTTTCAGGTC
AGGAGCAATGCTCTTCCTGTTAATCAAAATTCTAACTATACTAACAACAGCACAGTCATGGATT
GCAGCCTGATATCTTCTCAGTCACATAATGTACCATTGGATGTGGAACAAGTCCCAGATGCAG

ACTTAAAGAATGTCAGTGTGCTAAATGGGTACTCAGTTCCAGGATCAGCTTCGCCATCAGTGT
CATCCTGCTCAGTTCGTCCAGAAAGCTGCACTGGTTGGAAGGTGCAGAACTTGGTAGTAAAT
GTTGCTCCGGCAAGTAGATTGCCCGCTCACTTGCCTAGCTTGTGCGTTCAGGGATCTGACCCA
AAAATGATCGCATTGCCTGACCAAGGACGAGGCAGGAATCTTGGATTTGTGGGTAAAGGTAC
ATGCATCCCCAGTCGGTTTGCCGTGGATGACAATGAATCTCCAACTAATGACCCAAGTCATTG
GAATATGTGCATTGGTAATGAAAGGGTCAGAGTGAAGCAAGAGACTGTTTTGGATTTTGGAG
ATGGTTTTGAAGGCGAGCATGCTAAGTTACCACATTTCTCTCCAAGTGATCTCATGGGTGTTCT
GTCCAAGGATGGGTTGGCAGGATGCTACCTGAAATCCTGA
F: AGGGGATACCTCTCTGTCAT
R: CTACCAAGTTCTGCACCTTC

PYR/PYL: abscisic acid receptor PYR/PYL family
>Cluster-24380.59206;orf1 len=708 frame:1 start:7 end:714 gi|743890673|ref|XP_010911141.1|
PREDICTED: abscisic acid receptor PYR1-like [*Elaeis guineensis*]
ACGGGATGCGCTTCTCTCCAGCCCCGGGGGAGGAGGAAGAAGACGAAGCCATCCATGGAGG
GAGGAGGATCGGAACAGGAGTCTTCGAGATCGCCGGCGGTGGAGGGGGAAATTCTGGCGGG
GCTCACGGCGGAGGAGCAGGAGGAACTCCGGACGACGATCGAGGCGCACCACCGGTACCGG
GTGGGGCCGGGCCAATGCTCCTCGCTGCTCACGCAGCGGATCCGCGCCCCGGCCGCGACCAT
CTGGTCGGTGGTCCGCCGCTTCGACCGTCCTCAGATCTACAAGCACTTCATCCGCAGCTGCGC
CATCAAGGGCGGAGGCGAGGTCCGCGTGGGCTGCCTCCGCGAGGTCTGCGTCATCTCCGGCC
TCCCGGCGAGCACCAGCACCGAGCGGCTCGACGTGCTCGACGAGGACCGGAGGGTCGTCGG
GTTCAGCATCATCGGTGGGGAGCACCGCCTCCAGAACTACCGCTCCGTGACGACGGTCACCG
AGCTCGGCCCGCCGGGGAAAGGGATCTGGACAGTGGTGCTGGAATCCTACGTCGTGGACGTG
CCGGAGGGGAACACCGTTGACGACACCCGGCTCTTCGCCGACACCGTTGTCCGCCTCAACCT
GCAGAAGCTCGCCTCGGTTACTGAGGCGATGGCGGCCGCGGCCGCCGCCACCGCCACCGCCG
CCAATCCGGAAAAGGGTAAGTCACCGTAG
F: GACCGTCCTCAGATCTACAA
R: GTAGGATTCCAGCACCACT

PP2C: protein phosphatase 2C
>Cluster-24380.23154;orf1 len=1074 frame:-1 start:1074 end:1 gi|743797665|ref|XP_010925040.1|
PREDICTED: probable protein phosphatase 2C 6 isoform X2 [*Elaeis guineensis*]
ACTCAAAGGGAGGAGTCGATGGAGGACATGTCTCCGGCTGTTGCTTTGCCATTTAGGACCGG
CAATTCGCACCGCGAAGAGGTCGCTTGCCTTCAGCTGATAACGGACCCTGCCGCGACCCTGC
TGTCCGATACGATGGCTGACCATGTGCCGGCCGAGCGAGAGGAAGCTGCCCAAGTAGTGGTG
ATGGAGGAGGGAGGAGATGGGGATGAGCTCGAGGACCGGACGGTACACGGGAGCGAAGAA
GAGGATTCCGTTTCAGTCGTACCGGAGCCATCGGTTGGGAGCTCGATCAGCGATAGCAGCAG
CAGCATCGCCAGTGCCACTGACGAGTTCTCGAGCTTGGACCCGTCTTCCGAGGCCGGGACGC
CGATCTCTGTGGCGGGACCTGCTTTGACTGCGAATGTGGGTGTGCTGCCGGCGGTCGCCGAA
CCAGAGGTTCAGGGAGGTCGGGCCAGTGCAAGTGGCGGTGGGCGGAGCGTGTTCTTGACGA
ATTACGTACCGCTCTGGGGCTGCGTATCCATTTGTGGGCGGAGGCCGGAGATGGAGGATGCTG
TGGTGGCGGTGCCTGGATTCTTCGAAATCCCGCTCCGGATGCTAACAAATGATGGTGCTGTTG
ATGGGATGGATCCGAGTTTGATTAGCTTGCCTGCTCATTTCTTCGGAGTCTATGATGGTCATGG
TGGTTCCCAGGTTGCTAACTACTGTCGTGATCGACTTCATCTCGCACTGGTAGAAGGGCTAAG
AAATGTATCTGAAGGTCTGGGAGGAGTAAGCGGGGATGCTTGGAAGAAGCAGTGGGAGAGA
GTGTTCGTTGATTGCTTTCAGAAAGTTGATGATGAGGTGGGAGGCAAAGTTAGCAGAGGGAA
TGTGGGAGGTGCAGCTGATGCATCGGAGGATGGCGGCAATCTTTGTTCAGGTGCGCCATTAGA
ACCTGCTGCCCCCGAAACAGTCGGATCAACAGCTGTAGTTGCTGTTATCTGTTCTTCACATATC
ATCATTGCAAATTGTGGGGATTCCAGGGCGGTCCTTTGTCGTGGCAAACAGGCTGTGCCACTA
TCGGTGGATCATAAA
F: CACTGGTAGAAGGGCTAAGA

R: CTACAGCTGTTGATCCGACT

SnRK2: serine/threonine-protein kinase SRK2

>Cluster-24380.43061;orf1 len=1113 frame:2 start:350 end:1462 gi|743856397|ref|XP_010941849.1| PREDICTED: serine/threonine-protein kinase SAPK10-like [*Elaeis guineensis*]
GAGCGGGGACAGGATTGGGGGATCATGGATCGTGCGGCGCTGACGGTCGGGCCGGCGATGG
ATCTGCCGATAATGCACGACAGCGACCGGTACGAGCTGGTGAGGGACATCGGGTCCGGGAAC
TTCGGGATCGCAAGGCTGATGAGGGATAAGAAGACACGGGAGCTGGTGGCGGTGAAGTACAT
CGAGCGGGGCGAGAAGATTGATGAAAATGTTCAGCGAGAGATAATTAACCACAGGTCTTTGA
GGCACCCTAATATTATTAGGTTCAAAGAGGTTATTCTAACTCCAACCCATTTGGCTATTGTCATG
GAATATGCTTCTGGTGGTGAGCTTTTTGAGCGCATTTGCAATGCTGGCCGCTTCAGTGAGGAT
GAGGCTCGCTTTTTCTTCCAGCAGCTTATATCAGGAGTTAGCTATTGCCACTCTATGCAAGTAT
GTCACCGTGATTTAAAATTGGAGAACACCTTGTTGGATGGAAGCGTGGCTCCTCGTCTGAAGA
TATGTGATTTTGGGTATTCCAAGTCATCTGTTCTGCATTCACAACCAAAATCAACTGTTGGAAC
TCCTGCATACATTGCTCCTGAAGTGCTACTCAAGAAGGAATATGATGGCAAGATTGCTGATGTG
TGGTCTTGTGGAGTGACTTTATATGTGATGTTGGTGGGAGCATATCCTTTTGAGGATCCAGAAG
AGCCCAAAAACTTCCGAAAGACAATACAGCGTATATTAGGAGTTCAGTATTCAATTCCAGACT
ATGTCCACATATCTCCTGAGTGCCGACAACTGATCTCAAGGATTTTTGTTGGCAATCCTGCTAT
GAGGATAACAATCCCTGAGATACAAAATCATGAGTGGTTCTTGAAGAACCTTCCTGCTGATCT
GATGGATGACAACACGATGAGCAACCAATATGAGGAGCCTGACCAACCCATGCAGAGCATTG
ATGAGATAATGCAGATTATAGCTGAGGCAACCATCCCTGCAGCTGGCACTCGTGGGCTGAACC
CATATTTGACAGGCAGCATAGACCTTGATGATGACATGGAGGATCTTGACTCTGATCCTGAGCT
TGATGTGGACAGCAGTGGGGAGATAATCTATGCAATGTGA
F: GCTCCTCGTCTGAAGATATG
R: GCACTCAGGAGATATGTGGA

ABF: ABA responsive element binding factor

>Cluster-24380.39088;orf1 len=1155 frame:-2 start:1649 end:495 gi|743817554|ref|XP_010930969.1| PREDICTED: LOW QUALITY PROTEIN: ABSCISIC ACID-INSENSITIVE 5-like protein 4 [*Elaeis guineensis*]
CCAGATTACCTGACCGCTAAACGGCTATCCGAACCGCCCCGGGCCGGGCCCTATCTTTGCCTT
TTGTGGCAACTCCCGAAACCGCTTCCCGGTATTCTTGAGACCCCGGGGCTTCATAAATTTCCG
CTCGGGTCCCTCGCTTCCGTAGACCAGGCACTCGGAGGGGGAGAGGTCTCCTTCGAGATCGT
TCCATCAAGCGGCGCAAGAAGTGGAGATAAGAGAGACAGAGAGAGAGAGAGATTTGTGATC
GATAACCAAAACCCTAGGCCTGGAGACCCGCGGATGGCGTCGTCGAGGGTGATGCAGTCGTC
CTCGACGGCGAACTCGGATCTCGCGTGCGAGCCCTCCATATATTCTCTCACGATGAGCGGCGA
CCAGGCCAAGAATTTGGGATCCATGAACATGGACGACCTCCTTCGCAACATCTACGGCGACG
GCACAGCTGCGGCGGCGTCGCCGGCTACCCCCTTTGGTGGAGACGCAGGGCCGCCGCTGGCA
CGGGAGGGGAGCTCCTCGCTGCCGATGAGCATCGGGAGCAAGACGGTGGAGGAGGTGTGGA
GGGAGATCTCCGGAGGGAGGAAGGTGGATGGAGGAGGGGATGGATCGGGGTACAAGGACGC
CACGGCCGCGGAAGCGGCGGCAGCGAACGGGGAGATGACGCTGGAGGACTTCTTGGCGAGG
GCGGGGGCGGTGAGGGAGGAGGACGTTAGGGTTCCTCCGGGGTCGGTGGCGGCGGGCGGGT
TTGGGGTGGATGCGGTGATGAATGATCGGTTTAGTCAGCAGCAGGCTCAATTGCCGCTCGAGA
ATCCGATGCTAGGGTTTGGAAATGGGGTGGAGGCTGGGGCTGGGGGAGGAGGGAGAGGAGG
GAGGGGGAGGAAGAGACAGGTGCTCGATCCGGTGGACAGGGCGGCGCTACAGAGGCAGAA
AAGAATGATCAAGAACAGGGAGTCCGCAGCGAGGTCGAGGGAGAGGAAACAGGCTTATACT
GTGGAACTTGAATCTATAGTTACGCACCTGGAGGAGGAAAATGCGAGGCTATTGGGAGAACA
GGAGGAGGTCCACAGGCAGAGGCTTAAACAGCTTCTGGAGAACCTGATCCCAGTTACCGAG
AGGAAGCAGCCCCCTCGTGCTCTGAGGAGAACTCACTCCATGTAG
F: GAAGAGACAGGTGCTCGAT
R: GGTAACTGGGATCAGGTTCT

ETR: ethylene receptor

>Cluster-24380.32167;orf1 len=1944 frame:-1 start:2474 end:531 gi|743768453|ref|XP_010914501.1| PREDICTED: ethylene receptor-like [*Elaeis guineensis*]>gi|743768455|ref|XP_010914502.1| PREDICTED: ethylene receptor-like [*Elaeis guineensis*]>gi|743768457|ref|XP_010914503.1| PREDICTED: ethylene receptor-like [*Elaeis guineensis*]

AGAGGAGAATATTCTGGCTGTGGCGGTTGCTGGGAGATGGAGGGCTGTGACTGCATTGAGCC
GCAATGGCCTGCAGATGAGCTTTTGGTGAAGTATCAGTACATATCGGACTTCTTTATAGCACTT
GCATATTTCTCGATACCCCTTGAGCTCATTTATTTTGTGAAGAAGTCTTCATTCTTCCCGTATAG
ATGGGTGTTAATACAGTTCGGTGCTTTCATTGTTCTTTGTGGAGCAACCCACTTGATTAATTTAT
GGACTTTCACCGTGCACTCGAGAACACTTGCCATAGTAATGACAGTAGCAAAGATTTCAACTG
CCGTTGTGTCATGTGCCACTGCATTGATGCTTGTGCATATAATTCCTGACCTGTTGAGTGTGAA
AACAAGGGAGCTTTTTCTGAAGAATAAGGCTGAGGAGCTTGATAGGGAGATGGGCCTTATAA
GAACACAGGAAGAGACAGGGAGGCATGTCCGAATGCTGACGCATGAAATCAGAAGCACACT
TGACAGGCACACTATATTGAAGACTACACTTGTTGAGCTAGGGAGGACTTTGGATTTGGCAGA
ATGTGCACTGTGGATGCCATCCAATAGTGGTTTAAATCTTCAGCTTTCTCATACTCTGCACCAC
CAAATACCTCTTGGATCCGCCGTGTCTATTAACCTACCAATTGTCAATCAAGTTTTCAGCAGTA
ACCGTGCAATAAGGATTCCGCATACTTGTCCACTAGCAAGGATCCGGCCACTCACAGGAAGAT
ATGTGCCACCAGAAGTTGTTGCTGTCCGTGTACCGCTATTACATCTTTCAAATTTCCAAGTAAA
TGATTGGCCTGAGCTATCTGCAAAAAGCTATGCAATAATGGTTTTGATTCTTCCATCGAATAGT
GCAAGAAAGTGGCATTTCCATGAACTGGAGCTTGTGGAGGTGGTTGCTGATCAGGTAGCAGT
TGCACTTTCTCATGCTGCAATTCTAGAAGAGTCTATGCAGGCACGCAACCTACTCATGGAGCA
AAATGTTGCTCTAGATTTAGCTCGTCGGGAGGCAGAAATGGCAATTCGTGCTCGCAATGATTT
TCTAGCTGTCATGAACCATGAAATGCGGACTCCTATGCATGCAATCATTGCTCTGTCCTCCCTG
CTTCTGGAAACCGAGCTAACGCCAGAGCAACGCTTGATGGTGGAAACTGTATTGAAGAGTAG
TAACCTTCTCGCAACACTCATCAATGATGTTTTAGATCTTTCTAAGCTTGAGGATGGGAGCCTT
GAGCTGGAGATTGCAGCATTCAATCTTCATTCTGTTTTCAGAGAGGTCATTCATTTGATAAAGC
CGATAGCAGCTGTAAAGAAGCTCTCAGTATTGGTTACACTAGCACCTGATCTGCCTTTGTGTGC
CATTGGTGATGAGAAGCGGCTTATGCAAGCTATTTTAAATATATCTGGTAATGCCGTTAAGTTC
ACAAAGGAGGGTCATATATCAATTATGGCTTCTGTTGCGAAACCAGATTCTCTAAAAGACTCTC
GAGCTCCCGAATATTATCCAATTACTAGTGACGGGCACTTCTACTTGCGTGTGCAGGTAAAGG
ATACTGGTTGTGGAATCAGTCCCCAAGATTTACCACACATCTTTACCAAATTTGCACGCTCTCA
ACATGGAGCAAACAAAGGTTATAGTGGAAGCGGACTCGGGCTCGCCATTTGTAAGAGGTTTAT
AAGCCTCATGGAAGGACACATTTGGCTTGAGAGTGAAGGTGTGGGTAAAGGTTGCACAGCAA
CATTTATTGTCAAACTTGGTGTATGTGAAAAATCGAAGAGTCATCTTCAACAAATTGTTCCAGT
GACTGGGTCAAATATTGGAGATGCTGATCTTTCTGGTCCAAGAGTACCCTTTCGAGACGAGAG
TGGTCTGCTTCCCTCCAGGTTCCGGTACCAGAGAAGTGTATAG

F: GAGGAGCTTGATAGGGAGAT

R: GGTGCAGAGTATGAGAAAGC

CTR1: serine/threonine-protein kinase CTR1

>Cluster-24380.12097;orf1 len=198 frame:-3 start:1314 end:1117 gi|672159618|ref|XP_008799574.1| PREDICTED: probable serine/threonine-protein kinase roco5 [*Phoenix dactylifera*]

TCGCAGCCTCAGTGGATGGCGCCGGAAGTCCTCCGCGGCGAATGCTCAAATGAGATGTCGGA
CGTGTTCAGCTTCGGAGTCATACTGTGGGAGCTGATGACGGAGTCCGTCCCGTGGAGCCATCT
CAACTCCCTTCAGGTAAAAAGCCAGCCAGATCTCCTCGATCTCTCTCTCTCTGTTGATGGCCG
GAGGCGGTGA

F: TCGCAGCCTCAGTGGATG

R: TCCGGCCATCAACAGA

BRI1: protein brassinosteroid insensitive 1

>Cluster-24380.58194;orf1 len=3450 frame:3 start:3 end:3452 gi|743754986|ref|XP_010927763.1| PREDICTED: systemin receptor SR160-like [*Elaeis guineensis*]

CCCCCCCCCCACCCCCTCTCTAATGACGGAACTCAAACCCTAGAAACCCTTACCTCAGCCATG
AGTGAAAGAGAACGAGACCTCGACGGCCCATCCATGGACTTCCTCCCTCTTCTCCTTCTGCTG
TTACTACTATTCTCAGGGGTTGCATCGAGCGATGCCGGGGACTTGGAGCTTCTGATGTCGTTCA
AAAGATCGCTCCCCAATCCCCAGGTCCTCCAAAACTGGGACCCGAGCCAGAACCCTTGCTCA
TTCGCCTGCGTCAGCTGCAAGGCTGGTCGCGTCGCCGGCGTCGCCCTCCAGAGCGTCGCCCT
CAGCACCGACTTGCGCTCCGTTTCCTCTTATCTCGTGACCCTCGGGAGTCTAGAGAGTCTCTC
CCTCCGCTCCGCCAACCTCACCGGAAATATCTCGGCGGCGGCGTCCCGGTGCGGCAGCCAGC
TCGCCGTGCTGGACCTCGCAGGTAATGGCCTCGGGGGGTCGGTGGCTGATGTTTTAAACCTCG
CCGCTGCCTGCTCGGGGCTGAGATCCCTCAATCTCTCCGGCAACTCCATTGGGATCCCTTCTG
CCGGGAAAAACCCTTTTGGGTCTGGTGGGTTCTCGCTGGAGGTACTCGACCTCTCCCACAAC
AAGATCTCCGACGAGACCGACCTACGGTGGTTGCTGTCCAGCCTCGGCCTCCTCCGGCAGCT
GGACTTGAGCGGGAACCGGATCACCGGCGGGATCCCAGCGATGAGCACCTGCTCCGGCCTTC
AACACCTGGACCTGTCGGCGAACGAGCTCGCCGGCGCGGTAGGGGTGGGAGTCTTCGGCGG
GTGCCGGAGTTTGAGCTACCTGAACCTCTCTGCCAACCACTTCACCGGTATTCTTCCCTCCGA
CCTCTTTTCGTGCTCTTCCTTGGCCTCCCTTTCACTCTCCAACAACAACTTCTCGGGCGAGTTC
CCCTTCGAGACTCTAGTCTCTTCGATGCCGAAACTCAAGACTCTCGAGCTCTCGTTCAACAAC
TTCTCTGGGCCCCTCCCGGACGCGGTCTCGAAGCTCTCGATGCTGGAGCTTCTGGACCTCAGC
TCCAATGGCTTCTCCGGGTCGATCCCCTCGGCTCTCTGCCAAAGCTATGAAACTGGCTTGAAA
GAGCTCTACCTTCAGAACAACCGGTTCGCCGGTCGCATTCCAGAGTCCCTCCGCAACTGCTCC
AAATTGGTGTCGCTGGACCTCAGCTTCAATTACCTCAGCGGAGCCATTCCCGCCACTCTGGGC
TCTCTGTCTTCTCTCCGCGATCTCATCATGTGGCAGAATTTGCTCGAGGGCGAGATCCCGCCAC
AGCTGTCCAACATCCGGAGCCTGGAGAACCTCATCCTCGACAATAATGGGCTGACGGGATCC
ATCCCCGCCGGCCTCAGCAACTGCACCGATTTGAACTGGATCTCGCTGTCCAGCAACCACCTG
AGCGGCCCCATTCCCTCCTGGATCGGCCGGCTCGGCAATCTCGCCATCCTCAAGCTCGGGAAC
AATTCTTTCTCCGGCCCCATCCCGCCGGAGCTCGGGGACTGTAAGAGCTTGATTTGGCTGGAC
TTGAACGACAACCAGCTCAACGGGACCATCCCGCCGGCCCTCTCCAGGCAGTCGGGCAACAT
CGCCGTCGGCTTGGTCACCGGGAAACGGTATGTGTACCTGAGGAACGACGGGAGCAGCGAG
TGCCGCGGGTCCGGGAACCTGCTGGAGTTCGCGGGAATACGGCCGGAGGAGCTGAACCGGC
TCCCGAGCCGGCGGTTCTGCAACTTCACCAGGCCTTACATGGGGAGCACTCGGTACACTTTCA
ACAACAATGGCTCGATGATCTTCCTCGATCTCTCTTACAACCAGCTGGTCGGCGACATCCCGA
GGGAGCTCGGGAGCATGTACTATCTCATGATCTTGAACCTGGGCCACAATATGCTCTCCGGTCC
GATCCCATCGGACCTGGGTGGCTTGCACTATGTTGGTGTCTTGGATCTTTCTCATAATGCTCTC
GAAGGTCCGATCCCAGCTTCCTTCTCCGGCCTCTCCATGCTGTCCGAGATCGATCTCTCCAATA
ACAAGCTTAATGGGACCATCCCCGAGTTGGGGCAACTGGCCACATTCCCACGGTACCGGTATG
AGAACAATTCCGGCCTCTGTGGCTTCCCCCTCCCATCTTGTGGACAGAGTGCTAATGGCACTG
CAAGCAGCGACCACCGGAGGTCTCGCGGTTGGCGGGGCTCCCTTGCTGGGAGTGTGGCGATG
GGATTACTCTTCTCCCTCTTCTGCATTTTTGGTGCGATCATTATTGCGGTGGAGACCAGGAAGA
GGAAGAGGAAGAAAGAAAACAATAACAGCTCACGAGATTTCTACATCGGTGATAGTCGATCT
CATTCCGGCACTGCCAACTCCAACTGGAAGCTGACGGGCACGGAGGCCATGAGCATCAACCT
TGCCACATTCGAGAAGCCTCTTCGGAAGCTTACATTTGCCGATCTCCTCGAGGCGACCAATGA
CTTCCATGACGACAGCCTAATTGGCTCCGGTGGATTTGGGGATGTCTACAAAGCCCAGCTTAA
GGATGGCAGTGTTGTTGCCATCAAGAAACTGATTCATGTCAGTGGGCAAGGCGACCGGGAGT
TCATGGCCGAGATGGAGACCATTGGAAAGATCAAGCACCGCAACCTGGTTCCTTTGCTGGGC
TACTGCAAGGTTGGAGAAGAGCGGCTTTTGGTCTATGAATACATGAAGTATGGAAGTCTAGAA
GATGTCTTGCACGACCGGAAGAAGGCCGGGATCAAGCTAAATTGGGCAGCAAGGAGAAAGA
TTGCAGTCGGGGCGGCGAGAGGTCTAGCATTCCTCCACCACAACTGCATCCCCCACATAATTC
ACAGGGATATGAAGTCCAGCAACGTCCTTCTTGACGAGAACTTGGAAGCCAGGGTCTCGGAT
TTCGGGATGGCGAGACTTATGAGCGCAATGGACACTCATTTGAGCGTGTCCACCCTTGCAGGC

ACACCAGGATACGTCCCACCGGAGTACTACCAGAGCTTCCGGTGCACCACCAAAGGGGATGT
ATATAGCTATGGTGTGGTCTTGCTTGAGCTACTCACAGGGAGGCAGCCGACAGACTCATCGGA
CTTTGGGGATAATAACTTGGTCGGGTGGGTGAAGCAGCACACGAAGCTGAGAATCACCGATG
TCTTTGATCCAGAGCTATTGAAGGAGGACCCCAATCTGGAGCTGGAGCTGTTGGAGCACCTTA
AGATTGCTTGTGCCTGCTTGGATGACAGGCCATTACGGCGCCCCACGATGCTTAAAGTGATGG
CGATGTTCAAGGAGATTCAAGCAGGATCGACAGTGGATTCCACAACCTCAGCTCCTCCAGCTT
CGGTTGATGGGAGTTTTGGGGTGGTCGATATGAGCCTCAAGGAAGGCAAAGAGGACAAGGAT
TAA
F: CACAGGGATATGAAGTCCAG
R: AGTAGCTCAAGCAAGACCAC

BSK: BR-signaling kinase
>Cluster-24380.25346;orf1 len=1644 frame:1 start:358 end:2001 gi|743855073|ref|XP_010941215.1|
PREDICTED: probable serine/threonine-protein kinase At4g35230 [*Elaeis guineensis*]
AACATCAAATATAGCTATGTAATACGCTCTCTCTCTCTCTCTCTTATTGGACACACTCACTCT
TTCTTATTCTTCTCCACTGCTCTTTCTCTCTCGTCTCCTCTGGCCTCCTTCGCCGCCGCTGCCGC
CGCCGGGAAGCGGATGGGATTCTAGGGTTCGGGCCGCGATGGGCTGCCTCCACTCCAAGACC
TCCAACGTCCACTCCCCCGACGACGACTCCGTCCCTGGCGACAAGCCAGATCTACCGAATGG
AGACGGGGGCTTGCAGGATGAGGTGCCGGCATTCAAGGAGTACGGGCTTGCGGACCTCCGG
GCGGCGACCAAGGGCTTCAGCCCCGAGATGATCGTCTCGGAGAGCGGTGAGAAGGCCCCCA
ACGTCGTTTACCGTGGAAAGCTCGATGGCGGCCGCCCCGTCGCAGTCAAGCGCTTCTCCAAG
CAGTCCTGGCCCGATGCCCAGCAGTTCGTGGCGGAGGCGGCCGGGGTTGGGAAGGTGAGGC
ACAAGAGGCTGGTGAACCTGATTGGATGCTGTGCCGAGGGAGATGAGCGGCTTCTCGTGGCG
GAGTTCATGCCAAATGATACACTCTCCAAGCATCTTTTCCACTGGGATAAGCAGCCACTACCAT
GGGAAATGCGAGTTAGAGTTGCATACTACATTGCACAAGCACTTGATCATTGTAATACTGAAA
ATCGGAAAATCTATCACGATTTAAATGCATATAGAGTTCTTTTTGATGAGGATGGTGACCCTCG
CTTATCTAGCTTTGGTCTCATGAAGAATAGCCGGGATGGGAAAAGTTACAGCACTAACCTAGC
ATACACTCCGCCGGAGTTCTTGCGAACTGGCAGGGTCATTCCAGAGAGTGTAATATACAGTTA
TGGAACAGTTTTGCTGGATCTGTTGAGTGGGAAACATATCCCTCCAAGTCATGCATTAGATCTA
ATAAGAGGGAAGAACATGTTATTGATAATGGATTCATCGCTGGAGGGGCAGTATGCTAATGAA
GACGCAACCCAGCTTGTTGAACTAGCTTCAAAATGTCTACAGTTTGAGGCTAGGGATCGACCT
AATTCCAAATTTCTTCTTTCTGCTGCAGCACCCCTTCAAACACAGAAAGAGGTGGCATCACAT
GTTCTAATGGGCCTGACTAAAACACCGGCGATACTACCAACAATGCTCTCTCCACTTGGAAAG
GCTTGTGCAAGGATGGACCTTACTGCTGTCCATGATATATTGCTTAAAACAGGTTATAAAGATG
AAGAAGGTGCAGAAAACGAGCTTTCCTTTCAAGAATGGACGCAACAAGTGCAAGAGATGTT
GAATACAAAGAAATTTGGTGATATTGCATTCAGAGACAAGGATTTTAAAAGTGCAATCGAGTA
TTATTCGAAGTTGGTGGTGATGATGTCGGTTCCTTCAGCTACTGTTTTTGCTAGACGGGCTCTG
TCATACCTGATGAATGGCCAGCCAGAGCTTGCCCTTCGGGATGCCATGCAAGCACAGGTGTGC
ATGCCTGAGTGGCCCACAGCTTTCTATTTGCAAGCTCTTGCACTCTCCAAGCTCGGAATGGAA
ACCGATGCTCAGGATATGCTGAATGATGGAGCCACATTTGAAGTGAAGAGGCAAAACAGCTG
GCGCGGCTAG
F: GGGTCATTCCAGAGAGTGTA
R: TCCCTAGCCTCAAACTGTAG

BIN2: protein brassinosteroid insensitive 2
>Cluster-24380.50914;orf1 len=1242 frame:-1 start:1816 end:575 gi|672106778|ref|XP_008794094.1|
PREDICTED: shaggy-related protein kinase eta [*Phoenix dactylifera*]
TTGCTGGCCGTGTCGTCTCGGATGGCCTCGCTGCCTCCGCTGGGACCTAATCACCACCCAGAC
CTCCAGGCCTTGAACCTCGCCGCCGCTCCTCGCCGGCCGGAGATGGCCGAAGACAAGGAAG
CGTCTGTTATTGAGGGGAATGGTCCAGTAACTGGTCACATTATCTCAACTACGATTGGAGGAA
AGAATGGCGAAGCCAAACGGACCATTAGTTACATGGCAGAGCGTGTTGTGGGTACTGGGTCA

TTTGGAATTGTCTTTCAGGCTAAATGCTTGGAAACTGGGGAGATTGTTGCTATAAAAAAGGTC
TTGCAGGACAGGCGATACAAAAACCGTGAGCTACAACTGATGCGCTCGATGGATCATCCAAAT
GTGATTTCTCTGAAGCACTGTTTCTTCTCCACCACTAACAGAGATGAACTGTTTCTGAACCTG
GTCATGGAGTATGTCCCTGAAACTGTATTCCGTGTCCTGAAGCATTACAGCAATGTAAAGCAG
AGGATGCCGCTTATCTATGTGAAACTTTATATGTATCAGGTATTTAGGGGGTTAGCATACATTCA
TACTGTTCCAGGAATTTGCCATAGAGATGTGAAGCCACAGAATATCCTGGTCGATCCTCTTACT
CATCAAGTCAAGCTCTGTGACTTTGGAAGTGCCAAAATTCTGGTTAAGGGTGAAGCAAACAT
ATCATACATTTGCTCTCGCTATTACCGTGCTCCAGAGCTCATATTTGGTGCAACAGAATATACAG
CATCTATTGATATATGGTCAGCAGGATGTGTACTTGCGGAGTTGCTTCTTGGCCAGCCATTATTT
CCTGGAGAAAGTGCTGTCGATCAACTTGTTGAGATAATCAAGGTTCTTGGTACTCCGACTCGA
GAAGAAATTCGATGCATGAATCCTAACTATACAGAATTTAGGTTTCCACAGATAAAAGCTCATC
CTTGGCACAAGGTTTTTCACAAGCGAATGCCTCCGGAAGCTATAGATCTTGCATCCCGTCTTCT
TCAATATTCACCAAGCCTCCGTTGCACTGCACTTGAGGCTTGTGCCCATCCTTTCTTTGATGAG
CTACGGGAGCCCAATGCGCGGTTGCCAAGTGGTCGCCCTTTTCCTCCTCTCTTCAACTTTAAA
CAAGAATTAGTAGATGCTTCGCCAGAGCTCATCAACAAGCTGATACCTGAGCATGTGAAGCGA
CAATACGGTCTTAATTTTACACGTCCAGCTGGGACATAA
F: GAACCTGGTCATGGAGTATG
R: TGAGTAAGAGGATCGACCAG

BZR1/2: brassinosteroid resistant 1/2
>Cluster-24380.41344;orf1 len=1155 frame:3 start:3 end:1157 gi|743860987|ref|XP_010943157.1|
PREDICTED: protein BZR1 homolog 1-like [*Elaeis guineensis*]
AAAAAAAAAAAAACAGAGAGAGAGAGAGAGGAAAAGGAAGCAAGGGGGAGCAAAGGGTGA
AGAAAATAAACACACCAAACTTCTCTCCTTTTCGTCTCCTCTCTACCTCCATCTCTCTCCTGTC
CTTCCCAAAACCAAGCAGACGGAGTGGAGGAAAAACAGAGAGAGGGAGGAGAGATTATCAG
GATTGGGGAGAGATAGAGAGGAGAGAGAGAGAGAGGGAGGGATGACGTCGGGAGGGGGAA
GGTTGCCGACGTGGAGGGAGAGAGAGAACAACAAGCGGAGGGAGCGGCGGCGGCGGGCGA
TCGCGGCGAAGATATTCTCGGGGCTGCGGACGCTGGGGAACTACAAGCTCCCCAAGCACTGC
GACAACAACGAGGTCCTCAAGGCCCTCTGTGGCGAGGCCGGCTGGGTCGTGGAGGACGACG
GCACCACCTACCGAAAGGGATGCAAACCAACCCCTCCACCTCCAGAGGCTGCTGCCGGAGGT
CAGTCGACAAACATAAGCCCTTGCTCCTCATCTCATCATCTGAGCCCACCTTCCTCCTCCTTCC
CTAGCCCTGTCCCTTCCTACCATGCCAGCCCATCATCGTCCTCCTTCCCTAGCCCCACCCGAAT
TGAGAATTCCAACCCTGGTGTCAATCCCTCCTATCTCCTTCCCTTCCTCCGGAACCTCTCCACC
CTCCCTCCCCTTCGCATCTCTAACAGCGCCCCGGTTACCCCGCCGCTCTCCTCACCGACCGCC
TCCCGCCCTCCAAAAATTAGGAAACCAGACTGGGACTGTTCTGCATTCCGTCATCCTTTGTTT
GCTGCTTCTGCCCCTGCTAGCCCCACACGCGGTCGCCACCATGGGCACCCTGCCACTATACCA
GAGTGTGATGAGTCTGATGCTTCTACTGTTGATTCGGGACGGTGGGTCAGCTTCCAAATGACT
GCCCCGGCATCGCCGACCTTCAACCTTGTTAACCCGGTCGCCCAAATGCCAGAGACTGTAGTG
GGCCCTGGAGGAGGAGGAATGTCTGATAGGGGACGAGGTGGCATGGAGTTTGAATTCGAGAA
TGGAAGGGTGAAGCCTTGGATGGGGGAGAGAATTCATGAGGTTGGGGTGGAGGATCTTGAGC
TCACATTGGGAGTGGGAAGCAGTGGATCTAAGTGA
F: CAATCCCTCCTATCTCCTTC
R: CATTCCTCCTCCTCCAGG

CYCD3: cyclin D3, plant
>Cluster-24380.59326;orf1 len=1323 frame:3 start:327 end:1649 gi|743808580|ref|XP_010928373.1|
PREDICTED: cyclin-D3-2 [*Elaeis guineensis*]
CGAGAAGCCACTCCCTCACCTTCTTCTCTTCCTTCGTCTTCTCCTGTCCCTCCTCTCACCATTA
CTACTATAACCCCTGCGTATCATGAGCAAAACTTGCCAACCTCTTCTTCCCCTCAGAAGGAGAT
CATGGCTCTCACCTCCCTCCTTGACCCCCTCTACTGCCCAGAAGAGCACTTGGAGCTCGACGA
CGATACCTTTCTACCCTCCCACCTCCCCGTCTCGGTCGCCGAAGTGGCGGCGGAGACGCCGG

AGGAATGGGCGGAGATCTTGTCTTCTCTGGTAGCCAAAGAAGGGGAGACCCACCCCACGCTT
TCCTCTGACGGCGCCGACGATCTCTATCTCCGCTCCGCGAGGAGGGGCGCCGTCGCGTGGGT
GGCGCGCGCCGCCGCCCGGCATGGATTTTCTGCTCTCACGGCGGTGCTCGCCGTGAACTACCT
CGACCGGTGCTTCCTCTCCGGCGGAGGTGGGCTTCGGCTGCAGGGGGATCGGCCGTGGATGG
AGCGGCTGGCGGCGGTGGCGTGCCTGTCCCTGGCGGCGAAGGTGGAGGAGACGTACGTCCC
GCTCCTCCTCGACCTCCAGGCGGCCGCGGCGGAGGCGGACAGGGACCTCGACGCCGGGTAC
GTGTTCGAGGCCAAGACCGTGCGGCGGATGGAGCTCCTCGTGCTCTCCGCCCTCGGCTGGCG
GATGAATCCCGTCACCCCTCTCTCCTTCATCCAGCTCCTCCTCCCCCACCTGTGTTACAAACCC
CAAAACGGTAATCTCACCTCCTCCACCGCCCGCCTCCGCTGCGAAGCCATTCTCCTCTCCGTC
ATAGCGGATGGGAGATGGGTCCGGTATCCGGCATCGGTGTGGGCCGCGGCGGCGATGCGGCA
CGTTTTGGGCCAGCAGCTGGAGCCCGGCAGTGGCGTCGCTGCTGGGGACGCCTTGGAGTGCC
ACGAGACCCACCACCTCTTGGCCTTCCTCAACGCTCCCAAGGTGGGAGAGTGCTTTCAGCTC
ATTCTGGACTGCGTTGAAGGTAGCGGTGGTGTTACTGGTCATGGTCACAAAAGGAAGAACTT
GTTCTCCTCGTTGGATCACTATCGTTCACCATCCAGTCCCAATGCGGTGCTGGGTTCGTGCTTC
AGCTGCGAGAGCTTGTCGAGCTGCGATTCGTGGGCAGTGTGGCCCTTCTCGTCGGCCTCGCC
TTCACCTGAGCTTCCTCCTCCCAAGAGACCCAACTGCATTGCCACCCAAGCCTTTGGAGACA
ACGAGAGTGGGGAGAATGGGGATCGGCTTCACCACCAAGAGCCGAGACTGGATTGCAGTGC
TGTTCGTATTTAA
F: CTCCCTCACCTTCTTCTCTT
R: GGAGGGTAGAAAGGTATCGT

JAR1: jasmonic acid-amino synthetase
>Cluster-24380.22840;orf1 len=1761 frame:-2 start:2150 end:390 gi|743796836|ref|XP_010924828.1| PREDICTED: probable indole-3-acetic acid-amido synthetase GH3.5 [*Elaeis guineensis*]
GTGACATTGGAGAAGATGAGAGTTTTCAGCCTTGAGAGCGTGATAGAGGAGTTCGAGACGCT
GACAAAGGATGCCGGCCGCGTCCAGAGAGAGACCCTTAGAAAGATTCTCGAACAAAATGGT
GATGCAGAGTACCTGCAGAATTTGGGCCTTGGAGGAAGAACCGACCCCGAAAGCTTTAAGGC
TTGTGTTCCCTTGGTCACTCATAAGGATTTGGAGCCCTACATTAAGAGGATCATCGATGGGGAA
ACTTCACCTATTCTCACTGGGAAGCCAATAACATCGATCTCGTTGAGTTCTGGCACCACACAA
GGAAAGCCCAAGTTTTTGCCATTCAACGAGGAACTACTTCAAAATACCATGCAGATCTATAGG
ACTTCATTTGCTTTTAGAAACCGAGAATATCCAATTGGCAATGGAAAAGCCCTACAATTCATCT
ACAGCAGCAAGCAGGTCATAACAAATGGGGGCCTTACCGCTGCAACTGCCACAACAAATGTG
TACCGAAGTGAACAATTCAAATGCACAATGGAGGATATCCATTCTCGGTGTTGTAGTCCTGATG
AAGTTATATTTAGTCCAGACTTCCACCAGTCTTTATATTGTCACCTGCTATGTGGACTAATTTAC
TCGGCTGAAGTGCAGTTTATATCTTCCGCATTTGCCCACAGCATTGTTCATGCATTTCGAATGTT
TGAGCTGGTCTGGGAAGATCTTTGTAGTGATATCAGACATGGAGTTCTCTCCAGCAGAATTAC
TGTCCCATCTATCCGTGCAGCTGTTTGTAAACTTTTAAGTCCAAATCCCATGCTGGCAGATTCC
ATATACAAAAAATGTACGGGTTTAAGTAACTGGTATGGTGTGATTCCAGAACTCTGGCCCAATG
CCAAATATGTCTCTGGCATTATGACAGGATCCATGGAACCATATTTGAAAAAATTGAGACATTA
TGCTGGAAGCTTACCACTTATGAGTGCTGATTACGGTTCTTCAGAGGGATGGATTGCTGCAAA
TGTGAACCCTGGACTGCCTCCAGAATTGACAACCTTTGCAGTGCTTCCCAACATTGGGTACTT
TGAATTTATTCCTCTGGAGAAGCCTGAGGGCCAGGTGCTGGAGAATTGTGCCTCCATTCACTA
TATAGAATCAGAGCCAGTTGGTCTTACAGAAGTTGAGGTTGGCAAAGAGTATGAAATTGTTGT
CACCAGTTTTGCAGGTTTATATCGGTATAGGCTGGGGGATGTTGTAAAGATAGCAAGATTCCAT
AACTCCACACCTGAGCTGCAATTTATATGCCGAAGAAGCCTACTCCTATCAATCAACATTGACA
AAAATACGGAGAAAGACCTGCAGCTGGCTGTGGAAGAGGCAACCAAGCTGTTAGCAGCCGA
GAAGCTCGAGGTCGTGGATTTTACCAGCCATGTCGATGTCTCAACAGACCCTGGCCACTATGT
GATCTTCTGGGAGCTGAGCTCCAGCGCCAGCGAGGAGGTTCTGAGCAGCTGCTGCAATTGCT
TGGACCTGGCTTTTGTGGATGCTGGCTATGTTAGCTCAAGGAAGATTCGTTCCATCGGGCCGC
TGGAGCTCCGCGTACTCAGGAAAGGAACGTTCCAGAAGATTCTCGATCATTATCTAGTCCTCG

GGGCTGCAGTGAGCCAGTTCAAGACACCACGCTTTGTGGGCCCGTCAAACAGCAAGGTCTTG
CAGATCCTGTGCAGGAATGTCGTCGAGAGCTATTTCAGTACTGCCTATGGCTGCTAA
F: CTCGGTGTTGTAGTCCTGAT
R: GGACAGTAATTCTGCTGGAG

COI1: coronatine-insensitive protein 1
>Cluster-24380.36612;orf1 len=1839 frame:1 start:277 end:2115 gi|743867604|ref|XP_010905359.1| PREDICTED: coronatine-insensitive protein 1-like [*Elaeis guineensis*]>gi|743867608|ref|XP_010905360.1| PREDICTED: coronatine-insensitive protein 1-like [*Elaeis guineensis*]
CTGCTGCGCGATCTTGTCTTGATTCTGGGATCTACCGCCGGTGGAGCGAGCCGTCGTCGTCTC
GGAGAGATGGAGCGGCAGAGCTTGAATCGGGTGATGAGCTTGGGGATATCGGACTTGGCGCT
GGAGTGCGTGATGGGGTACATCGACGACCCCCGCGACCGGGAGGCTGTCTCCCTGGTGTGCA
GGAAGTGGTACCGCATCGACGCCATCACCCGGAAGCACATCACCATCGCCATATGCTACTCCG
CCAGCCCCGACCGGCTCCGGCGGCGCTTCCCCCGCCTCGAGTCCCTCAAGCTCAAGGGCAAG
CCCCGGGCGGCCATGTTCAACCTCATACCCGAGGACTGGGGCGGCTACGCCGGCCCCTGGGT
CTGCGAGATCGCCGACGCCTTCAACTGCCTCAAGGCCGTCCACTTCCGCCGGATGATCGTCCA
AGACGCCGATATCGGTGTCCTCGTCAAGGCTAGGGGCCACATGCTCCAGGCGCTCAAGCTCG
ACAAGTGCTCGGGGTTCTCCACCGATGGCCTCTTGCTTGTGGCCCGCTCTTGCAGATGCCTTA
GAACTTTATTTTTGGAAGAAAGCTCTGTTACCGAGAATGATGGTGAATGGCTTCATGAGCTTG
CGGTCAATAATTCTGTTCTTGAGACTTTGAATTTCTATATGACAGAACTTAGAATCTCGCCACA
AGAACTCGAGCTACTAGCCAGGAACTGCCGGTCTTTGATTTCCTTGAAGATCAGTGAGTGTGA
TGTTTCTGATCTAGTTGGCTTTTTCCGCACAGCAACAGCACTTGAAGAGTTCGGTGGGGGCTC
ATTTAATGATCAAGCAGGAGAAATCAACAGGTATGAGAGGGTTTGCTTTCCTCCCAGGCTATG
CTGCTTGGGTCTCAGTTATATGGGGATGAATGAAATGCACATAATATTTCCATTTGCTGCTTCAT
TAAAGAAGCTAGACCTGCAGTATACTTTCCTCAGCACTGAAGATCATTGTCAGCTGATTCAAC
GGTGCCCCAATCTAGAAATTCTTGAGGTGAGAAATGTTATTGGAGACCGAGGATTAGAAGTTG
TTGCTCAGACATGTAAGAAACTTCGAAGGCTCAGAATAGAGCGAGGGGATGATGAACAAGGC
CCAGAGGATGAACAGGGTAGAGTTTCACAGATAGGGCTGTCGAGTCTAGCTCGGGGCTGTCC
TGAACTGGAATATATCGCAGTATATGTATCTGATATCACTAATGCAGCTCTGGAATCTGTTGGCA
CTTTCAGCAAAAACCTCTGTGATTTTCGTCTCGTCTTGCTTGATAGAGAAGAGATGATAACAG
AGTTACCCCTCGACAATGGGGTTCATGCTTTGTTGAGAGGTTGCACCAAGCTTAGAAGGTTTG
CTTTTTATCTGAGACCTGGAGGTCTTTCAGATGTGGGTCTTGGTTTCATTGGCGAGTGCAGTAG
GAATATCCGGTGGATGCTATTCGGTAATGTTGGGGAATCTGATGTTGGACTTCTGCGATTTGCA
AGAGGTTGCCCAAGCCTGCAGAAGCTGGAGCTGAGAAGCTGTTGCTTTACCGAGCGTGCTTT
GGCCCTTGCAGCAACACAGCTGCCTTCATTGAGATACTTATGGGTGCAGGGTTACAAGGCATC
ACCAACTGGCAGGGACCTTATGGCCATGGCCCGCCCATTCTGGAACATCGAATTCATCCCTTC
TAGACAACAGATTGTTTATGATGATCTTGACGATAGGCCAGGTGCAATAGAGAGTCAGGCTCA
AATACTCGCATACTACTCCCTTGCTGGAAAGAGGACCGATTGTCCGGAATCTGTGGTTCCTTTG
TATCCAGAATGA
F: GACAATGGGGTTCATGCT
R: CCTGCACCCATAAGTATCTC

NYC2: transcription factor MYC2
>Cluster-24380.55318; len=948 start:1 end:947
CCTCATGGTCCAACAAACCCCACGTCCCCTTTCAGCGTCCCGATCCTCTCTCTCCTCCTCCTCG
CCCTCTCTTTCCCGGTCCCTTCCTCGCCGCCGAACCCTCGTTTTAGAAGCCCTAAAGCCGCGT
GCCACAATCCTAATCATAGTGGAGTGCAGCTCTGGTCCCATCCCGGAGCCGAGAGTTACCACG
GTTACCCTGCCGGCACGATCXCCGTTGATGTGACGGAGCCCGAGCACCGCCGCCCCCTAGAT
GAACCTGTGGGCCGACGACAACGCCTCTATGATGGAGGCCTTCATGGCCTCTACCACCGACCT
CCAGGGCTTCCCCTGGGCTCCGCCACCTGCTGCGTCGGCGGCTGGAGGGTGTCACGTGCCCT

CATCCCAGTCCGCCGTCATGTCATCGGCGGCGGCGGCGCCGCCCGCACCAGCTATTTTAACCA
GGAGACCCTCCAACAGCGGCTCCTGACACTGATCGAGGGGGCGAGGGAGACATGGACCTAC
GCGATCTTCTGGCAATCGTCTGTTGACGTGGCCTCCGGCGTGTCGCTGCTCGGCTGGGGCGAC
GGCTACTACAAGGGATGCGAGGAGGACAAGCGGAAGCAGAAGACCACCAACCCCGCCACCG
CTGCGGAGCAGGAGCACCGGAAGCGGGTGCTCCGGGAGCTTAACTCCCTCATTTCCGGTGGC
GCCGGCGGGTCCTCGCCCGACGAGACCGTGGAGGAGGAGGTCACGGACACGGAGTGGTTTT
TTCTCGTGTCCATGACGCAGTCGTTTGTCAACGGCGCCGGACTCCCTGGCCAGGCCTTCTTTG
CCGATGCTCCGAACTGGATCGCTGGCGCGGACCGGCTGGCGTTGGCGCCCTGCGATCGAGCG
CGGCAGGCGCGGGAGCTCGGCCTCCAGACCATGGTCTGCGTCCCGGTGGGGACTGGTGTGCT
GGAGCTCGGATCX
F: ATCCTCTCTCTCCTCCTCCT
R: TCATAGAGGCGTTGTCGT

TGA: transcription factor TGA
>Cluster-24380.48939;orf1 len=1434 frame:3 start:432 end:1865 gi|743786123|ref|XP_010922090.1|
PREDICTED: transcription factor HBP-1b(c38)-like [*Elaeis guineensis*]
GTGGTGGTGGAGGTAAGAGGACGATGGTGGTGGATGGGGAGTAGAAGAGGTGGTGCTGGTG
TGGAAGATGTTAAGGAGGCAGCGCCAGGGATGCCGAGCTTCGTTTCATCGCTGCCTGGTGCT
CATCAGATTGAGACAGAGGGAAACAGCATGCAACCTTCTCGAGTTTCTGGCCTTGCAGCCATT
GAGCAGTCTATTGGCTTTCGCATAGAGGATGCAGTTGATCTAAGCAGAAATCCTTTGTTTAACC
CAAAATCAAGTGGCCAATCTTCTGACCCTCTTCAATTTGGTGCTTTTAGTAAGCCTCTTGCTTC
TACTGATATTACTCCAGCGGCAGCTAGAATAGGGTCACTTGCAGTTCTGCAACAAAAAGGGCA
GCAGCCTAATCTGGTGTCATCATCCAGCGGTCACATTGAGAACTGGGGTGAATCTACAATGGG
AGATGCTAGTCCTAAGACTGACACATCAACAGATGTGGATACTGATGACAAGAATCAGAGGCT
TGAAAAAGGACAGGCTGCTGTTGCTGCGGCTTCTGACTCTAGTGACAAATCAAAGGATAAAT
TGGACCAGAAGACACTTCGCCGGCTGGCTCAAAATCGTGAAGCTGCTAGAAAAAGCCGGTTA
AGGAAAAAGGCATATGTGCAACAACTAGAGAGTAGCAGGTTAAAGCTTAATCAACTTGAGCA
GGAGCTTCAGCGAGCACGCCAACAGGGCATCTTCATTTCAAGCTCAGGAGATCAGTCCCATT
CCATGGGTGGAAATGGGGCTTTGGCATTTGACATGGAATATGCACGATGGCTAGAAGAACACC
ATCGACAGATCAATGAGCTGAGGGCTGCAGTGAACTCACATGCAAGTGACAATGACCTCCGT
GCTATTGTTGATAGCATTATGGCACATTATGATGAAATTTTTAGGCTCAAAAGTGTGGCAGCCA
AAGCAGATGTTTTTCACATGTTGTCAGGAATGTGGAAGACGCCCGCGGAAAGGTGTTTTCTGT
GGCTTGGGGGTTTCCGGTCATCTGAACTTCTCAAGCTACTTGCAAATCAGCTGGAGCCTCTTA
CAGAGCAGCAGTTGGTGGGGATATGTAATCTACAGCAGTCCTCCCAGCAGGCTGAGGATGCA
CTGTCACAGGGAATGGAAGCGCTGCAGCAATCCTTGGCAGAAACTTTGGCTTCTGGATCGCT
TGGGCCCACAGGATCATCTGGTAATGTAGCAAATTACATGGGTCAGATGGCAATGGCCATGGG
AAAGCTTGGCACTCTTGAAAATTTCCTCCGCCAGGCCGACAATTTGCGTCAACAAACCCTACA
ACAAATGCTTCGCATATTAACAACACGCCAATCAGCCCGTGCACTCCTTGCCATACATGACTAC
TTTTCTCGACTCCGTGCATTAAGCTCGCTTTGGCTTGCTCGACCAAGAGAGTGA
F: GCTAGAAGAACACCATCGAC
R: ACCAACTGCTGCTCTGTAAG

Supplementary Table 1. Statistics of the assembly quality.

Type	Transcripts	Unigenes
Total number	148,731	148,695
200-500 bp	40,724	40,688
500-1,000 bp	46,360	46,360
1-2 kbp	35,244	35,244
> 2 kbp	26,403	26,403
Minimum length	201	201
Mean length	1,230	1,230
Medium length	811	811
Maximum length	17,548	17,548
N50	1,862	1,862
N90	553	553
Total nucleotides	182,963,763	182,955,236

* *N50* = Sorting the transcript length and adding the transcript length from highest to lowest till the accumulation is greater than 50% of the total transcript length, the last transcript length we added is the N.50.

* *N90* = Sorting the transcript length and adding the transcript length from highest to lowest till the accumulation is greater than 50% of the total transcript length, the last transcript length we added is the N.50.

Supplementary Table 2. Gene expression of hormone signaling pathways and starch and sucrose metabolism determined via qRT-PCR.

No.	Gene	cDNA1 (A)	cDNA2 (B)	cDNA3 (C)	cDNA4 (D)	cDNA5 (E)	A-B	A-C	A-D	A-E	B-C	B-D	B-E	C-D	C-E	D-E
1	PE	9.36 ± 0.11	9.72 ± 0.46	9.73 ± 0.14			ns	ns			ns					
2	GAE	9.94 ± 0.15	10.69 ± 0.09	11.04 ± 0.32			ns	0.0219			ns					
3	xynB	8.57 ± 0.09	9.38 ± 0.17	10.23 ± 0.22			0.0028	<0.0001			0.0023					
4	TPP	11.00 ± 0.27	11.82 ± 0.13	13.01 ± 0.46			0.0413	0.0006			0.0087					
5	SS	8.96 ± 0.12	9.20 ± 0.31	10.16 ± 0.20			ns	0.0017			0.0052					
6	PGM	13.13 ± 0.15	10.77 ± 0.50	12.31 ± 0.13			0.0002	0.0406			0.0022					
7	UGDH	8.83 ± 0.09	9.49 ± 0.13	10.15 ± 0.29			0.0118	0.0003			0.0123					
8	SPS	8.63 ± 0.04	8.86 ± 0.23	9.57 ± 0.19			ns	0.0016			0.0070					
9	MGAM	7.85 ± 0.07	9.19 ± 0.11	9.60 ± 0.14			<0.0001	<0.0001			0.0127					
10	TPS	8.44 ± 0.22	9.54 ± 0.07	11.05 ± 0.56	2.01 ± 1.19		ns	0.0061	<0.0001		ns	<0.0001		<0.0001		
11	ARF	9.19 ± 0.14	9.85 ± 0.14	10.59 ± 0.49			ns	0.0032			ns					
12	CRE1	8.32 ± 0.04	9.15 ± 0.56	10.61 ± 0.59			ns	0.0025			0.0222					
13	B-ARR	8.49 ± 0.21	9.10 ± 0.37	9.99 ± 0.55			ns	0.0089			ns					
14	PYR-PYL	11.66 ± 0.16	12.17 ± 0.09	13.37 ± 0.48			ns	0.0009			0.0057					
15	BAM	17.96 ± 0.30	15.46 ± 0.42	15.34 ± 0.36	0.64 ± 0.62	3.33 ± 1.06	0.0043	0.0031	<0.0001	ns	ns	<0.0001	<0.0001	<0.0001	<0.0001	0.0025
16	GDE		14.16 ± 0.94	14.44 ± 1.53			ns (t-test)									
17	GlgB	15.11 ± 1.20	12.51 ± 0.18	11.21 ± 0.19			0.0099	0.0013			ns					
18	SnRK2	11.23 ± 0.27	9.90 ± 0.22	11.39 ± 0.18			0.0009	ns			0.0005					
19	ETR	8.66 ± 0.22	8.94 ± 0.40	10.31 ± 0.21	2.05 ± 0.21	4.06 ± 0.58	ns	0.0015	<0.0001	<0.0001	0.0056	<0.0001	<0.0001	<0.0001	<0.0001	0.0003
20	CTR1	8.63 ± 0.49	9.22 ± 0.19	11.27 ± 0.20			ns	0.0001			0.0006					
21	BRI1	9.13 ± 0.10	9.39 ± 0.14	11.72 ± 0.86			ns	0.0019			0.0033					
22	BSK	17.16 ± 0.60	13.67 ± 0.11	15.85 ± 0.39			0.0001	0.0214			0.0019					
23	BIN2	15.14 ± 0.53	12.76 ± 0.12	12.81 ± 0.40	-1.31 ± 0.16	1.36 ± 0.24	<0.0001	<0.0001	<0.0001	<0.0001	ns	<0.0001	<0.0001	<0.0001	<0.0001	<0.0001
24	CYCD3	11.28 ± 0.26	11.32 ± 0.17	13.25 ± 0.10			ns	<0.0001			<0.0001					
25	JAR1	8.87 ± 0.07	8.78 ± 0.13	9.98 ± 0.36			ns	0.0024			0.0016					
26	COI1	9.32 ± 0.11	9.87 ± 1.09	10.86 ± 0.05			ns	ns			ns					
27	TAG	13.95 ± 0.37	12.42 ± 1.98	13.93 ± 0.25			ns	ns			ns					
28	GAUT1	10.89 ± 0.10	12.02 ± 1.08	12.94 ± 0.12			ns	0.0175			ns					
29	E.3.2.1.67	8.09 ± 0.10	8.67 ± 1.44	10.24 ± 0.03			ns	0.0442			ns					
30	TIR1	12.69 ± 0.13	10.53 ± 0.29	10.27 ± 0.13	-4.46 ± 0.22	-1.89 ± 0.14	<0.0001	<0.0001	<0.0001	<0.0001	ns	<0.0001	<0.0001	<0.0001	<0.0001	<0.0001
31	AUXIAA	18.81 ± 0.76	12.00 ± 0.14	12.36 ± 0.58			<0.0001	<0.0001			ns					

Supplementary Table 3. Primer sequences for genes associated with plant hormone signal transduction and starch and sucrose metabolism.

No.	Gene names	Primer names	Sequences	Bases
**Cell Wall Biosynthesis and Modification (7 genes)**				
1	*GAUT1* : 2.4.1.43: alpha-1,4-galacturonosyltransferase	GAUT1-F	CCTCTACCACTACGTCCTCA	20
		GAUT1-R	ACCTCAGGTAGTCCAGCAG	19
2	*PE* : 3.1.1.11: pectinesterase	PE-F	GGAAGGAACACTCAAGGAC	19
		PE-R	AACTGCTCTGCTTCTCTGAC	20
3	*E3.2.1.67* : 3.2.1.67: galacturan 1,4-alpha-galacturonidase	E3.2.1.67-F	CTTCGGTCAAGTTCGTTG	18
		E3.2.1.67-R	AGAGGACTTGCCTGATCC	18
4	*GAE* : 5.1.3.6: UDP-glucuronate 4-epimerase	GAE-F	GTCGACACTTCCTCCAAGTA	20
		GAE-R	CAAGGATGGATCGTAGTAGG	20
5	*xynB* : 3.2.1.37: xylan 1,4-beta-xylosidase	xynB-F	GACATACCGGTTCTATACGG	20
		xynB-R	GAACTCTCAGATGGATGTCG	20
6	*Uxs* : 4.1.1.35: UDP-glucuronate decarboxylase	uxs-F	CCAGAAGTTTCCAGTACGTC	20
		uxs-R	GTCAGAGACCATGAGAGGAA	20
7	*UGDH* : 1.1.1.22: UDPglucose 6-dehydrogenase	UGDH-F	CTAACAACCAACCTGTGGTC	20
		UGDH-R	TTCCAGTAGTTAGCCACCTC	20
**Sucrose Metabolism (5 genes)**				
8	*SPS* : 2.4.1.14: sucrose-phosphate synthase	SPS-F	CGGAGACTATTCGTGATAGC	20
		SPS-R	GACCTCACTTCCACTACTGC	20
9	*SUS* : 2.4.1.13: sucrose synthase	SUS-F	GATACAGCTGAACGAGTGGT	20
		SUS-R	TCGGGTATCAACCTAGTCAC	20
10	*FRK* : 2.7.1.4: fructokinase	FRK-F	GTTGCTTCTTGTCACTGAGG	20
		FRK-R	GCAAACCTAAGGGCTTCTAC	20
11	*TPP* : 3.1.3.12: trehalose 6-phosphate phosphatase	TPP-F	GACGTGTAGGGACCATTCTA	20
		TPP-R	CTGAGAGTGCTTCGACTTCT	20
12	*SS* : 2.4.1.21: starch synthase	SS-F	TGGTGACACAGAAGTCTCAG	20
		SS-R	CACTACCATCACACGATGAC	20
**Starch Metabolism (6 genes)**				
13	*GlgC* : 2.7.7.27: glucose-1-phosphate adenylyltransferase	GlgC-F	CTCCTCCTCTTTCCACTTCT	20
		GlgC-R	CCATAAGCTCGTGAAAGG	18
14	*PGM* : 5.4.2.2: phosphor-glucomutase	PGM-F	GGACATCCAGACCCTAATCT	20
		PGM-R	AGCACCACTAGTTGGCATAG	20
15	*glgP* : 2.4.1.1: glycogen phosphorylase	glgP-F	CTGAGAAACTGGCAGAACTC	20
		glgP-R	ATCCTTTCCTTGGCACTC	18
16	*BAM* : 3.2.1.2: beta-amylase	BAM-F	CACCACTCTCTCTCCAACTC	20
		BAM-R	GAGGAAGAGGGATTATCGAG	20
17	*GDE* : 2.4.1.25: glycogen debranching enzyme	GDE-F	AAGGACTTCTGGAAGGAGAG	20
		GDE-R	GGCACACTACCAGTAACACA	20
18	*GlgB* : 2.4.1.18: 1,4-alpha-glucan branching enzyme	GlgB-F	ATGGTGGCTAGAGGAGTACA	20
		GlgB-R	CCTACACCACCATCTTGAAC	20
**Sugar Metabolism and Stress Response (1 genes)**				
21	*TPS* : 2.4.1.15: trehalose 6-phosphate synthase	TPS-F	GTCCATGACTACCACCTCAT	20
		TPS-R	AACCCCTCTTGGACTCATAC	20

Supplementary Table 3. Primer sequences for genes associated with plant hormone signal transduction and starch and sucrose metabolism. (Count.)

No.	Gene names	Primer names	Sequences	Bases
**Starch and Glucan Breakdown (2 genes)**				
19	*MGAM* : 3.2.1.20: maltase-glucoamylase	MGAM-F	GAGAGCAACATTCACAGAGG	20
		MGAM-R	CATATGTAGGTGCTCCCAGT	20
20	*BGL* : 3.2.1.21: beta-glucosidase	BGL-F	CACCTCCTACTCCTCCTCTT	20
		BGL-R	CCTGGGATCTTAACGAAGAC	20
**Glycolysis and Energy Metabolism (1 genes)**				
22	*GPI* : 5.3.1.9: glucose-6-phosphate isomerase	GPI-F	CTACCAGCTAATCCATCAGG	20
		GPI-R	AAGACTGCACATACCTGTCC	20
**Auxin signaling pathway (4 genes)**				
23	AUX1: auxin influx carrier	AUX1-F	CACAACTACCGGATATGGTC	20
		AUX1-R	GTCAGCGTGAACACGTATAG	20
24	TIR1: transport inhibitor response 1	TIR1-F	CAACTGCTACGCTATCTCC	19
		TIR1-R	CAATGAGCTCGAAGCAGT	18
25	AUXIAA: auxin-responsive protein IAA	AUXIAA-F	CCTATGATGGCTACGAGAAG	20
		AUXIAA-R	CAGACTGCTCCCAAAGATAG	20
26	ARF: auxin response factor	ARF-F	GCAAGGAAGTGTTGTAGGC	19
		ARF-R	GATAACAGAGGCAGGTGCTA	20
**Cytokinin signaling pathway (2 genes)**				
27	CRE1: arabidopsis histidine kinase 2/3/4 (cytokinin receptor)	CRE1-F	GAGCAGTTCTGGATGATGTC	20
		CRE1-R	GTCTTCTATCCACCACAGGA	20
28	B-ARR: two-component response regulator ARR-B family	B-ARR-F	AGGGGATACCTCTCTGTCAT	20
		B-ARR-R	CTACCAAGTTCTGCACCTTC	20
**Abscisic acid (ABA) signaling pathway (4 genes)**				
29	PYR/PYL: abscisic acid receptor PYR/PYL family	PYR/PYL-F	GACCGTCCTCAGATCTACAA	20
		PYR/PYL-R	GTAGGATTCCAGCACCACT	19
30	PP2C: protein phosphatase 2C	PP2C-F	CACTGGTAGAAGGGCTAAGA	20
		PP2C-R	CTACAGCTGTTGATCCGACT	20
31	SnRK2: serine/threonine-protein kinase SRK2	SnRK2-F	GCTCCTCGTCTGAAGATATG	20
		SnRK2-R	GCACTCAGGAGATATGTGGA	20
32	ABF: ABA responsive element binding factor	ABF-F	GAAGAGACAGGTGCTCGAT	19
		ABF-R	GGTAACTGGGATCAGGTTCT	20
**Ethylene signaling pathway (2 genes)**				
33	ETR: ethylene receptor	ETR-F	GAGGAGCTTGATAGGGAGAT	20
		ETR-R	GGTGCAGAGTATGAGAAAGC	20
34	CTR1: serine/threonine-protein kinase CTR1	CTR1-F	TCGCAGCCTCAGTGGATG	18
		CTR1-R	TCCGGCCATCAACAGA	16
**Brassinosteroid signaling pathway (5 genes)**				
35	BRI1: protein brassinosteroid insensitive 1	BRI1-F	CACAGGGATATGAAGTCCAG	20
		BRI1-R	AGTAGCTCAAGCAAGACCAC	20
36	BSK: BR-signaling kinase	BSK-F	GGGTCATTCCAGAGAGTGTA	20
		BSK-R	TCCCTAGCCTCAAACTGTAG	20
37	BIN2: protein brassinosteroid insensitive 2	BIN2-F	GAACCTGGTCATGGAGTATG	20
		BIN2-R	TGAGTAAGAGGATCGACCAG	20

Supplementary Table 3. Primer sequences for genes associated with plant hormone signal transduction and starch and sucrose metabolism. (Count.)

No.	Gene names	Primer names	Sequences	Bases
**Brassinosteroid signaling pathway (5 genes)**				
38	BZR1/2: brassinosteroid resistant 1/2	BZR1/2-F	CAATCCCTCCTATCTCCTTC	20
		BZR1/2-R	CATTCCTCCTCCTCCAGG	18
39	CYCD3: cyclin D3, plant	CYCD3-F	CTCCCTCACCTTCTTCTCTT	20
		CYCD3-R	GGAGGGTAGAAAGGTATCGT	20
**Jasmonic acid signaling pathway (3 genes)**				
40	JAR1: jasmonic acid-amino synthetase	JAR1-F	CTCGGTGTTGTAGTCCTGAT	20
		JAR1-R	GGACAGTAATTCTGCTGGAG	20
41	COI1: coronatine-insensitive protein 1	COI1-F	GACAATGGGGTTCATGCT	18
		COI1-R	CCTGCACCCATAAGTATCTC	20
42	NYC2: transcription factor MYC2	NYC2-F	ATCCTCTCTCTCCTCCTCCT	20
		NYC2-R	TCATAGAGGCGTTGTCGT	18
**Salicylic acid (SA) signaling pathway (1 gene)**				
43	TGA: transcription factor TGA	TGA-F	GCTAGAAGAACACCATCGAC	20
		TGA-R	ACCAACTGCTGCTCTGTAAG	20
